# A Three-Step Catalytic Asymmetric Sequence from Alkynes to α-Silyloxyaldehydes and Its Application to a C22–C41 Fragment of Bastimolide A

**DOI:** 10.1021/acs.orglett.4c01310

**Published:** 2024-05-16

**Authors:** Jacob N. Hackbarth, Gregory K. Friestad

**Affiliations:** Department of Chemistry, University of Iowa, Iowa City, Iowa 52242, United States

## Abstract

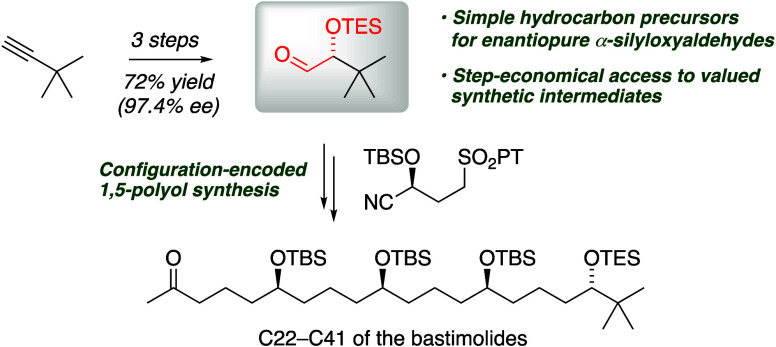

1,5-Polyol structures present challenges in stereocontrol, configurational assignment, and diastereomer separation; these are all compromised by remote stereochemical relationships. A configuration-encoded approach with alcohol configurations previously established within enantiopure building blocks offers a versatile solution to these issues. The iterative construction begins with α-silyloxyaldehydes; here, we introduce an enantioselective and step-economical route from alkynes to α-silyloxyaldehydes via silyl cation-induced ring opening of enol ester epoxides. This development enables an efficient configuration-encoded synthesis of the C22–C41 fragment of the bastimolides.

The World Health Organization reported an increase of 5 million cases of malaria worldwide from 2021 to 2022, rising to a total of 249 million cases; 608,000 deaths were observed in 2022, mostly among small children.^1^ Meanwhile, the development of drug resistance in the malaria parasite *Plasmodium falciparum* and its relatives threatens existing clinical treatments. Combatting resistance requires continued effort in discovery and development of new drugs with novel modes of action.^2^ Recently, the polyketide bastimolides A and B (Scheme 1), which differ only in their ring size (bastimolide B is lactonized at the C23 hydroxyl), were isolated from marine cyanobacteria and showed notable activity (A: IC_50_ 80–270 nM) against several drug-resistant strains of *P. falciparum*.^3^ Among historically important antimalarials and newer investigational drugs, macrolide-type polyketides have untapped potential, and represent a dramatic departure from existing drug structures.^4^ This may suggest an as-yet undiscovered mechanism of action for bastimolides; therefore synthesis is a high priority to support further biological evaluation.^5^

**Scheme 1:**
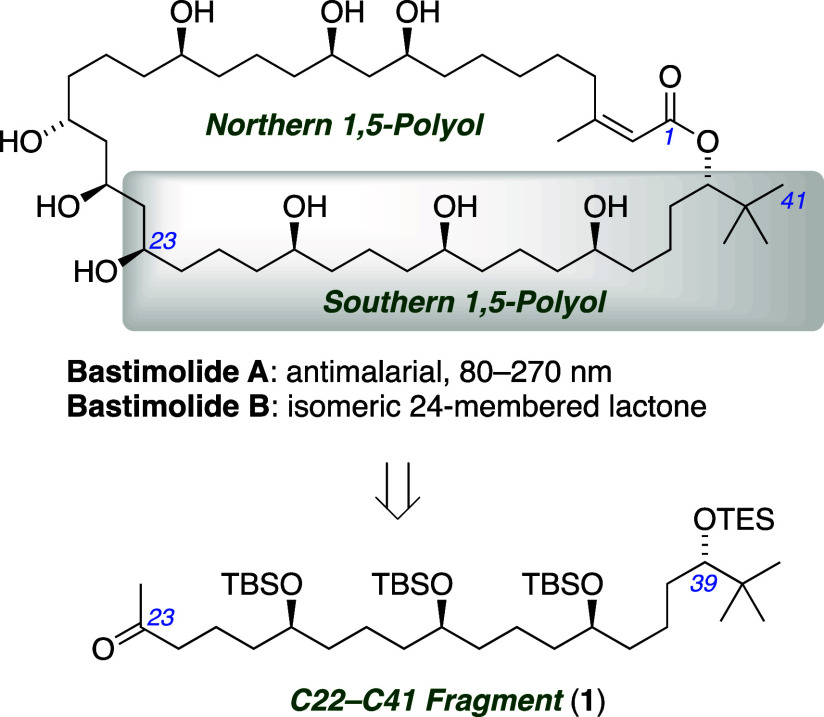
Bastimolide and Its C22–C41 Fragment

The isolated stereogenic centers of 1,5-polyols such as the bastimolides present significant challenges in synthesis as well as structure determination and diastereomer separation.^6^ To address these issues, we introduced a synthesis of 1,5-polyols with innovation at the strategy level, using an *iterative coupling of configuration-encoded building blocks* (Scheme 2a).^7,8^ The coupling events entail iterative Julia–Kocienski olefinations^9^ of aldehydes with enantiopure sulfone building blocks bearing protected alcohols of the required configurations to suit the target. The coupling events are independent of the hydroxyl group configurations, allowing for the stereochemically unambiguous assembly of all stereoisomers of various 1,5-polyols. This strategy avoids not only stereocontrol issues during assembly but also analytical and separations problems associated with the isolated stereogenic centers postassembly. Applying our strategy to synthesize bastimolide A and its analogs will test these assertions, further validating the strategic innovation as well as advancing our medicinal chemistry objectives.

**Scheme 2:**
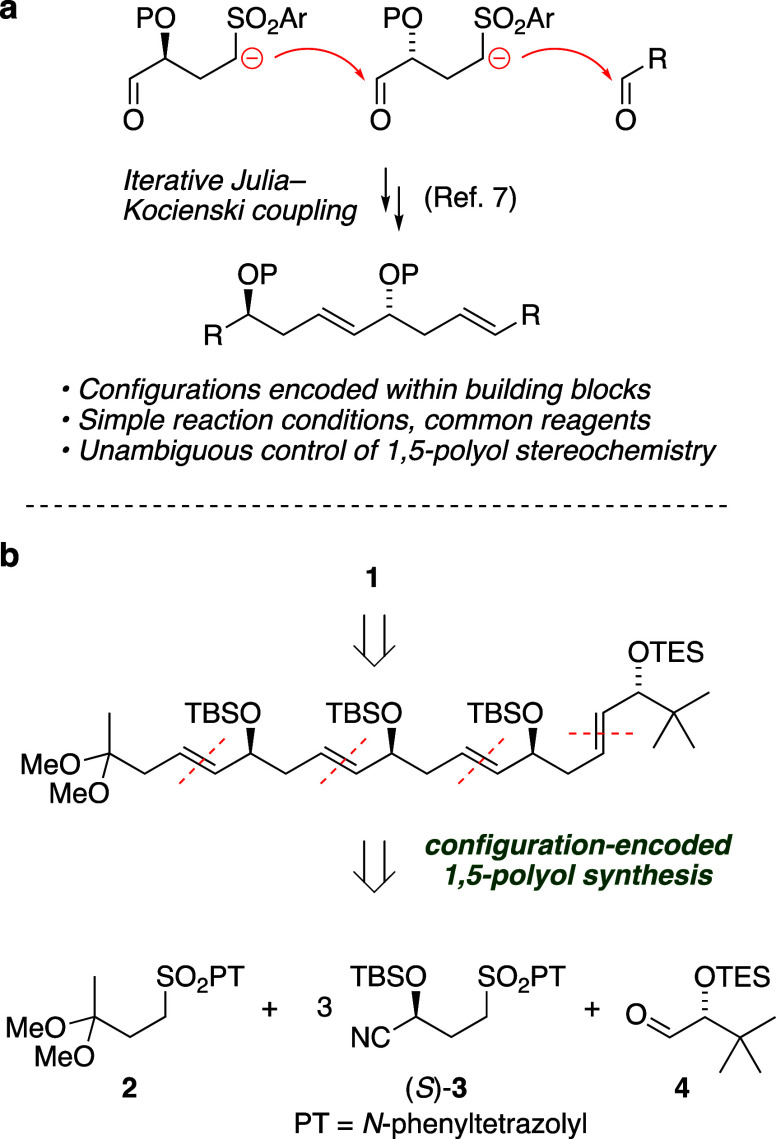
Application of Configuration-Encoded 1,5-Polyol Synthesis Strategy

Retrosynthetically, bastimolides may be divided into two fragments of similar complexity by envisioning an *anti*-selective Mukaiyama aldol coupling^10^ at C22–C23 (Scheme 1), and synthetic efforts first addressed the southern C22–C41 fragment (**1**). Functional group addition of alkenes enables Julia–Kocienski disconnection of **1** (Scheme 2b) to the repeating units of **3**(7a) in which a nitrile serves as a latent aldehyde. The initial coupling would require α-silyloxyaldehyde **4**, which we hypothesized could be accessed from *tert*-butylacetylene in a three-step route, exploiting a ring-opening reaction of enol ester epoxides we introduced in racemic form.^11^ Rendering that route enantioselective was a crucial element in our synthetic plan. Here we report a catalytic asymmetric method to prepare α-silyloxyaldehyde **4** and its application in the configuration-encoded synthesis of a C22–C41 fragment of bastimolide A.

Protected enantiopure α-silyloxyaldehydes are attractive precursors for synthesis of various chiral alcohols,^12^ but their preparations have significant limitations. They can be obtained from reduction of nature-derived enantiopure α-silyloxyesters,^12a,12b,13^ oxidative olefin cleavage of enantiopure allylic^12c,12d^ or allenic^12e^ alcohols, hydrolytic kinetic resolution of racemic epoxides,^12g^ or reduction of *O*-silylcyanohydrins.^14^ These approaches generally suffer from either a lack of versatility due to structural limitations of source materials or poor step economy arising from multistep chiral reagent preparations, redox adjustments, and protecting group manipulations. As we previously reported,^11^ epoxides of (*Z*)-enol esters, on exposure to a silyl cation equivalent (e.g., TBSOTf), undergo both ring opening of the epoxide and protecting group installation in the same transformation, efficiently furnishing racemic α-silyloxyaldehydes.^7a,11^ A key question remained: Would the configuration of an enantiopure epoxide be retained during this transformation?

Our initial attempt to answer this question began with (*Z*)-enol ester **6** (Scheme 3a). Anti-Markovnikov addition of *p*-anisic acid to *tert*-butylacetylene (**5**) furnished **6** in 78% yield via a modification of the Ru-catalyzed method of Dixneuf;^15a^ generation of the Ru catalyst *in situ* gave a somewhat lower yield.^15b,16^ Berkessel–Katsuki Ti-catalyzed epoxidation^17^ with ligand **A** (Scheme 3b) performed well at low catalyst loading (0.3 mol %), providing known enol ester epoxide **7a**(17a) with high enantioselectivity (98.5% ee). Employing additive C_6_F_5_CO_2_H gave improved yield and rate;^17c^ the epoxidation was complete in less than 18 h with 93% yield. Berkessel has noted that carboxylate anions participate in Ti coordination, serving as surrogate ligands to replace hydrogen peroxide under catalytically relevant conditions.^17d^

**Scheme 3:**
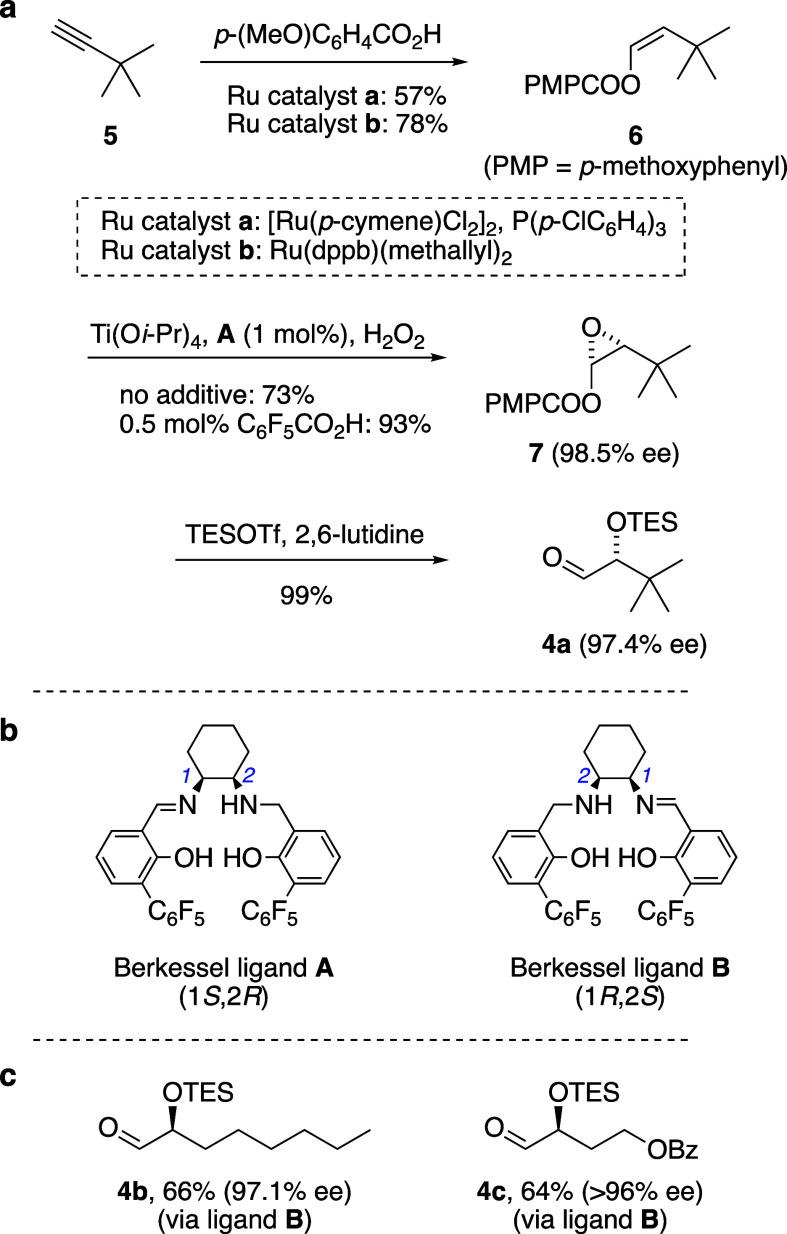
Three-Step Enantioselective Synthesis of α-Silyloxyaldehydes via Enol Ester Epoxides

Having reliable access to the enol ester epoxide, we next assessed retention of the configuration in the silyl cation-mediated epoxide ring-opening reaction. Upon exposure to triethylsilyl triflate in the presence of 2,6-lutidine, the enol ester epoxide was cleanly and rapidly transformed to α-silyloxyaldehyde **4a** in quantitative yield; the configuration had been retained with 97.4% ee. The overall yield of 72% and step economy of the sequence from alkyne to α-silyloxyaldehyde is noteworthy. Following the same sequence using enantiomeric Berkessel ligand **B**, 1-octyne and 3-butyn-1-yl benzoate were converted in three steps to the corresponding α-silyloxyaldehydes **4b** and **4c** (Scheme 3c). The step economy of this approach compares favorably with a seven-step route to **4c** from 2-deoxy-d-ribose.^5e^ Interestingly, despite the compatibility of the silyl ethers of **4a**–**4c** with the reaction conditions, the sequence did not offer access to a structure bearing a *tert*-butyldimethylsilyl ether in place of the benzoate of **4c**. Further investigation of scope and reactivity in this route to α-silyloxyaldehydes is underway and will be reported in due course.

With the enantiopure α-silyloxyaldehyde **4a** in hand, configuration-encoded 1,5-polyol synthesis via Julia–Kocienski coupling commenced (Scheme 4). From **4a**, three iterations of 1,5-diol assembly were applied utilizing the configuration-encoded γ-sulfononitrile building block (*S*)-**3**. The first coupling proceeded quantitatively, affording diol nitrile **8**; reduction of the nitrile was then required to reveal the aldehyde for the next coupling event. In our prior work to develop this sequence, we have observed that DIBAL-H reduction of α-silyloxynitriles can be accompanied by an epimerization at the α-silyloxyaldehyde α-carbon,^7b^ suspected to occur by imine-enamine tautomerization of the intermediate *N*-Al or *N*-H imines during hydrolytic workup. Reduction of diol nitrile **8** was such a case, with occasional leakage of diastereomeric purity prompting us to address this problem with a more general and practical workup protocol: After any remaining aluminum hydride was quenched with methanol at ca. – 20 °C, the mixture was stirred with equal volumes of Et_2_O and aqueous dibasic phosphate buffer (K_2_HPO_4_, 4.5 M) for 1 h, leading to an easily separated organic phase containing the intermediate *N*-H imine, free of aluminum salts. The ether solution of the imine was then stirred with wet Dowex acidic ion-exchange resin for several hours to complete the hydrolysis, affording α-silyloxyaldehyde **9** in 72% yield without detectable epimerization. Using this modified hydrolytic workup, the same two-step sequence of Julia–Kocienski coupling and DIBAL-H reduction was repeated twice more, again without detectable epimerization, to furnish triol aldehyde **11** and then tetraol aldehyde **13**.

**Scheme 4:**
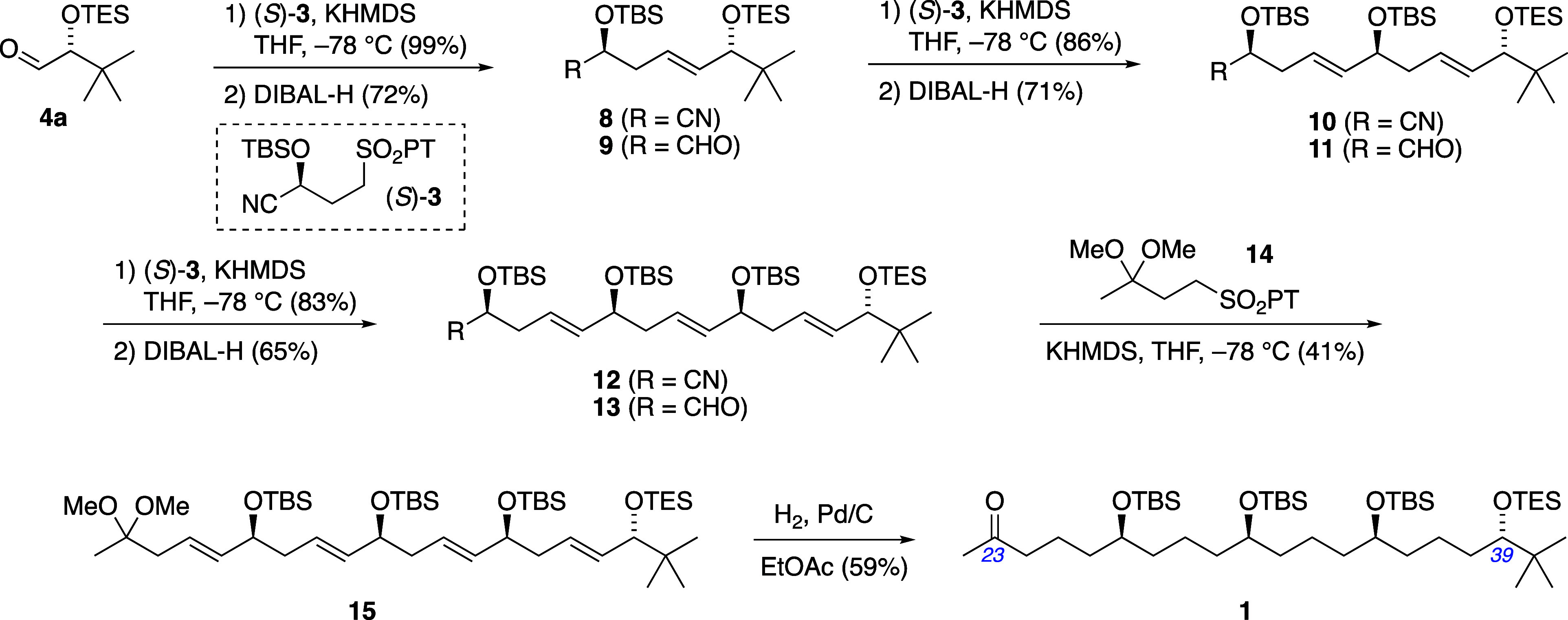
Configuration-Encoded Assembly of the C22–C41 Fragment of Bastimolides A and B

Lastly, the assembled 1,5-polyol was readied for the projected Mukaiyama aldol fragment coupling (Scheme 4). A final Julia–Kocienski olefination of aldehyde **13** with sulfone **14**, prepared in three steps from methyl vinyl ketone (see Supporting Information),^18^ afforded dimethyl ketal **15**. The alkenes were then saturated under typical hydrogenation conditions (1 atm of H_2_, 10% Pd/C, EtOAc) which also led to ketal hydrolysis, furnishing **1**, the southern 1,5-polyol fragment of bastimolides. Compound **1** contains the C23 ketone functionality needed for aldol coupling to the northern fragment as well as functional group differentiation suitable for selective esterification of alcohols at either C39 (for bastimolide A) or C23 (for bastimolide B).

In conclusion, we have (a) introduced an asymmetric modification of our 3-step route from hydrocarbons to α-silyloxyaldehydes, (b) improved the reliability of DIBAL-H reduction of enantiopure α-silyloxynitriles, and (c) applied these findings to address stereochemical challenges in 1,5-polyol synthesis, culminating in an asymmetric synthesis of the C22–C41 fragment of the bastimolides. The strategy of iterative coupling of configuration-encoded building blocks offers unambiguous control of remote stereogenic centers in such 1,5-polyol targets.

## Data Availability

The data underlying this study are available in the published article and its Supporting Information.

## Supporting Information Available

Preparative procedures characterization data including spectra for all new compounds (PDF)
