# Supplementary material for: A Three-Step Catalytic Asymmetric Sequence from Alkynes to α-Silyloxyaldehydes and Its Application to a C22–C41 Fragment of Bastimolide A
Source: Org Lett. 2024 May 16;26(21):4492–6. doi: 10.1021/acs.orglett.4c01310 (PMC11148846; doi:10.1021/acs.orglett.4c01310)
Supplement: Supplementary file 1 — ol4c01310_si_001.pdf [file ol4c01310_si_001.pdf]

A Three-Step Catalytic Asymmetric Sequence from Alkynes to α-Silyloxyaldehydes and Its Application to a C22–C41 Fragment of Bastimolide A

Jacob N. Hackbarth and Gregory K. Friestad*

Department of Chemistry, University of Iowa, Iowa City, IA 52242, USA

*Corresponding Author: gregory-friestad@uiowa.edu

Supporting Information

Table of Contents

Materials and Methods S2

Preparative Procedures S3

References S19

Spectral Data for New Compounds S20

Materials and Methods

Reactions employed oven- or flame-dried glassware under nitrogen unless otherwise noted. THF, diethyl ether, CH_2Cl_2, benzene and toluene were purchased inhibitor-free, sparged with argon, and passed through columns of activated alumina prior to use (dropwise addition of blue benzophenone ketyl solution revealed the THF purified in this manner sustained the blue color more readily than the control sample purified by distillation). Nitrogen was passed successively through columns of anhydrous $CaSO_4$ and R3-11 catalyst for removal of water and oxygen, respectively. All other materials were used as received from commercial sources unless otherwise noted. Thin layer chromatography (TLC) employed glass 0.25 mm silica gel plates with UV indicator. Flash chromatography columns were packed with 230–400 mesh silica gel as a slurry in the initial elution solvent. Gradient flash chromatography was conducted by adsorption of product mixtures on silica gel, packing over a short pad of clean silica gel as a slurry in hexane, and eluting with a continuous gradient from hexane to the indicated solvent. Radial chromatography refers to centrifugally accelerated thin-layer chromatography performed with a Chromatotron using commercially supplied rotors. Melting points are uncorrected. Nuclear magnetic resonance (NMR) data were obtained at operating frequencies of 600, 500, 400, or 300 MHz for ^{1}H and 150, 125, 100 or 75 MHz for ^{13}C, respectively. Optical rotations were determined using a digital polarimeter operating at ambient temperature. High-resolution mass spectra were obtained using a Thermo Q Exactive hybrid quadrupole Orbitrap mass spectrometer. Chromatographic stereoisomer ratio analyses employed HPLC using Chiralcel OD-H, AD-H, or OJ-3 columns with 2-propanol/hexane as mobile phase and photodiode array UV detection. Microwave synthesizer experiments employed a CEM Discover 2 with sealed tubes; power setting is automated to maintain the continuously monitored pressure and temperature within limits as indicated in the experimental procedures.

Preparative Procedures

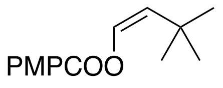

6
(PMP = *p*-methoxyphenyl)

(*Z*)-3,3-Dimethylbut-1-en-1-yl 4-methoxybenzoate (6). In a modification of the Goossen procedure,[1] a mixture of [(*p*-cymene)$RuCl_2$]$_2$ (1.0062 g, 1.64 mmol, 0.05 equiv), $P(C_6H_4Cl)_3$ (1.80 g, 4.92 mmol, 0.15 equiv), DMAP (0.8029 g, 6.57 mmol, 0.20 equiv), and *p*-anisic acid (5.000 g, 32.86 mmol) was prepared in deoxygenated 1,2-dichloroethane (DCE, 700 mL). Argon was bubbled through the mixture via syringe needle for 15 min with vigorous stirring. After stirring another 30 min, 3,3-dimethyl-1-butyne (7.6 mL, 61.7 mmol, 1.9 equiv) was added, and the flask was heated in an oilbath at 80 °C for 12 h. The mixture was cooled to 0 °C and an oxidative workup was applied to assist in removal of ruthenium byproducts:[2] aqueous 30% H_2O_2 (5–10 equiv, diluted with an equal volume of water) was added (Caution: O_2 may be produced; allow sufficient headspace to accomodate foaming), and the mixture was stirred for 1 h. The mixture was extracted with CH_2Cl_2 and filtered through silica gel, eluting with CH_2Cl_2. Concentration and flash chromatography (SiO_2, 50:1 hexanes/EtOAc) furnished **6**[3] (4.3935 g, 57% yield, *Z/E* >98:2) as a waxy yellow semi solid.[i,ii]

Alternative procedure: In a modification of Dixneuf's method,[4] to a Schlenk tube under Ar was added *p*-anisic acid (456 mg, 3.00 mmol) and Ru(dppb)(methallyl)$_2$[5] (19 mg, 0.03 mmol, 1 mol%). The tube was evacuated and refilled with Ar, then freshly distilled 1,2-dichloroethane (1.5 mL) and 3,3-dimethyl-1-butyne (0.74 mL, 6.0 mmol) were added via syringe through a

---

[i] Ruthenium byproducts interfere with the subsequent epoxidation reaction. Removal of ruthenium byproducts was judged successful if there was an absence of orange, red, or black color.

[ii] For addition of *p*-anisic acid to 3,3-dimethyl-1-butyne (*tert*-butylacetylene), this procedure offered higher yield than the General Procedure A (57% vs 40%).

rubber septum. The reaction mixture was heated using an oilbath at 55–60 °C, with pressure equalizing through an open Ar inlet, and after 5 min, the rubber septum was replaced with a greased ground-glass stopper and heating was continued with the Ar inlet closed. After 2 d at 55–60 °C, the cooled reaction mixture was partitioned between saturated aqueous $NaHCO_3$ (10 mL) and CH_2Cl_2 (3 x 10 mL), dried (Na_2SO_4), and concentrated in vacuo to an orange oil. Flash chromatography with gradient elution (5% to 10% hexanes/Et_2O) gave **6** (551 mg, 78% yield, *Z/E* >98:2) as a colorless oil.

General Procedure A: Ru-Catalyzed Addition of *p*-Anisic Acid to Alkynes

A mixture of [(*p*-cymene)$RuCl_2$]$_2$ (0.05 equiv), $P(C_6H_4Cl)_3$ (0.15 equiv), and DMAP (0.20 equiv) in 1,2-DCE[iii] (0.0036 M with respect to [(*p*-cymene)$RuCl_2$]$_2$) was stirred for 45 min under argon at 75 °C. This mixture was added via cannula into a suspension of *p*-anisic acid (1.0 equiv) and alkyne (1.4 equiv) in 1,2-DCE (0.167 M with respect to *p*-anisic acid), and stirring was continued at 75 °C for 12 h.[iv] The crude reaction mixture was filtered through silica gel, eluting with CH_2Cl_2, then concentrated in vacuo and purified via flash chromatography (SiO_2, 50:1 hexanes/EtOAc). If ruthenium byproducts remained[v] the product was dissolved in EtOAc at 70 °C, and 20 mg aliquots of ruthenium scavenger Snatch-Cat[6] were added every 20 min until there was no color change. Then the sample was filtered through silica gel, eluting with EtOAc. Concentration in vacuo furnished the (*Z*)-enol ester.

[iii] A mixture of 1,2-dichloroethane (1,2-DCE) and 3Å molecular sieves was sonicated under vacuum for ca. 30 s, then backfilled with argon, and this procedure was repeated 6 times. Without deoxygenating the solvent, inconsistent yields were observed, and phosphine oxide was detected after the reaction.

[iv] For addition of *p*-anisic acid to 1-octyne, yields with varied reaction temperatures were as follows: 50 °C, 25% yield; 60 °C, 55% yield; 70 °C, 78% yield (in triplicate).

[v] Presence of ruthenium byproducts (observable as orange, red, or black color) interferes with the subsequent epoxidation reaction.

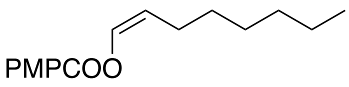

S1b
(PMP = *p*-methoxyphenyl)

(*Z*)-Oct-1-en-1-yl 4-methoxybenzoate (S1b). From oct-1-yne (0.15 mL, 1.0 mmol, 1.4 equiv) and *p*-anisic acid (0.1079 g, 0.709 mmol) via General Procedure A was obtained known compound **S1b**[7] (0.1459 g, 79% yield, *Z/E* >98:2) as a colorless oil.

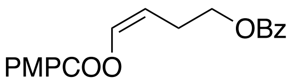

S1c
(PMP = *p*-methoxyphenyl)

(*Z*)-4-(Benzoyloxy)but-1-en-1-yl 4-methoxybenzoate (S1c). From but-3-yn-1-yl benzoate[8] (1.8 g, 10.3 mmol, 1.4 equiv) and *p*-anisic acid (1.12 g, 7.38 mmol) via General Procedure A was obtained enol ester **S1c** (1.7726 g, 74% yield, *Z/E* >98:2) as a colorless solid. mp 69–72 °C; ^{1}H NMR (500 MHz, CDCl$_3$): δ 8.05 (d, *J* = 8.99 Hz, 2H), 8.04-8.02 (m, 2H), 7.54-7.51 (m, 1H), 7.41-7.38 (m, 3H), 6.95 (d, J= 8.99 Hz, 2H), 5.08 (dt, apparent q, *J* = 7.06 Hz, 1H), 4.43 (t, *J* = 6.67 Hz, 2H), 3.88 (s, 3H), 2.79-2.75 (m, 2H); ^{13}C NMR {^{1}H} (125 MHz, CDCl$_3$): δ 166.5, 163.9, 163.0, 136.3, 132.9, 132.0, 130.2, 129.5, 128.3, 121.3, 113.8, 109.0, 63.8, 55.4, 24.6; HRMS (FTMS + p ESI) *m/z*: [M+Na]$^+$ Calcd for $C_{19}H_{18}O_5Na$ 349.1046; Found 349.1043.

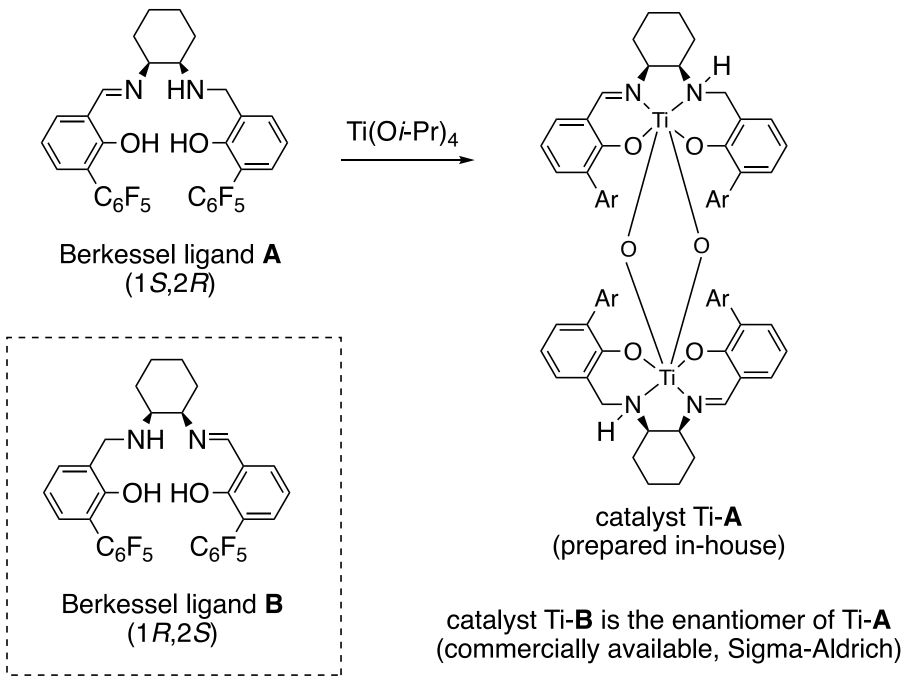

General Procedure B: Berkessel–Katsuki Epoxidation. To a screw cap vial equipped with micro stir bar were added enol ester, Berkessel–Katsuki epoxidation catalyst[9] Ti-**A** or Ti-**B** (0.002 equiv), 1,2-DCE (6 M in enol ester), and 30% aqueous H_2O_2 (2.5 equiv). The mixture was vigorously stirred for 48 h at room temperature with the cap loosely attached to permit pressure release.[vi] Then the reaction was diluted with CH_2Cl_2 and tested for peroxides with a water-wetted test strip; if positive, then the reaction was quenched with saturated aqueous thiosulfate solution. The organic phase was dried over Na_2SO_4 and concentrated in vacuo. Flash chromatography (SiO_2, 10:1 hexanes/EtOAc) furnished the enol ester epoxide.

[vi] Oxygen evolution may occur, and can be more significant if ruthenium impurities are carried forward from the previous step.

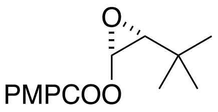

7
(PMP = *p*-methoxyphenyl)

(2*S*,3*R*)-3-(*tert*-Butyl)oxiran-2-yl 4-methoxybenzoate (7). From enol ester **6** (4.39 g, 18.7 mmol) and Ti-**A** (0.045 g, 0.030 mmol, 0.32 mol%) via General Procedure B was obtained known epoxide **7**[3] (3.434 g, 73% yield, 98.59% ee by HPLC) as a colorless solid. mp 56–58 °C; $[\alpha]_D^{23}$ –27.2 (*c* 12.98, $CHCl_3$); HPLC (Chiralcel OD-H, gradient from 0.3% to 40% IPA in hexanes over 40 min, flow rate = 1.00 mL/min, *l* = 254 nm) t_R = 9.212 min (major), 8.668 min (minor).

In another run, the additive $C_6F_5CO_2H$ (0.5 mol%) was employed, as described by Berkessel.[10] From enol ester **6** (0.898 g, 3.83 mmol), Ti-**A** (11 mg, 0.0074 mmol, 0.2 mol%), and $C_6F_5CO_2H$ (4 mg, 0.02 mmol, 0.5 mol%) was obtained epoxide **7** (0.894 g, 93% yield). $[\alpha]_D^{23}$ –30.6 (*c* 3.47, $CHCl_3$).

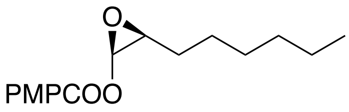

S2b
(PMP = *p*-methoxyphenyl)

(2*R*,3*S*)-3-Hexyloxiran-2-yl 4-methoxybenzoate (S2b). From enol ester **S1b** (0.1118 g, 0.4261 mmol, 1.0 equiv) and Ti-**B** (0.0035 g, 0.0023 mmol) via General Procedure B was obtained known compound **S2b**[3] (0.0749 g, 63% yield, 97.84% ee by HPLC) as a colorless waxy semi-solid. $[\alpha]_D^{23}$ +27.1 (*c* 5.39, $CHCl_3$); HPLC (Chiralcel OD-H, gradient from 0.3% to 40% IPA in hexanes over 40 min, flow rate = 1.00 mL/min, *l* = 254 nm) t_R = 9.74 min (major), 9.36 min (minor).

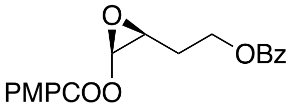

S2c
(PMP = *p*-methoxyphenyl)

(2*R*,3*S*)-3-(2-(Benzoyloxy)ethyl)oxiran-2-yl 4-methoxybenzoate (S2c). From enol ester **S1c** (0.5187 g, 1.52 mmol) and Ti-**B** (0.0046 g, 0.031 mmol) via General Procedure B was obtained compound **S2c** (0.4614 g, 84% yield, 97.6% ee by HPLC) as a pale yellow solid. mp 76–78 °C; $[\alpha]_D^{23}$ +51.4 (*c* 7.19, $CHCl_3$); HPLC (Chiralcel OD-H, gradient from 0.3% to 40% IPA in hexanes over 40 min, flow rate = 1.00 mL/min, *l* = 258 nm) t_R = 23.056 min (major), 21.484 min (minor); ^{1}H NMR (400 MHz, $CDCl_3$): δ 8.03 (d, *J* = 8.48 Hz, 2H), 7.97 (d, *J* = 8.99 Hz, 2H), 7.56-7.52 (m, 1H), 7.42-7.38 (m, 2H), 6.92 (d, *J* = 8.99 Hz, 2H), 5.84 (d, *J* = 2.53 Hz, 1H), 4.63-4.52 (m, 2H), 3.87 (s, 3H), 3.32 (ddd, J_1 = 6.51 Hz, J_2 = 5.69 Hz, J_3 = 2.56 Hz, 1H), 2.35-2.21 (m, 2H); ^{13}C NMR {^{1}H} (100 MHz, $CDCl_3$): δ 166.5, 165.8, 164.2, 133.2, 132.1, 130.0, 129.7, 128.5, 121.2, 113.9, 75.8, 61.9, 55.6, 54.1, 27.3; HRMS (FTMS + p ESI) *m/z*: $[M+Na]^+$ Calcd for $C_{19}H_{18}O_6Na$ 365.0996; Found 365.0990.

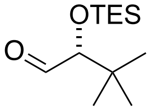

4a

(*R*)-3,3-Dimethyl-2-((triethylsilyl)oxy)butanal (4a). To a solution of epoxide **7** (1.1190 g, 4.47 mmol) in CH_2Cl_2 (18 mL) were added 2,6-lutidine (1.04 mL, 8.94 mmol, 2 equiv) and freshly distilled triethylsilyl trifluoromethanesulfonate (1.11 mL, 6.12 mmol, 1.1 equiv). After 20 min, the reaction mixture was partitioned between saturated aqueous ammonium chloride (5 mL) and CH_2Cl_2 (15 mL). Concentration and flash chromatography (50:1 hexanes/EtOAc) yielded α-silyloxyaldehyde **4a** (1.0181 g, 99% yield, 97.4% ee) as a colorless oil. Caution: This compound is easily evaporated; vacuum must be applied with care to avoid material loss. $[\alpha]_D^{21}$ +15.4 (*c*

2.38, $CHCl_3$); ^1H NMR (400 MHz, $CDCl_3$): δ 9.60 (d, $J = 3.3$Hz, 1H), 3.49 (d, $J = 3.3$ Hz, 1H), 0.97-0.93 (m, 18H), 0.60 (q, $J = 7.94$ Hz, 6H); ^{13}C NMR {^1H} (100 MHz, $CDCl_3$): δ 204.9, 84.4, 35.9, 25.8, 6.8, 4.9; HRMS (FTMS + p ESI) *m/z*: $[M+Na]^+$ Calcd for $C_{12}H_{26}O_2SiNa$ 253.1594; Found 253.1589.

Reduction and desilylation of **4a** provided known 3,3-dimethylbutane-1,2-diol; the sample was levorotatory, establishing the absolute configuration of **4a** as (*R*).[11] Benzoylation provided a monobenzoate derivative for which enantiopurity of 97.41% ee was determined via HPLC (Chiralcel OJ-3, gradient from 0.3% to 40% IPA in hexanes over 40 min, hold at 40% for 20 min, flow rate = 1.00 mL/min, *l* = 229 nm) t_R = 25.148 min (major), 25.968 min (minor).

General Procedure C: **Silyl Cation-Induced Epoxide Opening (microwave).**[vii] To a solution of epoxide (1 equiv) in CH_2Cl_2 (0.25 M) in a microwave reactor tube were added 2,6-lutidine (2 equiv) and freshly distilled triethylsilyl trifluoromethanesulfonate (TESOTf, 1.1 equiv) under argon atmosphere. The tube was sealed and subjected to microwave irradiation (100 W) for 30 min with temperature and pressure limits set to 50 °C and 60 psi. The reaction mixture was quenched with aqueous saturated ammonium chloride solution and extracted with CH_2Cl_2 (15 mL) Concentration and flash chromatography (petroleum ether/EtOAc) afforded the α-silyloxyaldehyde.

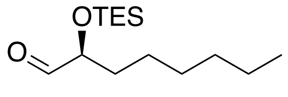

4b

(*S*)-2-((Triethylsilyl)oxy)octanal (4b). From enol ester epoxide **S2b** (0.1050 g, 0.377 mmol, 1.0 equiv), 2,6-lutidine (0.109 mL, 0.94 mmol, 2.5 equiv), and TESOTf (0.076 g, 0.399 mmol, 1.1

[vii] In some early experiments, heating was applied via microwave to minimize reaction time. Microwave heating is optional.

equiv) via General Procedure C, was obtained α-silyloxyaldehyde **4b** (0.0619 g, 66% yield, 97.1% ee) as a colorless oil. $[\alpha]_D^{21}$ –24.6 (*c* 2.0, $CHCl_3$); ^{1}H NMR (400 MHz, $CDCl_3$): δ 9.59 (d, *J* = 1.87 Hz, 1H), 3.96-3.93 (m, 1H), 1.64-1.25 (m, 12H), 0.99-0.93 (t, *J* = 7.91 Hz, 9H), 0.88 (t, *J* = 6.82 Hz, 3H), 0.65-0.59 (m, 6H); ^{13}C NMR {^{1}H} (100 MHz, $CDCl_3$): δ 204.5, 77.6, 32.9, 31.7, 29.3, 24.6, 22.6, 14.1, 6.8, 4.9; HRMS (FTMS + p ESI) *m*/*z*: $[M+Na]^+$ Calcd for $C_{14}H_{30}O_2SiNa$ 281.1907; Found 281.1898.

Reduction, desilylation, and benzoylation of **4b** provided the known 1,2-octanediol dibenzoate derivative for which (*S*)-configuration and enantiopurity of 97.1% ee were determined by polarimetry and HPLC respectively. $[\alpha]_D^{20}$ –7.0 (*c* 0.26, $CHCl_3$), lit.[12] $[\alpha]_D^{21}$ –6 (*c* 0.15, $CDCl_3$); HPLC (Chiralcel OJ-3, gradient from 0.3% to 40% IPA in hexanes over 40 min, hold at 40% for 20 min, flow rate = 1.00 mL/min, *l* = 234 nm) t_R = 7.072 min (major), 6.828 min (minor).

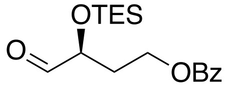

4c

(*S*)-4-Oxo-3-((triethylsilyl)oxy)butyl benzoate (4c). From enol ester epoxide **S2b** (0.1013 g, 0.292 mmol, 1.0 equiv), 2,6-lutidine (0.067 mL, 0.584 mmol, 2 equiv), and TESOTf (0.061g, 0.31 mmol, 1.1 equiv) via General Procedure C, was obtained α-silyloxyaldehyde **4c** (0.0613 g, 64% yield) as a colorless oil. $[\alpha]_D^{23}$ –12.9 (*c* 3.17, $CHCl_3$); ^{1}H NMR (400 MHz, $CDCl_3$): δ 9.69 (d, *J* = 1.32 Hz, 1H), 8.01-7.98 (m, 2H), 7.58-7.54 (m, 1 H), 7.46-7.42 (m, 2H), 4.53-4.37 (m, 2H), 4.23-4.20 (m, 1H), 2.19-2.04 (m, 2H), 0.96 (t, *J* = 7.92 Hz, 9H), 0.64 (q, *J* = 7.92 Hz, 6H); ^{13}C NMR {^{1}H} (100 MHz, $CDCl_3$): δ 203.9, 166.3, 133.1, 130.0, 129.6, 128.5, 74.5, 60.2, 32.3, 6.7, 4.7; HRMS (FTMS + p ESI) *m*/*z*: $[M+Na]^+$ Calcd for $C_{17}H_{26}O_4NaSi$ 345.1493; Found 345.1486.

Reduction ($NaBH_4$) and esterification with (*S*)-(+)-MTPA-Cl afforded the corresponding (*R*)-MTPA ester.[13] Only one diastereomer was detected by 500 MHz ^{1}H NMR (e.r. >98:2, >96% ee).

General Procedure D: Julia-Kocienski Olefination To a solution of sulfone (2–6 equiv relative to aldehyde) in THF (0.045 M) was added KHMDS (0.5 M in toluene, 0.95 equiv relative to sulfone) at –78 °C. After 45 min, the requisite aldehyde in THF (0.2 M) was added dropwise to the reaction mixture at –78 °C. After 3–12 hr at –78 °C,[viii] the reaction mixture was quenched with aqueous NH_4Cl solution and allowed to warm to room temperature, then extracted with EtOAc. The organic phase was washed with water and brine, then dried over Na_2SO_4. Concentration and flash chromatography (SiO_2, hexanes/EtOAc) afforded the alkene products.

General Procedure E: DIBAL Reduction and Hydrolysis. To a solution of α-silyloxynitrile (1.0 equiv) in toluene (0.7 M) at –78 °C was added DIBAL-H (1.0 M in toluene, 1.5 equiv) dropwise. The mixture was allowed to warm to –20 °C over the course of 5 h, then held at –20 °C for 2 h. The reaction mixture was quenched at –20 °C by addition of MeOH (1.5 mL per mmol of starting nitrile), then aqueous K_2HPO_4 (4.5 M, 5 mL per mmol starting nitrile) and an equal volume of Et_2O were added, and the biphasic mixture was allowed stir at 20 °C for 1 h, resulting in a clean phase separation. After separation of the organic phase, the aqueous phase was extracted three times with Et_2O, and the combined organic phases were dried over Na_2SO_4, and concentrated in vacuo to give crude imine.[ix] To a solution of the imine in Et_2O/H_2O (10:1 ratio, 30 mL per mmol of starting nitrile) was added Dowex 50wx8 200-mesh acidic ion-exchange resin[x] (0.5 g per mmol of starting nitrile). The reaction was stirred for 12 h, or monitored by NMR every hour until there was no imine peak present. After filtration, the organic phase was dried

[viii] In some cases, the reaction was allowed to warm to ca. 20 °C prior to quenching, with no apparent detriment.

[ix] The crude imine was usually accompanied by varying amounts of aldehyde, as judged by ^{1}HNMR (imine: δ 7.5–8.1 ppm, aldehyde: δ 9.6 ppm); epimerization at the α-carbon may be detected by the presence of additional peaks in the same vicinities.

[x] Prior to use, the resin was treated with 0.5 M aqueous sulfuric acid in a sintered glass funnel, followed by washings with water, MeOH and then Et_2O. Resin treated in this way could be used for several months without any apparent change in reactivity. Active resin, on mixing with brine solution, causes the solution to test acidic with pH paper.

(Na_2SO_4) and concentrated in vacuo. The residue was taken up in a small amount of Et_2O and filtered through a short pad of silica gel, eluting with Et_2O. After concentration in vacuo, the aldehyde was obtained in sufficient purity for the next step. Further chromatography should be minimized to avoid material losses and/or epimerization.

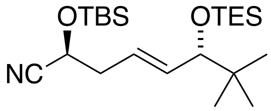

8

(2*S*,6*S*,*E*)-2-((*tert*-Butyldimethylsilyl)oxy)-7,7-dimethyl-6-((triethylsilyl)oxy)oct-4-enenitrile (8). From sulfone (*S*)-**3** (0.9816 g, 2.37 mmol) and α-silyloxyaldehyde **4a** (0.2775 g, 1.2 mmol) in 12.6 mL THF via General Procedure D was obtained **8** (0.4972 g, 99% yield, *E*/*Z* 91:9) as a colorless oil. $[\alpha]_D^{23}$ –13.4 (*c* 2.59, $CHCl_3$); ^1H NMR (500 MHz, $CDCl_3$): δ 5.69-5.51 (m, 2H), 4.43 (t, J = 6.13 Hz, 1H), 3.69 (d, J = 6.82, 1H), 2.58-2.45 (m, 2H), 0.96 (t, J = 8.03 Hz, 9H), 0.92 (s, 9H), 0.85 (s, 9H), 0.57 (m, 6H), 0.19 (s, 3H), 0.15 (s, 3H); ^{13}C NMR {^1H} (100 MHz, $CDCl_3$): δ 136.7, 123.6, 119.8, 81.2, 62.3, 39.5, 35.7, 26.0, 25.6, 18.2, 7.1, 5.2, –5.0, –5.2; HRMS (FTMS + p ESI) *m*/*z*: $[M+Na]^+$ Calcd for $C_{22}H_{45}NNaO_2Si_2$ 434.2881; Found 434.2883.

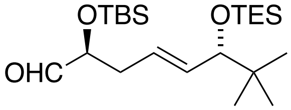

9

(2*S*,6*S*, E)-2-((*tert*-Butyldimethylsilyl)oxy)-7,7-dimethyl-6-((triethylsilyl)oxy)oct-4-enal (9). From α-silyloxynitrile **8** (*E*/*Z* 91:9, 0.195 g, 0.4735 mmol) and DIBAL-H (1.0 M in toluene, 0.71 mL, 0.71 mmol) via General Procedure E was obtained α-silyloxyaldehyde **9** (0.1414 g, 72% yield, *E*/*Z* 91:9) as an orange oil. $[\alpha]_D^{23}$ –6.3 (*c* 1.1, $CHCl_3$); ^1H NMR (400 MHz, $CDCl_3$): δ 9.59 (d, J = 1.95 Hz, 1H), 5.54-5.52 (m, 2H), 4.04-4.00 (m, 1H), 3.64-3.62 (m, 1H), 2.43-2.40 (m, 2H),

0.95-0.91 (m, 18H), 0.82 (s, 9H), 0.58-0.52 (m, 6H), 0.09 (s, 3H), 0.08 (s, 3H); ^{13}C NMR {^{1}H} (100 MHz, $CDCl_3$): δ 204.2, 134.9, 125.3, 81.4, 77.6, 36.3, 35.7, 26.0, 25.9, 18.3, 7.1, 5.2, –4.5, –4.8; HRMS (FTMS + p ESI) *m*/*z*: $[M+Na]^+$ Calcd for $C_{22}H_{46}O_3NaSi_2$ 437.2878 ; Found 437.2873.

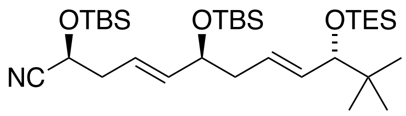

10

(2*S*,4*E*,6*S*,8*E*,10*S*)-2,6-Bis((*tert*-butyldimethylsilyl)oxy)-11,11-dimethyl-10-((triethylsilyl)oxy)dodeca-4,8-dienenitrile (10). From sulfone (*S*)-**3** (0.5971 g, 1.47 mmol) and α-silyloxyaldehyde **9** (0.2438 g, 0.5878 mmol) via General Procedure D was obtained **10** (0.2999 g, 85.6 % yield) as a colorless oil. $[\alpha]_D^{22}$ –2.83 (*c* 1.09, $CHCl_3$); ^{1}H NMR (500 MHz, $CDCl_3$): δ 5.68-5.41 (m, 4H), 4.41 (t, *J* = 6.20 Hz, 1H), 4.14 (q, *J* = 5.43 Hz, 1H), 6.63 (d, *J* = 6.82 Hz, 1H), 2.53-2.45 (m, 2H), 2.29-2.19 (m, 2H), 0.93 (t, *J* = 7.89 Hz, 9H), 0.91 (s, 9H), 0.90 (s, 9H), 0.82 (s, 9H), 0.58-0.53 (m, 6H), 0.18 (s, 3H), 0.14 (s, 3H), 0.06 (s, 3H), 0.05 (s, 3H); ^{13}C NMR {^{1}H} (100 MHz, $CDCl_3$): δ 138.9, 133.6, 127.5, 122.1, 119.7, 81.7, 73.1, 62.2, 41.7, 39.4, 35.7, 26.1, 26.0, 25.7, 18.4, 18.2, 7.1, 5.3, –4.2, –4.7, –5.0, –5.2; HRMS (FTMS + p ESI) *m*/*z*: $[M+Na]^+$ Calcd for $C_{32}H_{65}O_3NNaSi_3$ 618.4164; Found 618.4160.

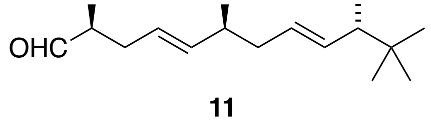

11

(2*S*,4*E*,6*S*,8*E*,10*S*)-2,6-Bis((*tert*-butyldimethylsilyl)oxy)-11,11-dimethyl-10-((triethylsilyl)oxy)dodeca-4,8-dienal (11). From α-silyloxynitrile **10** (0.119 g, 0.200 mmol) and DIBAL-H (1.0 M in toluene, 0.30 mL, 0.30 mmol) via General Procedure E was obtained α-

silyloxyaldehyde **11** (0.085 g, 71% yield) as a pale yellow oil. $[\alpha]_D^{22}$ –1.43 (*c* 1.50, $CHCl_3$); ^1H NMR (400 MHz, $CDCl_3$): δ 9.59 (d, *J* = 1.6 Hz, 1H), 5.63-5.34 (m, 4H), 4.09-4.05 (m, 1H), 4.01-3.95 (m, 1H), 3.61 (d, *J* = 6.56 Hz, 1H), 2.43-2.37 (m, 2H), 2.27-2.14 (m, 2 H), 0.94 (s, 9H), 0.58-0.53 (m, 6H), 0.18 (s, 3H), 0.14 (s, 3H), 0.06 (s, 3H), 0.04 (s, 3H); ^{13}C NMR {^1H} (100 MHz, $CDCl_3$): δ 204.0, 137.1, 133.4, 127.8, 123.9, 81.8, 77.4, 73.4, 41.8, 36.2, 35.7, 26.1, 26.0, 25.9, 18.3 (2C), 7.1, 5.3, –4.2, –4.6, –4.6, –4.7; HRMS (FTMS + p ESI) *m/z*: $[M+Na]^+$ Calcd for $C_{32}H_{66}O_4NaSi_3$ 621.4161; Found 621.4169.

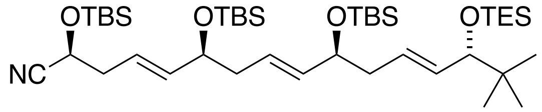

12

(2*S*,4*E*,6*S*,8*E*,10*S*,12*E*,14*S*)-2,6,10-Tris((*tert*-butyldimethylsilyl)oxy)-15,15-dimethyl-14-((triethylsilyl)oxy)hexadeca-4,8,12-trienenitrile (12). From sulfone (*S*)-**3** (0.173 g, 0.426 mmol) and α-silyloxyaldehyde **11** (0.085 g, 0.142 mmol) via General Procedure D was obtained **12** (0.092 g, 83% yield) as a colorless oil. $[\alpha]_D^{24}$ +1.87 (*c* 0.97, $CHCl_3$); ^1H NMR (400 MHz, $CDCl_3$): δ 5.68-5.39 (m, 6H), 4.41 (t, *J* = 3.37 Hz, 1H), 4.15 (m, 2H), 3.61 (d, *J* = 6.98 Hz, 1H), 2.53-2.45 (m, 2H), 2.28-2.15 (m, 4H), 0.95-0.88 (m, 32H), 0.82 (s, 9H), 0.58-0.52 (m, 6H), 0.18 (s, 3H), 0.14 (s, 3H), 0.05-0.02 (m, 9H), 0.00 (s, 3H); ^{13}C NMR {^1H} (100 MHz, $CDCl_3$): δ 138.8, 136.0, 133.2, 128.0, 126.1, 122.1, 119.6, 81.8, 73.7, 72.9, 62.2, 41.9, 41.4, 39.4, 35.7, 26.1, 26.1, 26.0, 25.7, 18.4 (2C), 18.2, 7.1, 5.3, –4.0, –4.3, –4.6, –4.6, –5.0, –5.2; HRMS (FTMS + p ESI) *m/z*: $[M+Na]^+$ Calcd for $C_{42}H_{85}O_4NNaSi_4$ 802.5448; Found 802.5454.

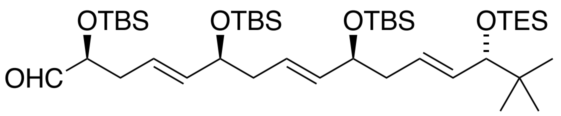

13

(2*S*,4*E*,6*S*,8*E*,10*S*,12*E*,14*S*)-2,6,10-Tris((*tert*-butyldimethylsilyl)oxy)-15,15-dimethyl-14-((triethylsilyl)oxy)hexadeca-4,8,12-trienal (13). From α-silyloxynitrile **12** (26.6 mg, 0.0341 mmol) and DIBAL-H (1.0 M in toluene, 0.06 mL, 0.06 mmol) via General Procedure E was obtained α-silyloxyaldehyde **13** (17.3 mg, 65% yield) as a pale yellow oil. $[\alpha]_D^{22}$ +3.01(*c* 1.04, $CHCl_3$); ^{1}H NMR (400 MHz, $CDCl_3$): δ 9.59 (d, *J* = 1.44, 1H), 5.57-5.39 (m, 6H), 4.08-3.99 (m, 2H), 3.61 (d, *J* = 7.05 Hz, 1H), 2.41-2.14 (m, 6H), 0.97-0.88 (m, 36H), 0.82 (s, 9H), 0.58-0.52 (m, 6H), 0.10-0.01 (m, 18H); ^{13}C NMR {^{1}H} (100 MHz, $CDCl_3$): δ 204.0, 137.0, 135.8, 133.1, 128.1, 126.4, 123.9, 81.8, 77.4, 73.8, 73.2, 41.9, 41.5, 36.2, 35.7, 26.1, 26.1, 26.0, 25.9, 18.37, 18.36, 18.3, 7.1, 5.3, –4.0, –4.2, –4.6, –4.6, –4.6, –4.7; HRMS (FTMS + p ESI) *m*/*z*: $[M+Na]^+$ Calcd for $C_{42}H_{86}O_5NaSi_4$ 805.5445; Found 805.5456.

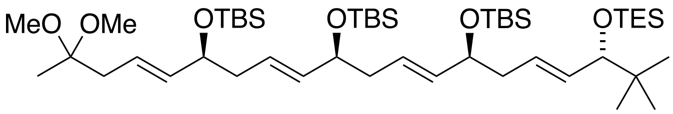

15

(5*S*,6*E*,9*S*,10*E*,13*S*,14*E*,17*S*)-5-(*tert*-Butyl)-9,13-bis((*tert*-butyldimethylsilyl)oxy)-17-((E)-4,4-dimethoxypent-1-en-1-yl)-3,3-diethyl-19,19,20,20-tetramethyl-4,18-dioxa-3,19-disilahenicosa-6,10,14-triene (15). From sulfone **14** (78.3 mg, 0.240 mmol) and α-silyloxyaldehyde **13** (54.4% w/w,[xi] 54.2 mg, 0.0377 mmol) via General Procedure D (except quenching with H_2O instead of aq. NH_4Cl) was obtained **15** (13.8 mg, 41.4 % yield) as a colorless oil. ^{1}H NMR (500 MHz, $CDCl_3$): δ 5.55-5.39 (m, 8H), 4.10-4.04 (m, 3H), 3.62 (d, *J* = 7.44, 1H), 3.19 (s, 6H), 2.38-2.30 (m, 2H), 2.27-2.13 (m, 6H), 1.24 (s, 3H), 0.93 (t, *J* = 7.94 Hz,

[xi] This sample contained 29.5 mg of the aldehyde (0.0377 mmol). The remainder of the mass was nitrile **12**.

9H), 0.89-0.88 (m, 27H), 0.83 (s, 9H), 0.58-0.53 (m, 6H), 0.04-0.02 (m, 18H); ^{13}C NMR {^{1}H} (100 MHz, $CDCl_3$): δ 136.5, 135.6, 135.5, 133.1, 128.1, 126.7, 126.4, 125.2, 101.4, 81.8, 73.9, 73.6, 73.4, 48.3, 42.0, 41.6, 41.6, 39.7, 35.7, 26.1, 26.0, 26.0, 22.9, 18.39 (2C), 18.37, 7.1, 5.3, –4.0, –4.1, –4.3, –4.6, –4.6, –4.6; HRMS (FTMS + p ESI) *m/z*: $[M+Na]^+$ Calcd for $C_{48}H_{98}O_6NaSi_4$ 905.6333; Found 905.6335.

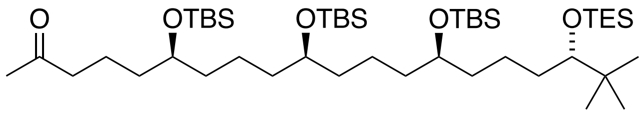

1

(6*S*,10*S*,14*R*,18*S*)-6,10,14 Tris((*tert*-butyldimethylsilyl)oxy)-18-(triethylsilyloxy)-19,19-dimethyldodecane-2-one (1). To a solution of ketal **15** (8.4 mg, 0.0095 mmol) in ethyl acetate (0.5 mL) was added Pd/C (10%, 1.4 mg, 0.0013 mmol Pd). The atmosphere was replaced with H_2 (5 cycles of brief evacuation and refilling from H_2 balloon) and the mixture was stirred for 2 d at 20 °C under H_2 (1 atm, balloon). The mixture was filtered through Celite and concentrated in vacuo to obtain ketone **1** (7.1 mg, 70% purity, 59% yield) as a colorless oil. This sample was a 2.34:1 mixture of **1** with a mono-olefin (HRMS (FTMS + p ESI) *m/z*: $[M+H]^+$ Calcd for $C_{46}H_{98}O_5Si_4$ 843.6564; Found 843.6553) that did not separate by flash chromatography with various solvent systems.[xii] The analytical sample of **1** was obtained by resubjecting a sample of the above mixture (3.4 mg) to the same hydrogenation conditions over 4 d, furnishing a pure sample of the fully saturated ketone **1** (1.9 mg) as a colorless oil. $[\alpha]_D^{20}$ –12 (*c* 0.27, $CHCl_3$); ^{1}H NMR (500 MHz, $CDCl_3$): δ 3.67–3.59 (m, 3H), 3.24 (br d, *J* = 7.2 Hz, 1H), 2.41 (m, apparent t, *J* = 7.2 Hz, 2H), 2.13 (s, 3H), 1.67–1.55 (m, 2H), 1.47–1.32 (m, 14H), 1.32–1.21 (m, 6H), 0.97 (t, *J* = 8.1 Hz, 9H), 0.89 (s, 9H), 0.88 (s, 18H), 0.85 (s, 9H), 0.61 (m, apparent q, *J* = 8.1 Hz, 6H),

[xii] Chromatography solvents attempted: 20:1 hexanes/EtOAc, 10% Et_2O in hexanes, or a 20:1 mixture of hexanes/(10% MeOH in CH_2Cl_2).

0.05–0.03 (m, 18H); ^{13}C NMR {^{1}H} (125 MHz, $CDCl_3$): δ 209.0, 81.3, 72.5, 72.3, 72.0, 44.0, 37.8, 37.41, 37.39, 37.3, 36.4, 35.7, 33.6, 29.8, 26.3, 26.0, 25.9, 23.6, 21.03, 20.98, 18.14, 18.11, 7.2, 5.7, –4.38, –4.44 (some peaks are not resolved); HRMS (FTMS + p ESI) *m/z*: $[M+Na]^+$ Calcd for $C_{46}H_{101}O_5Si_4$ 845.6721; Found 845.6706.

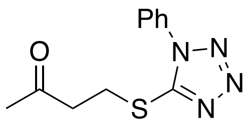

S3

4-((1-Phenyl-1H-tetrazol-5-yl)sulfanyl)butan-2-one (S3). To a suspension of 1-phenyl-1H-tetrazole-5-thiol (0.922 g, 5.17 mmol) in CH_2Cl_2 (2 mL) at 20 °C was added methyl vinyl ketone (freshly distilled, 0.60 mL, 7.2 mmol). The mixture became homogeneous after ca. 1 h. After 1 d, the solution was concentrated in vacuo to afford ketosulfide **S3** as a colorless oil which was sufficiently pure for the subsequent step. ^{1}H NMR (500 MHz, $CDCl_3$): δ 7.55-7.50 (m, 5H), 3.54 (t, J = 6.39 Hz, 2H), 3.12 (t, J = 6.39 Hz, 2H), 2.18 (s, 3H); ^{13}C NMR {^{1}H} (125 MHz, $CDCl_3$): δ 206.0, 154.4, 133.7, 130.3, 129.9, 123. 8, 43.0, 30.1, 26.8; HRMS (FTMS + p ESI) *m/z*: $[M+Na]^+$ Calcd for $C_{11}H_{13}N_4OS$ 249.0805; Found 249.0800.

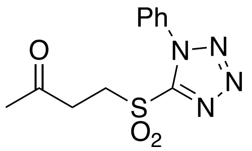

S4

4-((1-Phenyl-1H-tetrazol-5-yl)sulfonyl)butan-2-one (S4). To a solution of ketosulfide **S3** in 95% ethanol (50 mL) was added ammonium molybdate tetrahydrate (0.33 g, 0.27 mmol), and aqueous H_2O_2 (30%, 4.2 mL, 38 mmol), and the mixture was stirred at 20 °C. After 1 d, the mixture was concentrated to remove most of the ethanol, then diluted with H_2O (30 mL) to

complete precipitation of the solid product. Vacuum filtration, washing the filter cake with H_2O (2 x 10 mL), afforded ketosulfone **S4** (1.250 g, 86% yield) as a colorless solid. mp 81–83 °C; ^1H NMR (500 MHz, $CDCl_3$): δ 7.70-7.60 (m, 5H), 3.98 (t, J = 7.2 Hz, 2H), 3.19 (t, J = 7.2 Hz, 2H), 2.24 (s, 2H); ^{13}C NMR {^1H} (125 MHz, $CDCl_3$): δ 202.7, 153.5, 133.1, 131.7, 129.9, 125.3, 50.9, 36.5, 29.9; HRMS (FTMS + p ESI) *m/z*: $[M+H]^+$ Calcd for $C_{11}H_{13}N_4O_3S$ 281.0703; Found 281.0697.

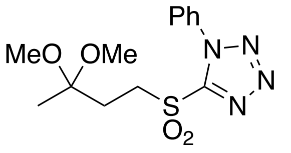

14

5-((3,3-Dimethoxybutyl)sulfonyl)-1-phenyl-1H-tetrazole (14). To a solution of ketosulfone **S4** (0.5211 g, 1.6 mmol) in trimethylorthoformate (2.3 mL, 18 mmol) was added *p*-TsOH·H_2O (0.091 g, 0.47 mmol, 0.3 equiv) and the resultant mixture was stirred for 1 h. This mixture was filtered through silica gel, eluting with 1:30 MeOH/CH_2Cl_2 to give ketalsulfone **14** (0.5918 g, 97% yield) as a colorless solid.[xiii] mp 76–79 °C; ^1H NMR (500 MHz, $CDCl_3$): δ 7.70-7.58 (m, 5 H), 3.79-3.74 (m, 2H), 3.20 (s, 6H), 2.26-2.23 (m, 2H), 1.35 (s, 3H); ^{13}C NMR {^1H} (125 MHz, $CDCl_3$): δ 153.5, 133.2, 131.6, 129.9, 125.2, 100.2, 52.6, 48.7, 29.3, 21.2; HRMS (FTMS + p ESI) *m/z*: $[M+Na]^+$ Calcd for $C_{13}H_{18}O_4N_4NaS$ 349.0941; Found 349.0933.

[xiii] Ketalsulfone **14** is prone to decomposition. It should be used immediately or stored at –20 °C.

References

1. Goossen, L. J.; Paetzold, J.; Koley, D. Regiocontrolled Ru-catalyzed addition of carboxylic acids to alkynes: practical protocols for the synthesis of vinyl esters. *Chem. Commun.* **2003**, 706-707.
2. Knight, D. W.; Morgan, I. R.; Proctor, A. J., A simple oxidative procedure for the removal of ruthenium residues from metathesis reaction products. *Tetrahedron Lett.* **2010**, *51*, 638-640.
3. Matsumoto, K.; Feng, C.; Handa, S.; Oguma, T.; Katsuki, T. Asymmetric epoxidation of (Z)-enol esters catalyzed by titanium(salalen) complex with aqueous hydrogen peroxide. *Tetrahedron* **2011**, *67*, 6474-6478.
4. Doucet, H.; Martin-Vaca, B.; Bruneau, C.; Dixneuf, P. H. General Synthesis of (Z)-Alk-1-en-1-yl Esters via Ruthenium-Catalyzed anti-Markovnikov trans-Addition of Carboxylic Acids to Terminal Alkynes. *J. Org. Chem.* **1995**, *60*, 7247-7255.
5. Prepared from diphenylphosphinobutane (dppb) and bis(2-methylallyl)(1,5-cyclooctadiene)ruthenium(II) (both commercially available), as noted in Ref. 4, by the procedure of Genêt et al.: Genêt, J. P.; Pinel, C.; Ratovelomanana-Vidal, V.; Mallart, S.; Pfister, X.; Cano De Andrade, M. C.; Laffitte, J. A. Novel, general synthesis of the chiral catalysts diphosphine-ruthenium (II) diallyl complexes and a new practical in situ preparation of chiral ruthenium (II) catalysts. *Tetrahedron: Asymmetry* **1994**, *5*, 665-614.
6. Szczepaniak, G.; Ruszczyńska, A.; Kosiński, K.; Bulska, E.; Grela, K. Highly efficient and time economical purification of olefin metathesis products from metal residues using an isocyanide scavenger. *Green Chemistry* **2018**, *20*, 1280-1289.
7. Lumbroso, A.; Vautravers, N. R.; Breit, B. Rhodium-Catalyzed Selective anti-Markovnikov Addition of Carboxylic Acids to Alkynes. *Org. Lett.* **2010**, *12*, 5498-5501.
8. Zhou, B.; Chen, H.; Wang, C. Mn-catalyzed aromatic C-H alkenylation with terminal alkynes. *J. Am. Chem. Soc.* **2013**, *135*, 1264-1267.
9. Engler, H.; Lansing, M.; Gordon, C. P.; Neudörfl, J.-M.; Schäfer, M.; Schlörer, N. E.; Copéret, C.; Berkessel, A. Olefin Epoxidation Catalyzed by Titanium–Salalen Complexes: Synergistic H2O2 Activation by Dinuclear Ti Sites, Ligand H-Bonding, and π-Acidity. *ACS Catalysis* **2021**, *11*, 3206-3217.
10. Lansing, M.; Engler, H.; Leuther, T. M.; Neudörfl, J.-M.; Berkessel, A. Titanium cis-1,2-Diaminocyclohexane Salalen Catalysts of Outstanding Activity and Enantioselectivity for the Asymmetric Epoxidation of Nonconjugated Terminal Olefins with Hydrogen Peroxide. *ChemCatChem* **2016**, *8*, 3706-3709.
11. Deffieux, A.; Sepulchre, M.; Spassky, N. Alcoolyse et glycolyse de derives organozinciques et organocadmiens par des composes chiraux. *J. Organometallic Chem.* **1974**, *80*, 311-327.
12. Yamanaka, H.; Sato, K.; Sato, H.; Iida, M.; Oishi, T.; Chida, N. Total synthesis of mycestericin A and its 14-epimer. *Tetrahedron* **2009**, *65*, 9188-9201.
13. Negishi has validated the use of MTPA esters to measure enantiopurity of 2-substituted 1-alkanols. Xu, S.; Lee, C.-T.; Wang, G.; Negishi, E. Widely Applicable Synthesis of Enantiomerically Pure Tertiary Alkyl-Containing 1-Alkanols by Zirconium-Catalyzed Asymmetric Carboalumination of Alkenes and Palladium- or Copper-Catalyzed Cross-Coupling. *Chem. Asian J.* **2013**, *8*, 1829-1835.

Spectral Data for New Compounds

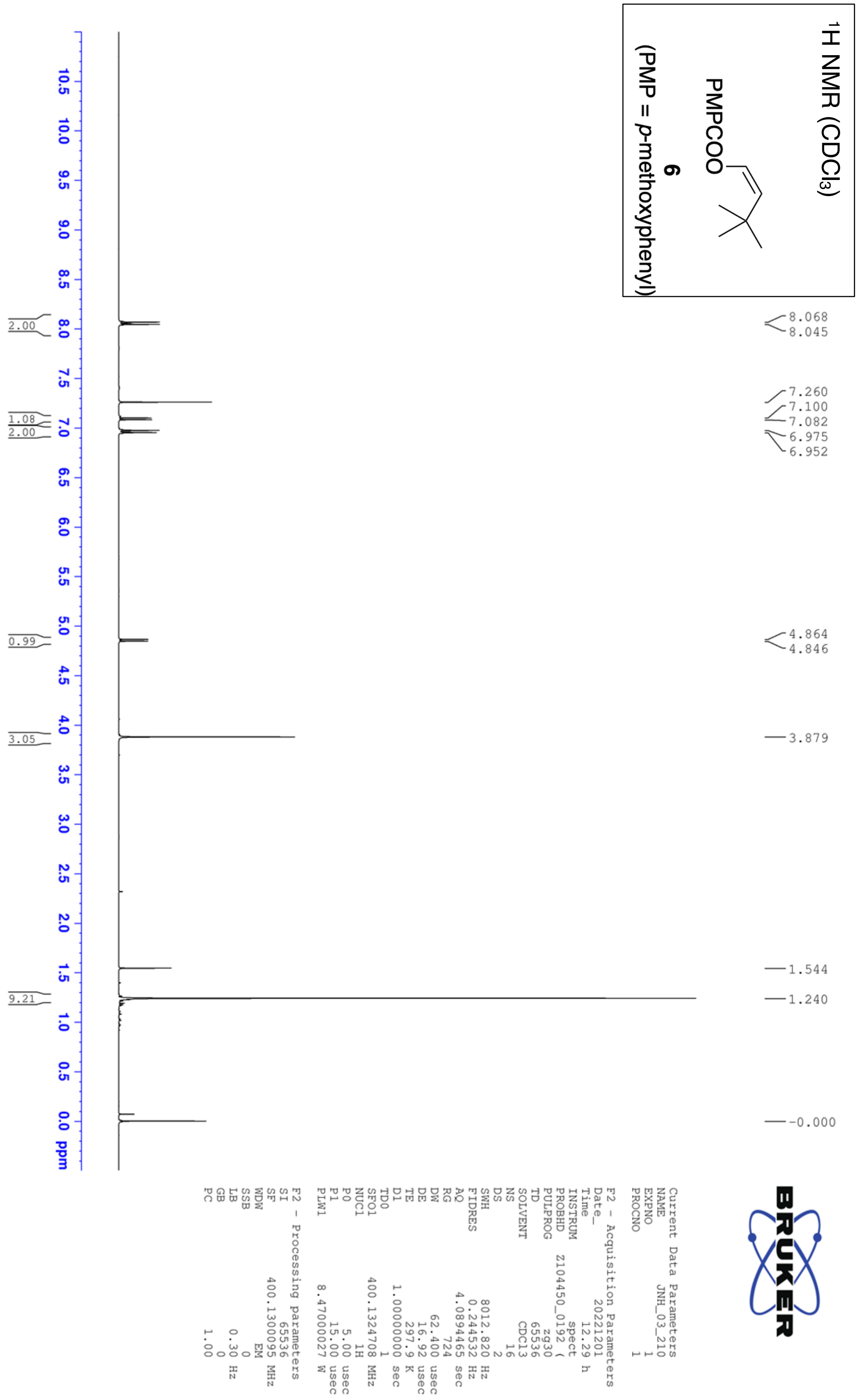

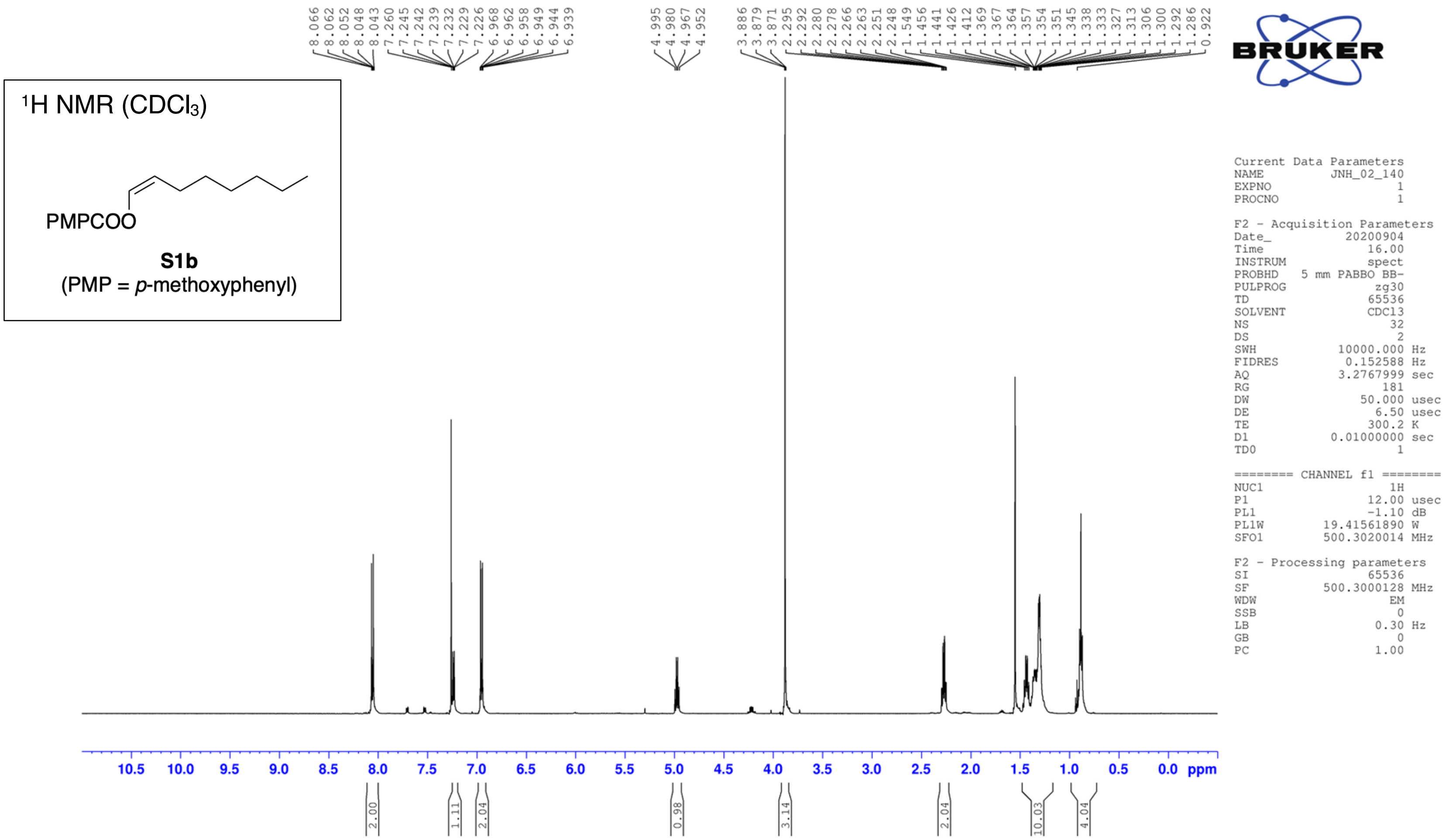

1H NMR (CDCl3)
PMPCOO
S1b
(PMP = p-methoxyphenyl)
8.066
8.062
8.052
8.048
8.043
7.260
7.245
7.242
7.239
7.232
7.229
7.226
6.968
6.962
6.958
6.949
6.944
6.939
4.995
4.980
4.967
4.952
3.886
3.879
3.871
2.295
2.292
2.280
2.278
2.266
2.263
2.251
2.248
1.549
1.456
1.441
1.426
1.412
1.369
1.367
1.364
1.357
1.354
1.351
1.345
1.338
1.333
1.327
1.313
1.306
1.300
1.292
1.286
0.922
10.5
10.0
9.5
9.0
8.5
8.0
7.5
7.0
6.5
6.0
5.5
5.0
4.5
4.0
3.5
3.0
2.5
2.0
1.5
1.0
0.5
0.0 ppm
2.00
1.11
2.04
0.98
3.14
2.04
10.03
4.04
Current Data Parameters
NAME JNH_02_140
EXPNO 1
PROCNO 1
F2 - Acquisition Parameters
Date_ 20200904
Time 16.00
INSTRUM spect
PROBHD 5 mm PABBO BB-
PULPROG zg30
TD 65536
SOLVENT CDCl3
NS 32
DS 2
SWH 10000.000 Hz
FIDRES 0.152588 Hz
AQ 3.2767999 sec
RG 181
DW 50.000 usec
DE 6.50 usec
TE 300.2 K
D1 0.01000000 sec
TD0 1
======== CHANNEL f1 ========
NUC1 1H
P1 12.00 usec
PL1 -1.10 dB
PL1W 19.41561890 W
SFO1 500.3020014 MHz
F2 - Processing parameters
SI 65536
SF 500.3000128 MHz
WDW EM
SSB 0
LB 0.30 Hz
GB 0
PC 1.00

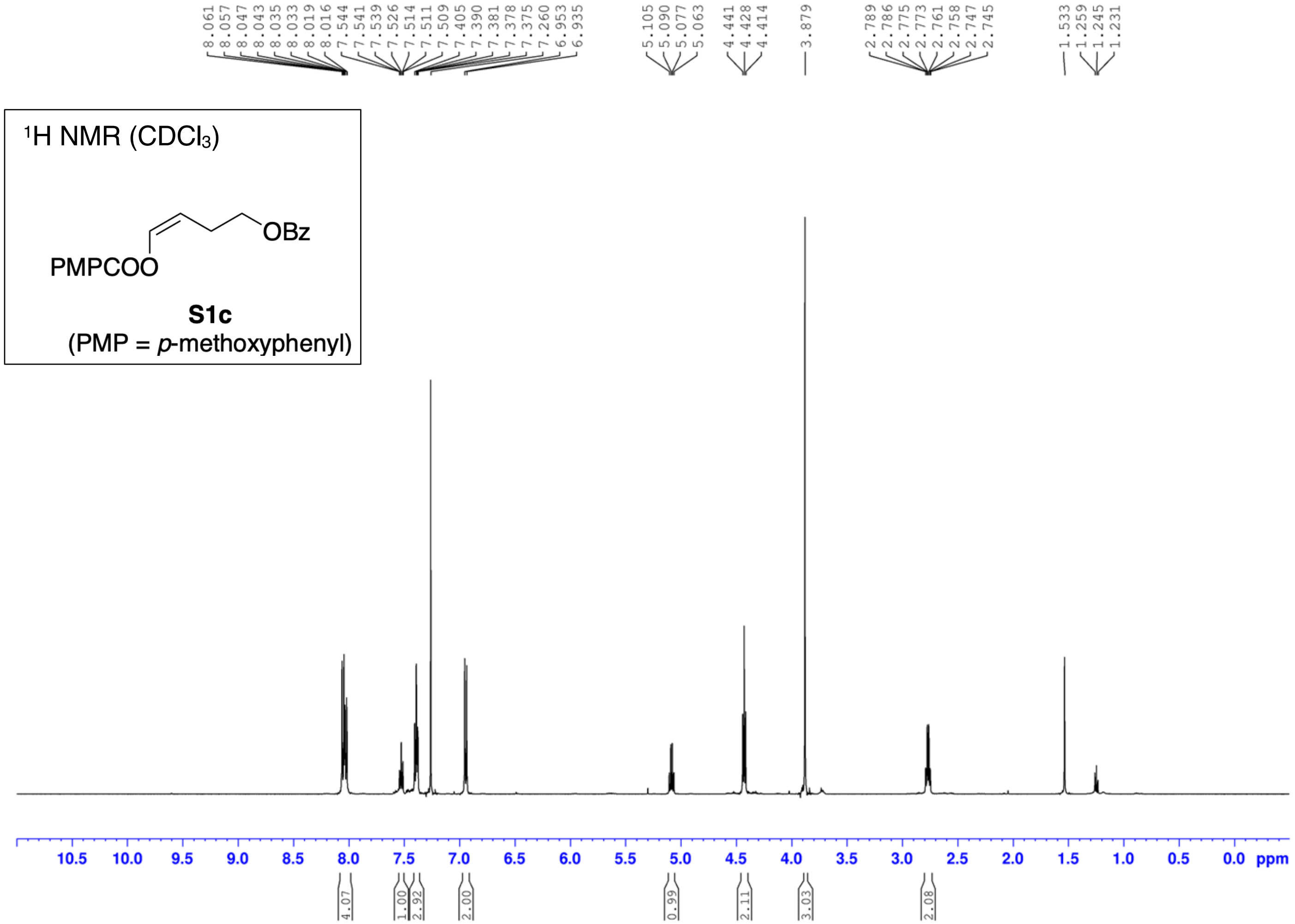


```
Current Data Parameters
NAME             JNH_02_187
EXPNO                     4
PROCNO                    1

F2 - Acquisition Parameters
Date_              20201110
Time                  16.25
INSTRUM               spect
PROBHD    5 mm PABBO BB-
PULPROG                zg30
TD                    65536
SOLVENT               CDCl3
NS                       32
DS                        4
SWH           10000.000 Hz
FIDRES         0.152588 Hz
AQ            3.2767999 sec
RG                    322.5
DW               50.000 usec
DE                 6.50 usec
TE                300.2 K
D1           0.01000000 sec
TD0                       1

======== CHANNEL f1 ========
NUC1                     1H
P1                12.00 usec
PL1               -1.10 dB
PL1W        19.41561890 W
SFO1        500.3020014 MHz

F2 - Processing parameters
SI                    65536
SF          500.3000129 MHz
WDW                      EM
SSB                       0
LB                   0.30 Hz
GB                        0
PC                     1.00
```

^{13}C NMR ($CDCl_3$)

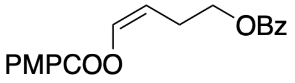

S1c
(PMP = *p*-methoxyphenyl)

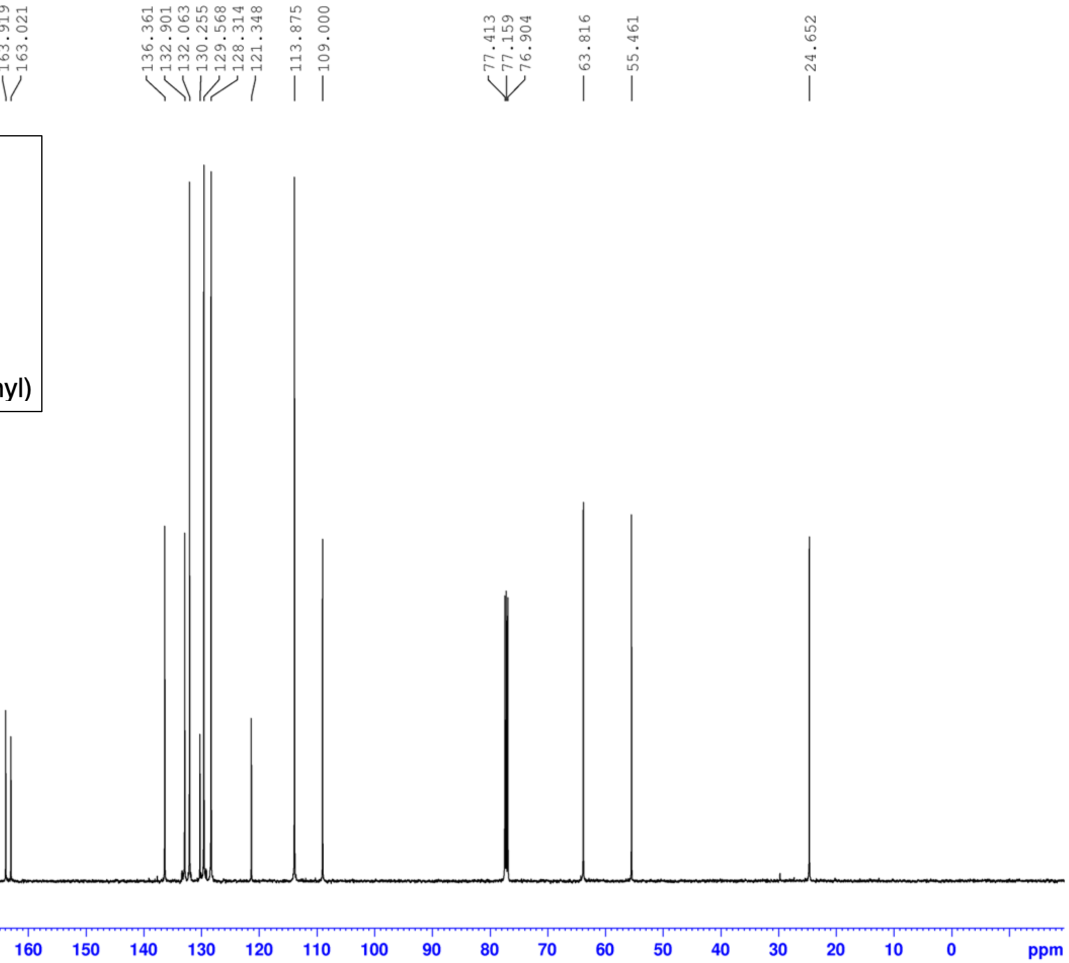


```
Current Data Parameters
NAME              JNH_02_187
EXPNO                      8
PROCNO                     1

F2 - Acquisition Parameters
Date_               20201116
Time                   11.40
INSTRUM                spect
PROBHD     5 mm PABBO BB-
PULPROG              zgpg30
TD                    131072
SOLVENT                CDCl3
NS                       256
DS                         8
SWH                31446.541 Hz
FIDRES              0.239918 Hz
AQ                 2.0840447 sec
RG                   20642.5
DW                    15.900 usec
DE                      6.50 usec
TE                     300.2 K
D1                2.00000000 sec
D11               0.03000000 sec
TD0                        1

======== CHANNEL f1 ========
NUC1                     13C
P1                     10.00 usec
PL1                    -1.70 dB
PL1W            148.61408997 W
SFO1             125.8131151 MHz

======== CHANNEL f2 ========
CPDPRG[2             waltz16
NUC2                      1H
PCPD2                  80.00 usec
PL2                    -1.10 dB
PL12                   15.40 dB
PL13                   17.40 dB
PL2W             19.41561890 W
PL12W             0.43466163 W
PL13W             0.27425295 W
SFO2             500.3025017 MHz

F2 - Processing parameters
SI                    131072
SF               125.8005351 MHz
WDW                       EM
SSB                        0
LB                      3.00 Hz
GB                         0
PC                      1.40
```

^{1}H NMR ($CDCl_3$)

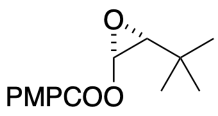

7

(PMP = *p*-methoxyphenyl)

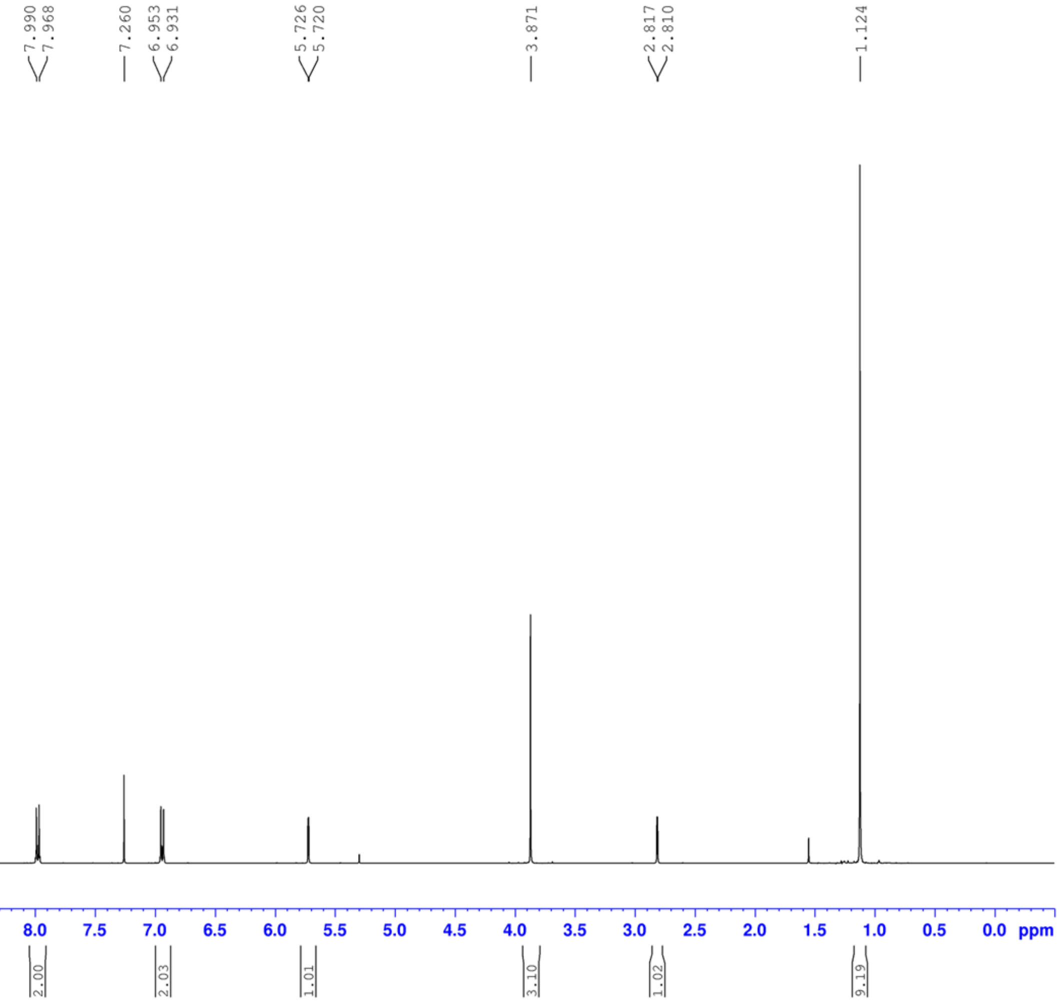


```
Current Data Parameters
NAME            JNH_02_72
EXPNO                   1
PROCNO                  1

F2 - Acquisition Parameters
Date_            20191219
Time                13.47 h
INSTRUM             spect
PROBHD    Z104450_0192 (
PULPROG              zg30
TD                  65536
SOLVENT             CDCl3
NS                     16
DS                      2
SWH              8012.820 Hz
FIDRES           0.244532 Hz
AQ              4.0894465 sec
RG                    575
DW                 62.400 usec
DE                  16.92 usec
TE                  297.1 K
D1             1.00000000 sec
TD0                     1
SFO1          400.1324708 MHz
NUC1                   1H
P0                   5.00 usec
P1                  15.00 usec
PLW1           8.47000027 W

F2 - Processing parameters
SI                  65536
SF            400.1300097 MHz
WDW                    EM
SSB                     0
LB                   0.30 Hz
GB                      0
PC                   1.00
```

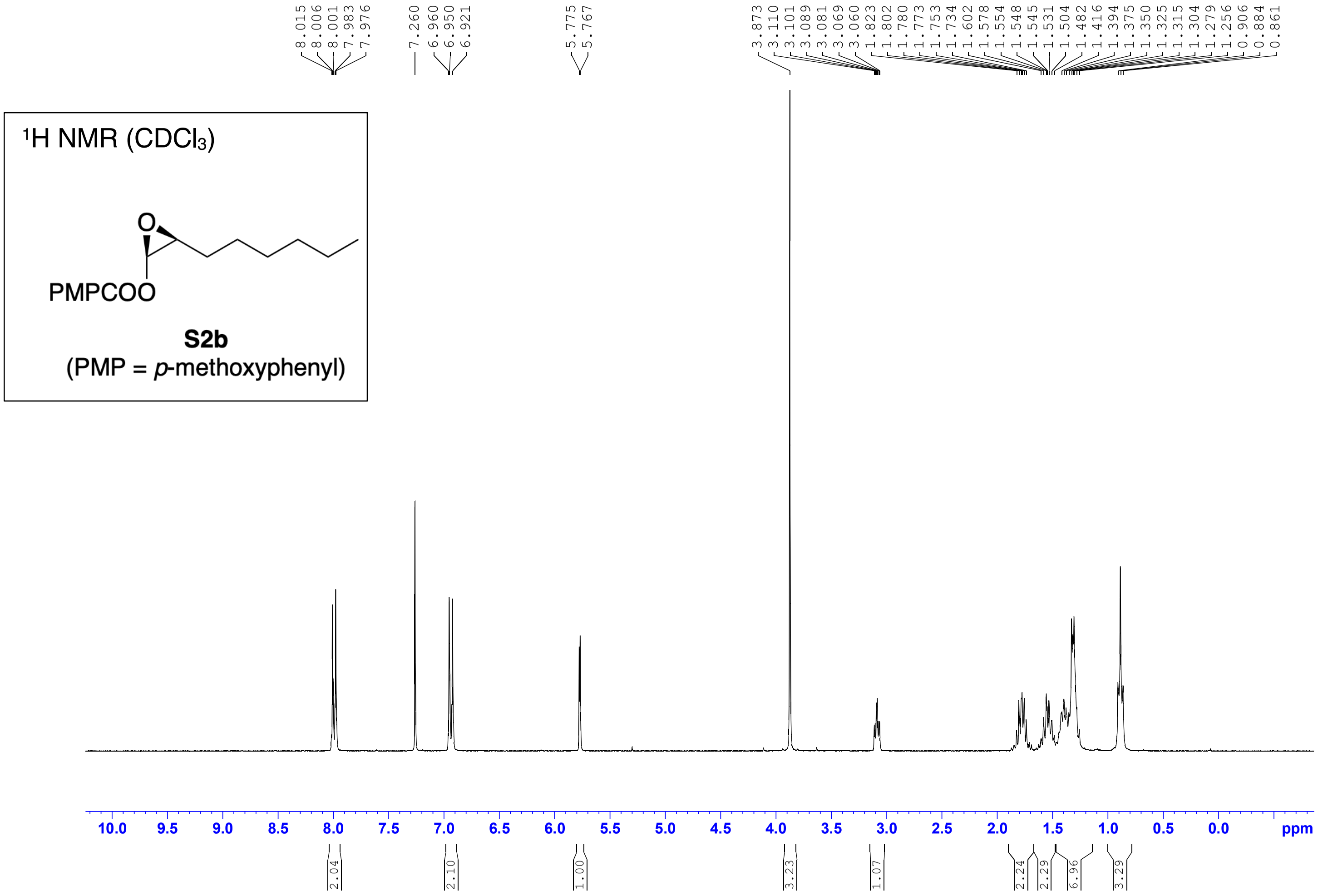


```
Current Data Parameters
NAME              JNH_02_6
EXPNO                    1
PROCNO                   1

F2 - Acquisition Parameters
Date_             20190613
Time                 16.31
INSTRUM              spect
PROBHD   5 mm QNP 1H/13
PULPROG               zg30
TD                   32768
SOLVENT              CDCl3
NS                      16
DS                       0
SWH               5995.204 Hz
FIDRES            0.182959 Hz
AQ               2.7328513 sec
RG                   812.7
DW                  83.400 usec
DE                    6.50 usec
TE                   300.0 K
D1              0.01000000 sec
TD0                      1

======== CHANNEL f1 ========
NUC1                    1H
P1                    9.00 usec
PL1                  -0.50 dB
SFO1           300.1524012 MHz

F2 - Processing parameters
SI                   16384
SF             300.1500051 MHz
WDW                     EM
SSB                      0
LB                    0.30 Hz
GB                       0
PC                    1.00
```

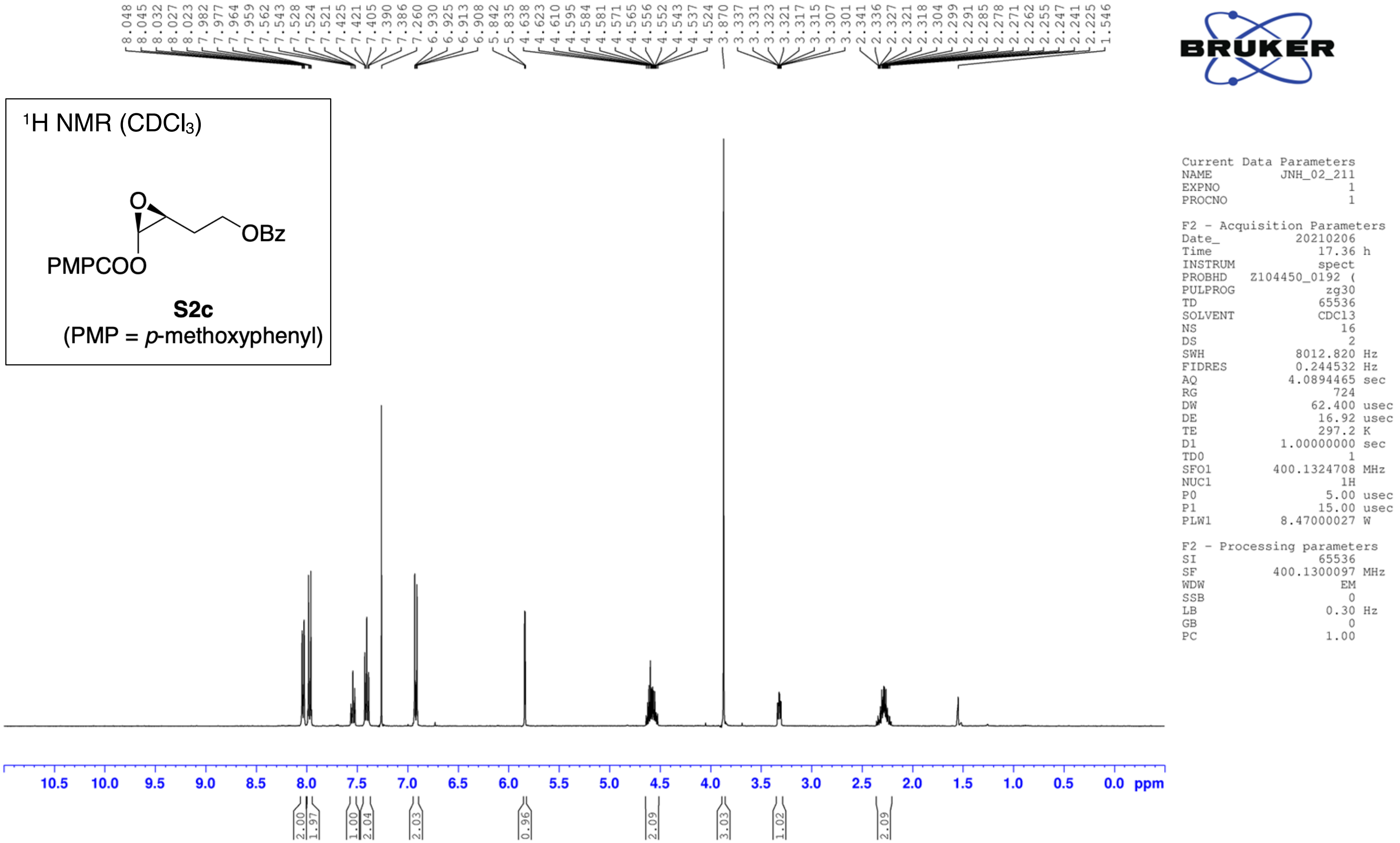

1H NMR (CDCl3)
PMPCOO
OBz
O
S2c
(PMP = p-methoxyphenyl)
8.048 8.045 8.032 8.027 8.023 7.982 7.977 7.964 7.959 7.562 7.543 7.528 7.524 7.521 7.425 7.421 7.405 7.390 7.386 7.260 6.930 6.925 6.913 6.908 5.842 5.835 4.638 4.623 4.610 4.595 4.584 4.581 4.571 4.565 4.556 4.552 4.543 4.537 4.524 3.870 3.337 3.331 3.323 3.321 3.317 3.315 3.307 3.301 2.341 2.336 2.327 2.321 2.318 2.304 2.299 2.291 2.285 2.278 2.271 2.262 2.255 2.247 2.241 2.225 1.546
2.00 1.97 1.00 2.04 2.03 0.96 2.09 3.03 1.02 2.09
10.5 10.0 9.5 9.0 8.5 8.0 7.5 7.0 6.5 6.0 5.5 5.0 4.5 4.0 3.5 3.0 2.5 2.0 1.5 1.0 0.5 0.0 ppm
Current Data Parameters
NAME JNH_02_211
EXPNO 1
PROCNO 1
F2 - Acquisition Parameters
Date_ 20210206
Time 17.36 h
INSTRUM spect
PROBHD Z104450_0192 (
PULPROG zg30
TD 65536
SOLVENT CDCl3
NS 16
DS 2
SWH 8012.820 Hz
FIDRES 0.244532 Hz
AQ 4.0894465 sec
RG 724
DW 62.400 usec
DE 16.92 usec
TE 297.2 K
D1 1.00000000 sec
TD0 1
SFO1 400.1324708 MHz
NUC1 1H
P0 5.00 usec
P1 15.00 usec
PLW1 8.47000027 W
F2 - Processing parameters
SI 65536
SF 400.1300097 MHz
WDW EM
SSB 0
LB 0.30 Hz
GB 0
PC 1.00

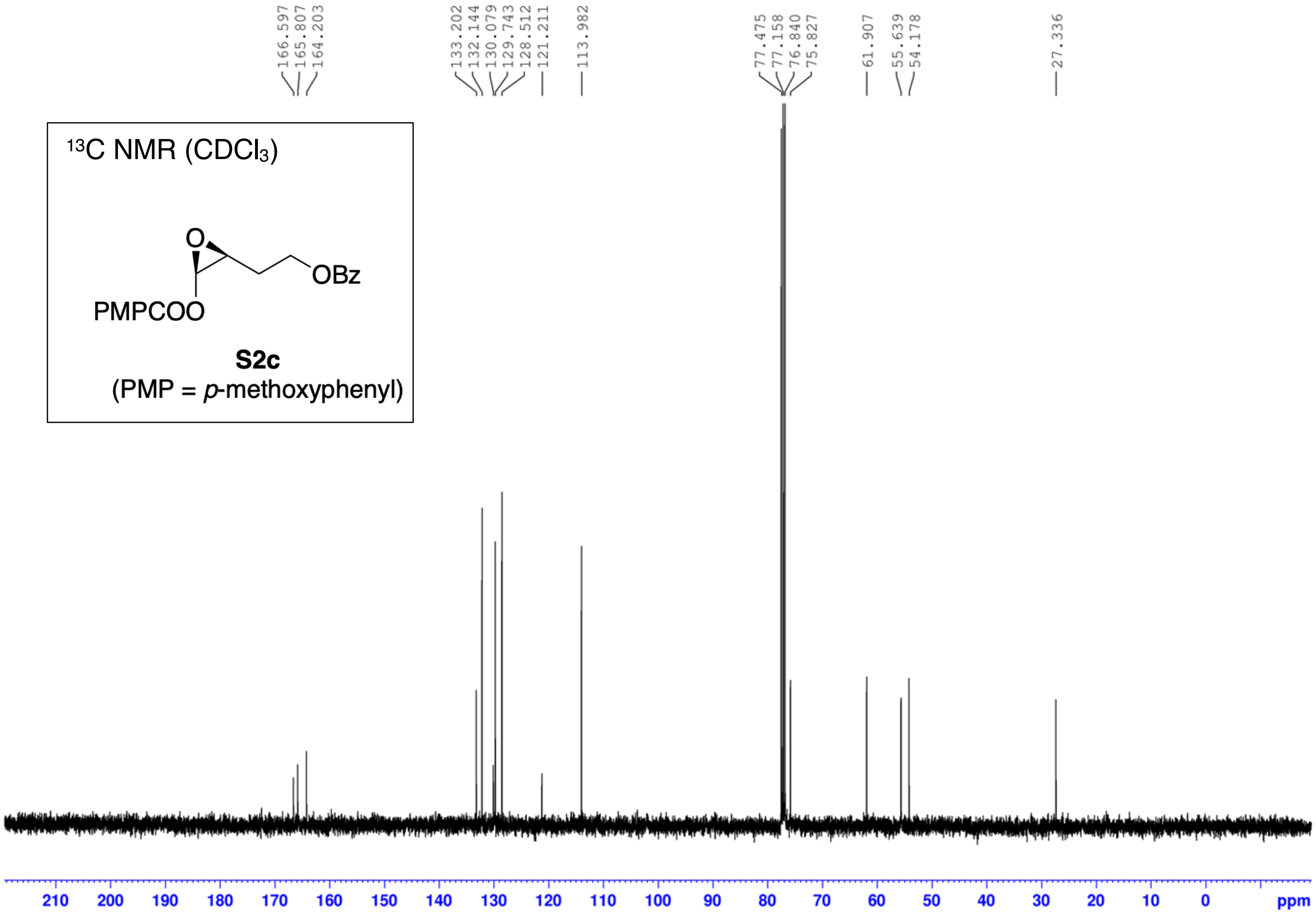

Current Data Parameters
NAME JNH_03_3 13C
EXPNO 1
PROCNO 1

F2 - Acquisition Parameters
Date_ 20220527
Time 19.58 h
INSTRUM spect
PROBHD Z104450_0192 (
PULPROG zgpg30
TD 65536
SOLVENT CDCl3
NS 101
DS 2
SWH 24038.461 Hz
FIDRES 0.733596 Hz
AQ 1.3631488 sec
RG 203
DW 20.800 usec
DE 6.50 usec
TE 298.0 K
D1 2.00000000 sec
D11 0.03000000 sec
TD0 1
SFO1 100.6228298 MHz
NUC1 13C
P0 3.28 usec
P1 9.85 usec
PLW1 28.63999939 W
SFO2 400.1316005 MHz
NUC2 1H
CPDPRG[2 waltz65
PCPD2 90.00 usec
PLW2 8.47000027 W
PLW12 0.23528001 W
PLW13 0.11834000 W

F2 - Processing parameters
SI 32768
SF 100.6127561 MHz
WDW EM
SSB 0
LB 1.00 Hz
GB 0
PC 1.40

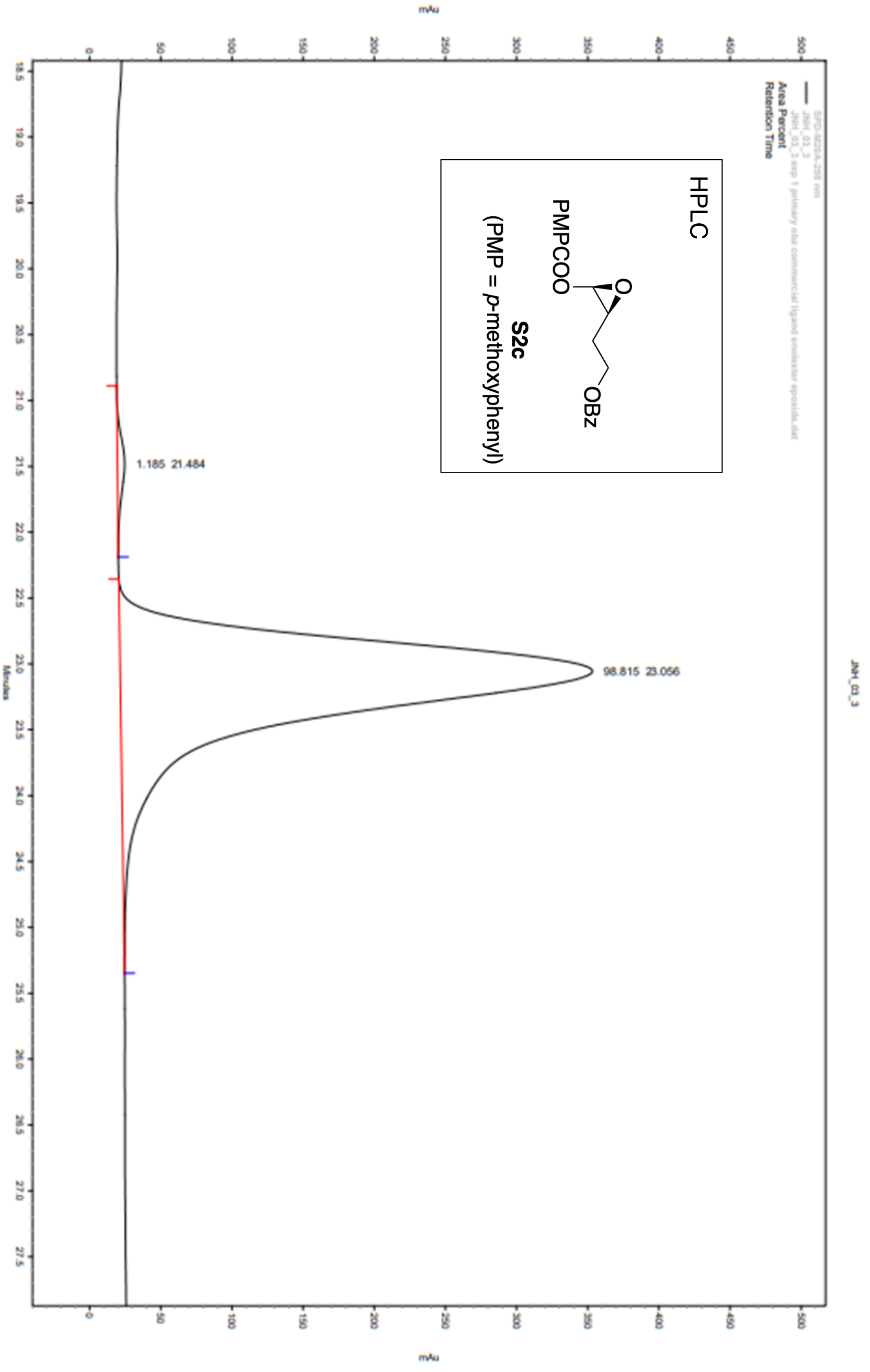

HPLC
PMPCOO
O
OBz
S2c
(PMP = p-methoxyphenyl)
Area Percent
Retention Time
1.185 21.484
98.815 23.056
JNH_03_3
mAu
Minutes

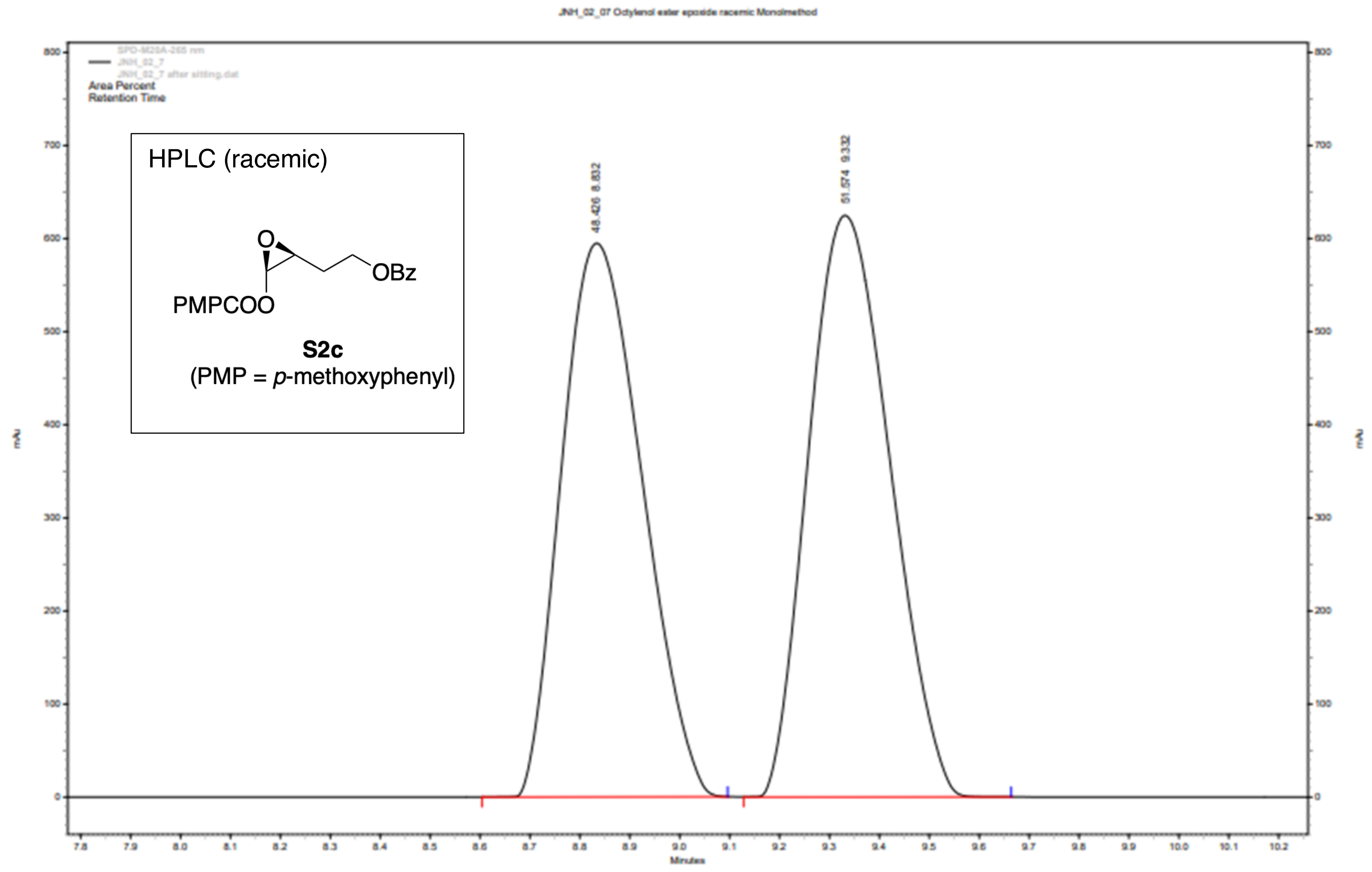

JNH_02_07 Octylenol ester epoxide racemic Monolmethod
Area Percent
Retention Time
HPLC (racemic)
O
PMPCOO
OBz
S2c
(PMP = p-methoxyphenyl)
48.426 8.832
51.574 9.332
mAu
Minutes

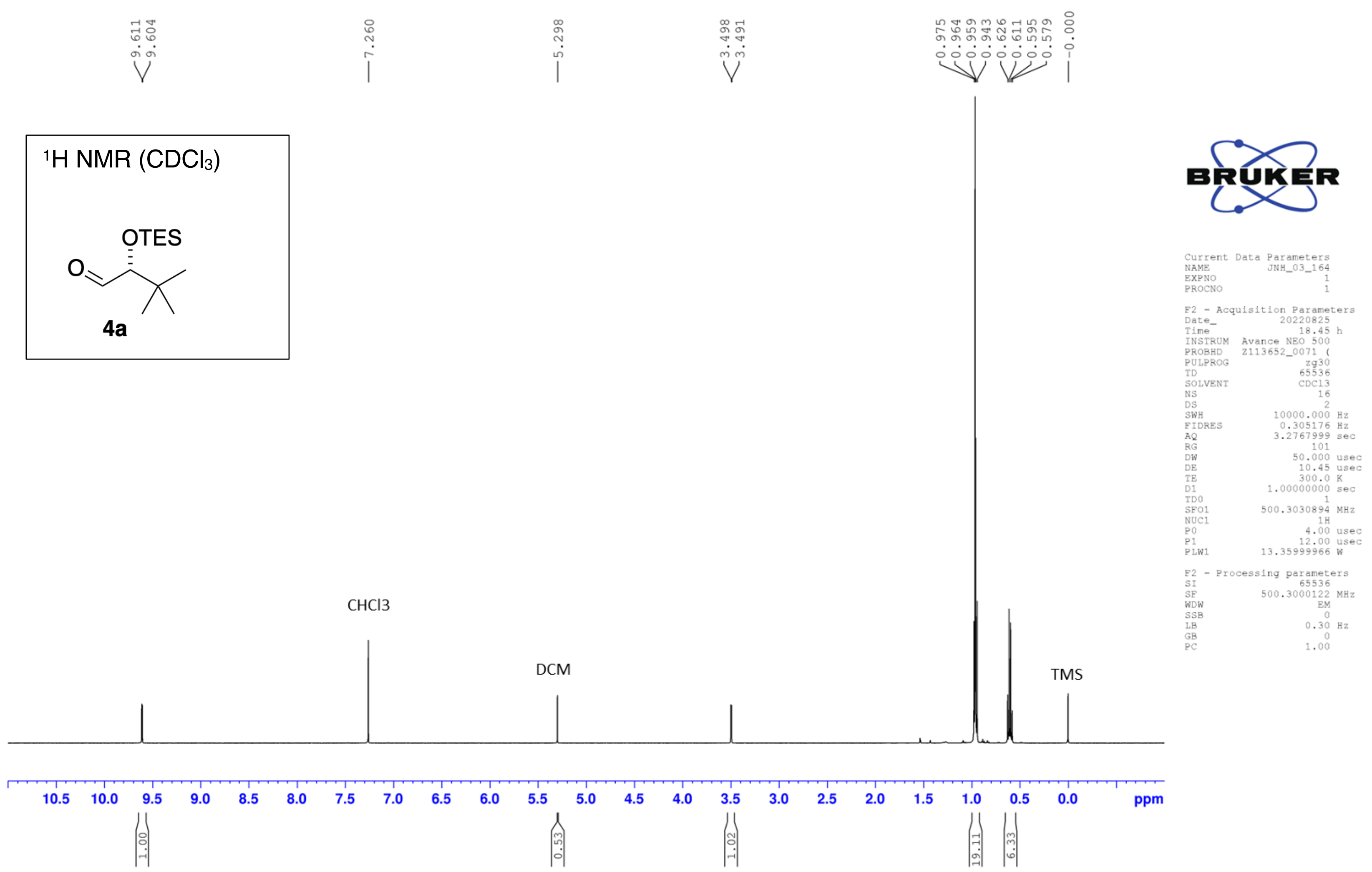

1H NMR (CDCl3)
OTES
O
4a
9.611
9.604
7.260
5.298
3.498
3.491
0.975
0.964
0.959
0.943
0.626
0.611
0.595
0.579
-0.000
CHCl3
DCM
TMS
10.5 10.0 9.5 9.0 8.5 8.0 7.5 7.0 6.5 6.0 5.5 5.0 4.5 4.0 3.5 3.0 2.5 2.0 1.5 1.0 0.5 0.0 ppm
1.00
0.53
1.02
19.11
6.33
BRUKER
Current Data Parameters
NAME JNH_03_164
EXPNO 1
PROCNO 1
F2 - Acquisition Parameters
Date_ 20220825
Time 18.45 h
INSTRUM Avance NEO 500
PROBHD Z113652_0071 (
PULPROG zg30
TD 65536
SOLVENT CDCl3
NS 16
DS 2
SWH 10000.000 Hz
FIDRES 0.305176 Hz
AQ 3.2767999 sec
RG 101
DW 50.000 usec
DE 10.45 usec
TE 300.0 K
D1 1.00000000 sec
TD0 1
SFO1 500.3030894 MHz
NUC1 1H
P0 4.00 usec
P1 12.00 usec
PLW1 13.35999966 W
F2 - Processing parameters
SI 65536
SF 500.3000122 MHz
WDW EM
SSB 0
LB 0.30 Hz
GB 0
PC 1.00

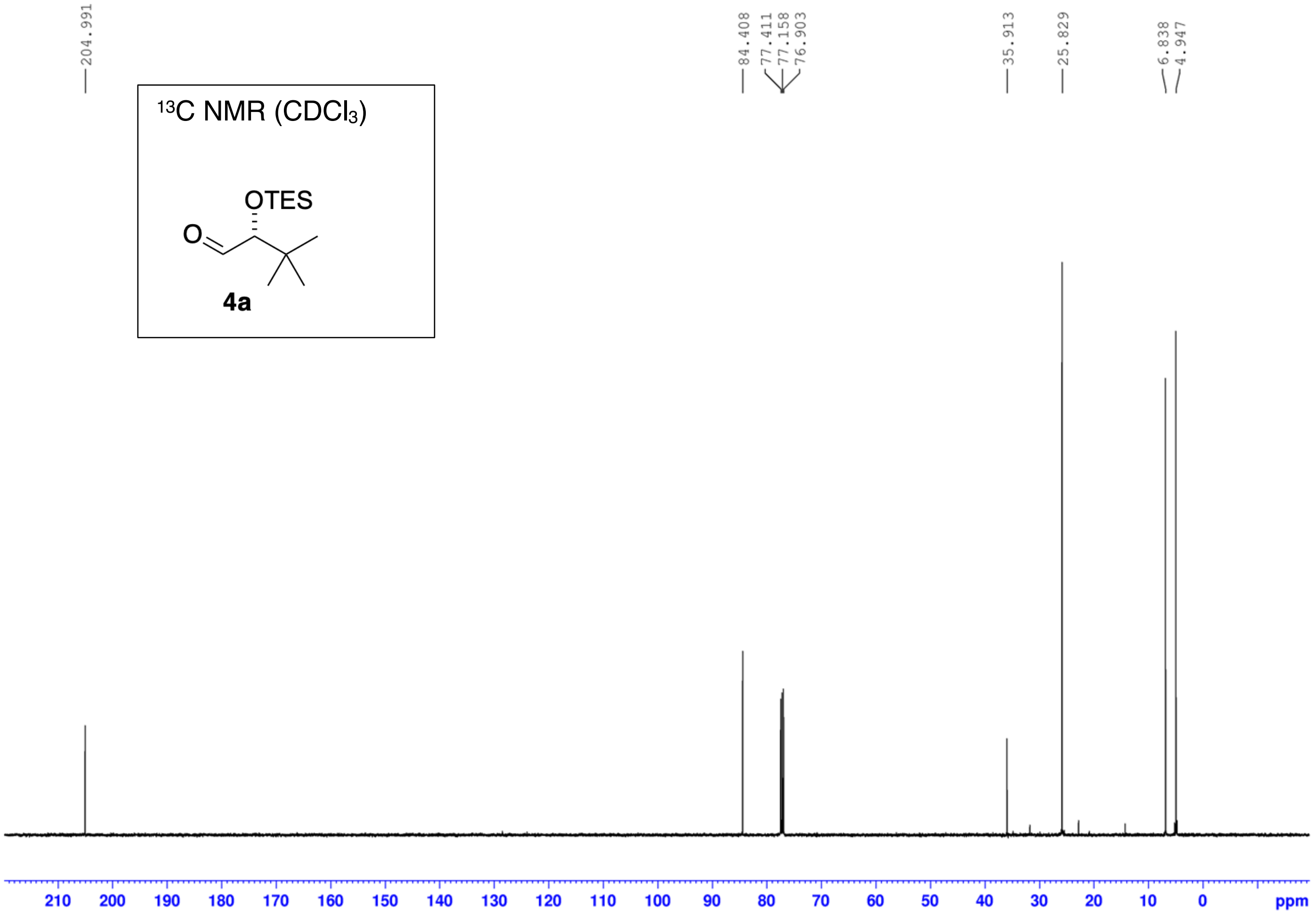

BRUKER

```
Current Data Parameters
NAME         JNH_03_16 13C
EXPNO                    3
PROCNO                   1

F2 - Acquisition Parameters
Date_             20211112
Time               10.49 h
INSTRUM     Avance NEO 500
PROBHD      Z113652_0071 (
PULPROG            zgpg30
TD                  65536
SOLVENT             CDCl3
NS                     92
DS                      4
SWH            30120.482 Hz
FIDRES          0.919204 Hz
AQ             1.0878977 sec
RG                    101
DW                 16.600 usec
DE                  10.00 usec
TE                  298.0 K
D1             2.00000000 sec
D11            0.03000000 sec
TD0                     1
SFO1          125.8131151 MHz
NUC1                  13C
P0                   3.33 usec
P1                  10.00 usec
PLW1         103.61000061 W
SFO2          500.3020012 MHz
NUC2                   1H
CPDPRG[2           waltz65
PCPD2               80.00 usec
PLW2          13.35999966 W
PLW12          0.30400079 W
PLW13          0.15236519 W

F2 - Processing parameters
SI                  32768
SF            125.8005170 MHz
WDW                    EM
SSB                     0
LB                   1.00 Hz
GB                      0
PC                   1.40
```

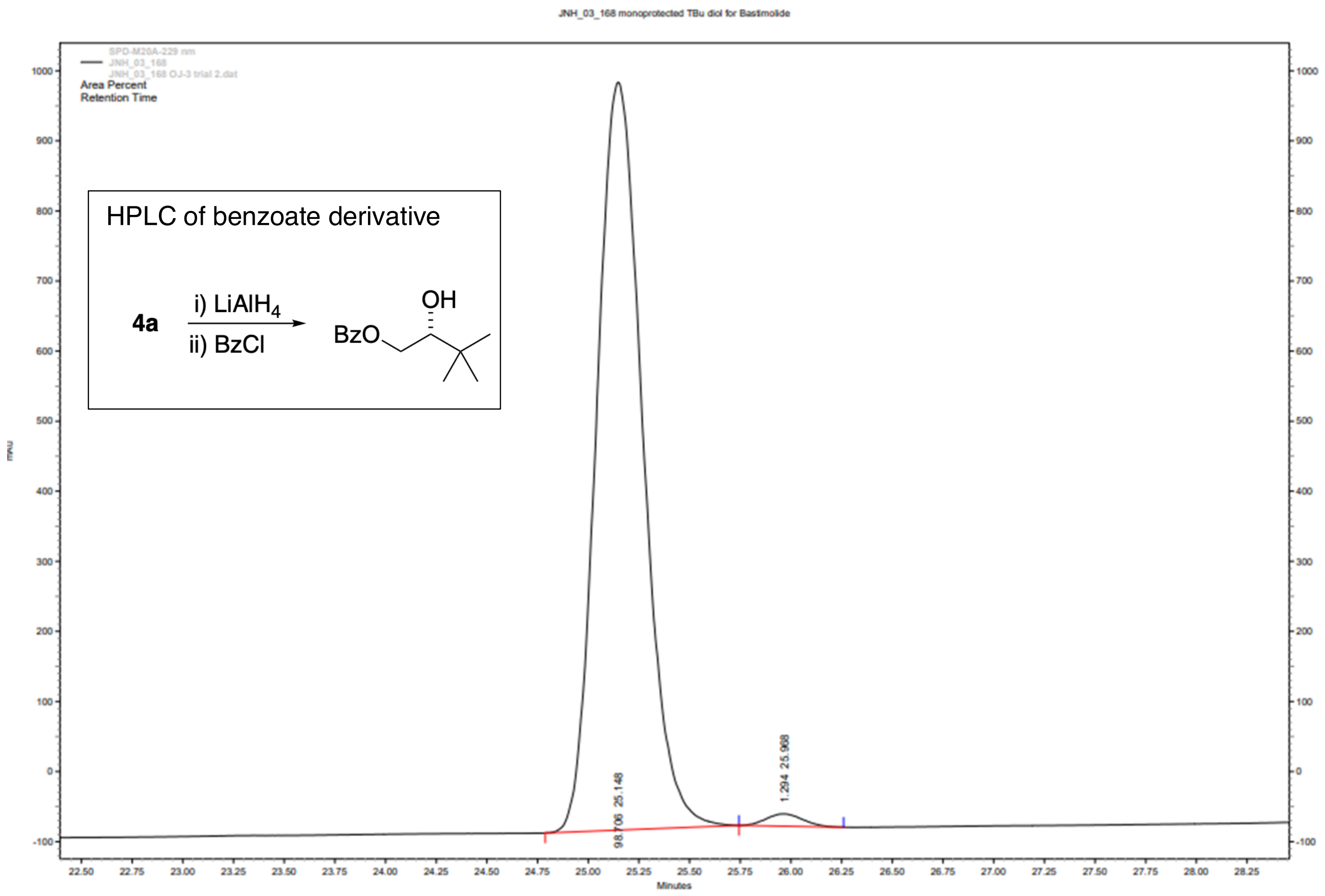

JNH_03_168 monoprotected TBu diol for Bastimolide
SPD-M20A-229 nm
JNH_03_168
JNH_03_168 OJ-3 trial 2.dat
Area Percent
Retention Time
HPLC of benzoate derivative
4a
i) LiAlH4
ii) BzCl
BzO
OH
98.706 25.148
1.294 25.968
mAU
Minutes

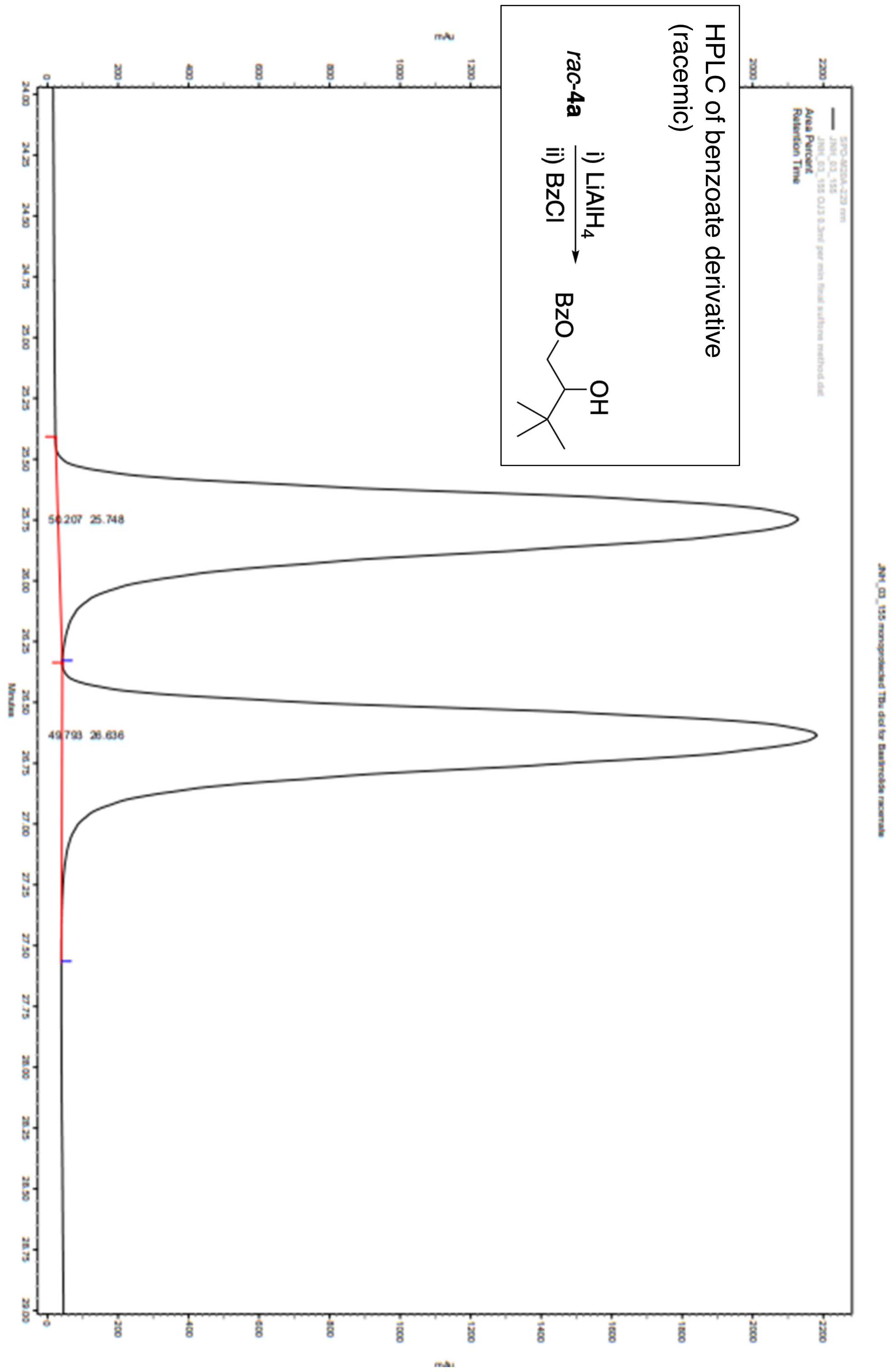
HPLC of benzoate derivative (racemic)
rac-4a
i) LiAlH4
ii) BzCl
BzO
OH
56.207 25.748
49.793 26.636
Minutes
mAu

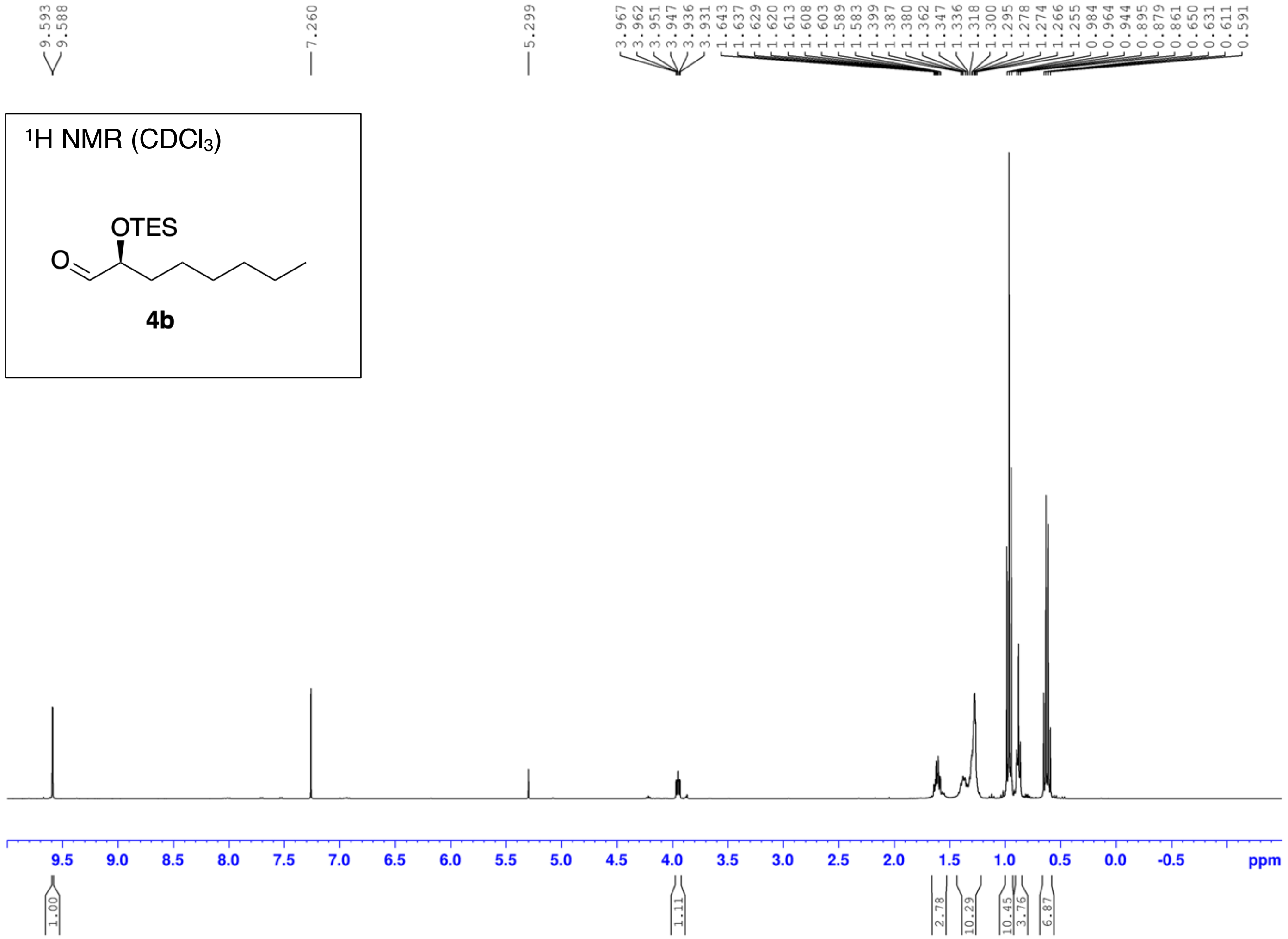

Current Data Parameters

NAME	JNH_02_229
EXPNO	1
PROCNO	1

F2 - Acquisition Parameters

Date_	20210323
Time	8.01 h
INSTRUM	spect
PROBHD	Z104450_0192 (
PULPROG	zg30
TD	65536
SOLVENT	CDCl3
NS	32
DS	2
SWH	8012.820 Hz
FIDRES	0.244532 Hz
AQ	4.0894465 sec
RG	203
DW	62.400 usec
DE	16.92 usec
TE	296.0 K
D1	1.00000000 sec
TD0	1
SFO1	400.1324708 MHz
NUC1	1H
P0	5.00 usec
P1	15.00 usec
PLW1	8.47000027 W

F2 - Processing parameters

SI	65536
SF	400.1300098 MHz
WDW	EM
SSB	0
LB	0.30 Hz
GB	0
PC	1.00

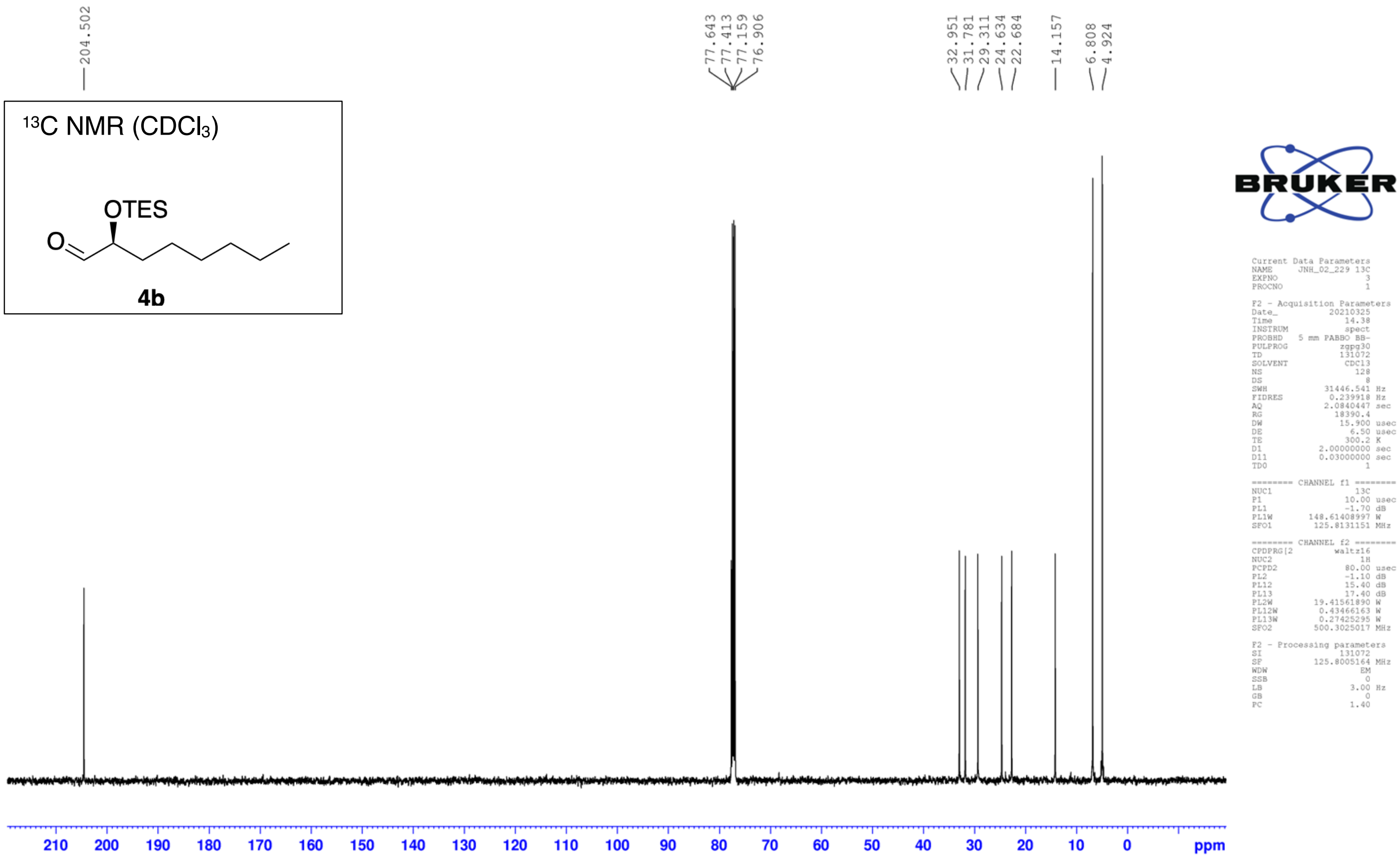

13C NMR (CDCl3)
OTES
O
4b
204.502
77.643
77.413
77.159
76.906
32.951
31.781
29.311
24.634
22.684
14.157
6.808
4.924
210 200 190 180 170 160 150 140 130 120 110 100 90 80 70 60 50 40 30 20 10 0 ppm
BRUKER
Current Data Parameters
NAME JNH_02_229 13C
EXPNO 3
PROCNO 1
F2 - Acquisition Parameters
Date_ 20210325
Time 14.38
INSTRUM spect
PROBHD 5 mm PABBO BB-
PULPROG zgpg30
TD 131072
SOLVENT CDCl3
NS 128
DS 8
SWH 31446.541 Hz
FIDRES 0.239918 Hz
AQ 2.0840447 sec
RG 18390.4
DW 15.900 usec
DE 6.50 usec
TE 300.2 K
D1 2.00000000 sec
D11 0.03000000 sec
TD0 1
======== CHANNEL f1 ========
NUC1 13C
P1 10.00 usec
PL1 -1.70 dB
PL1W 148.61408997 W
SFO1 125.8131151 MHz
======== CHANNEL f2 ========
CPDPRG[2 waltz16
NUC2 1H
PCPD2 80.00 usec
PL2 -1.10 dB
PL12 15.40 dB
PL13 17.40 dB
PL2W 19.41561890 W
PL12W 0.43466163 W
PL13W 0.27425295 W
SFO2 500.3025017 MHz
F2 - Processing parameters
SI 131072
SF 125.8005164 MHz
WDW EM
SSB 0
LB 3.00 Hz
GB 0
PC 1.40

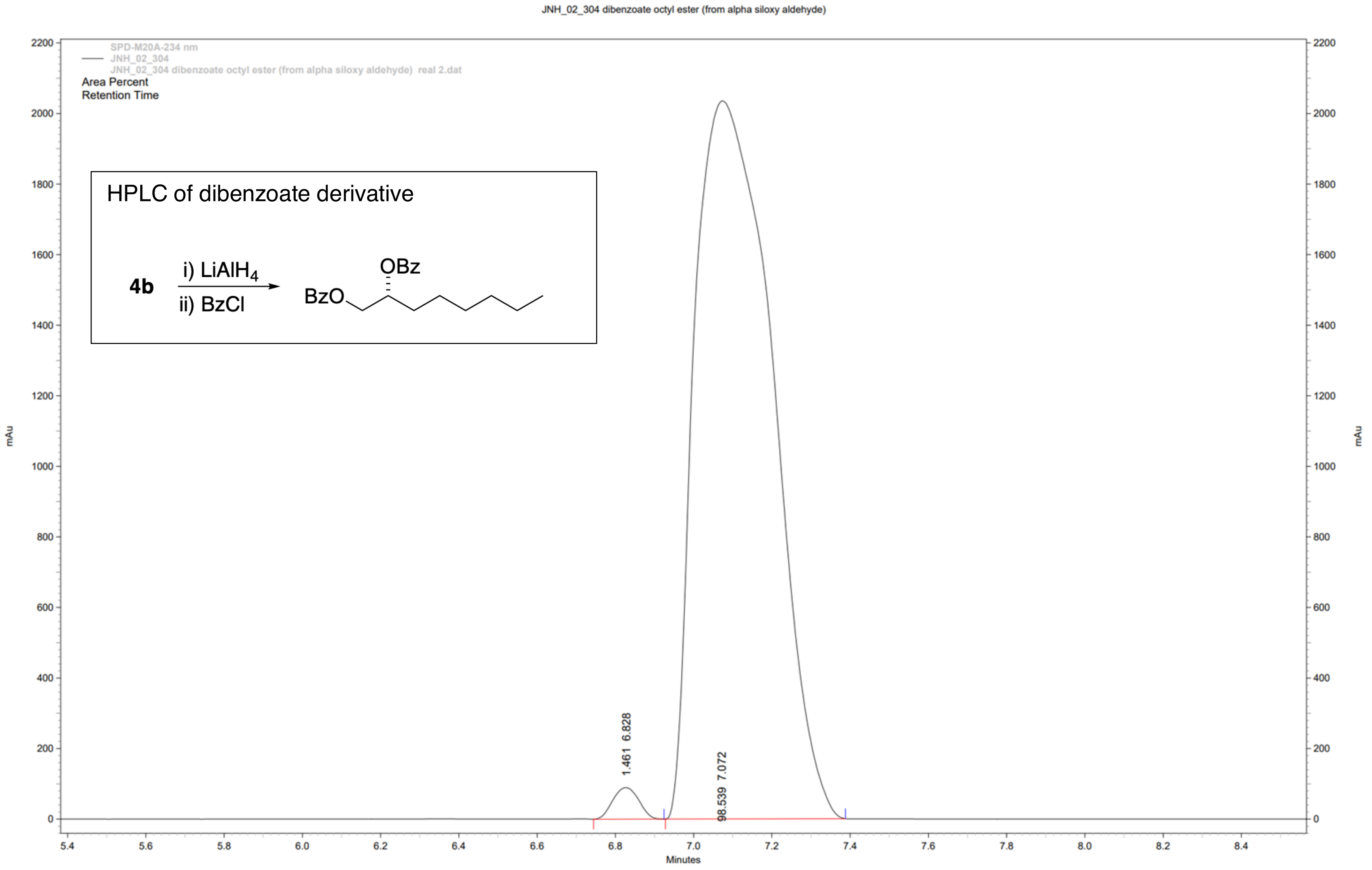
JNH_02_304 dibenzoate octyl ester (from alpha siloxy aldehyde)
SPD-M20A-234 nm
JNH_02_304
JNH_02_304 dibenzoate octyl ester (from alpha siloxy aldehyde) real 2.dat
Area Percent
Retention Time
HPLC of dibenzoate derivative
4b
i) LiAlH4
ii) BzCl
BzO
OBz
1.461 6.828
98.539 7.072
mAu
Minutes

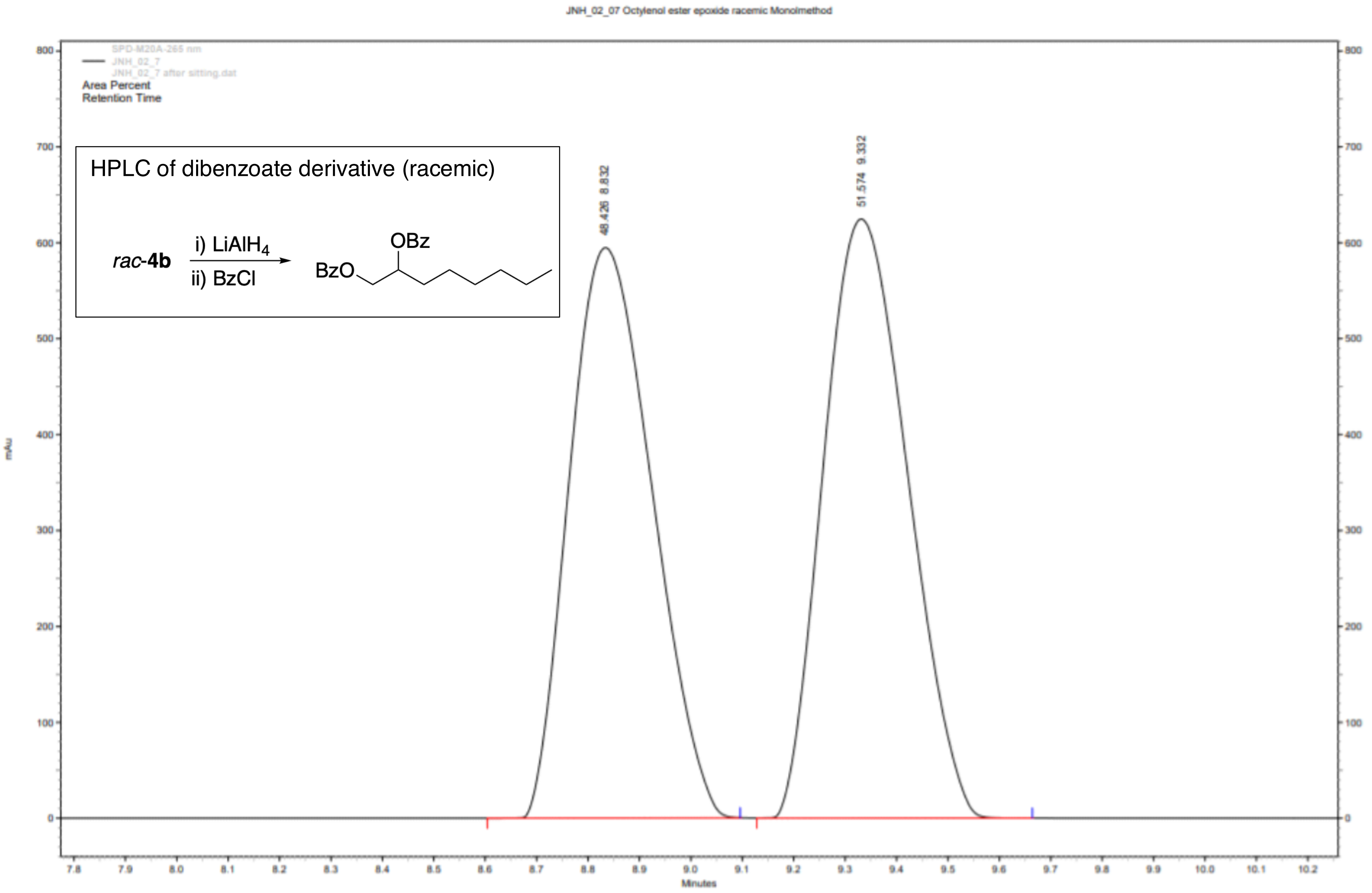
JNH_02_07 Octylenol ester epoxide racemic Monolmethod
SPD-M20A-265 nm
JNH_02_7
JNH_02_7 after sitting.dat
Area Percent
Retention Time
HPLC of dibenzoate derivative (racemic)
rac-4b
i) LiAlH4
ii) BzCl
BzO
OBz
48.426 8.832
51.574 9.332
mAu
Minutes

^1H NMR ($CDCl_3$)

4c

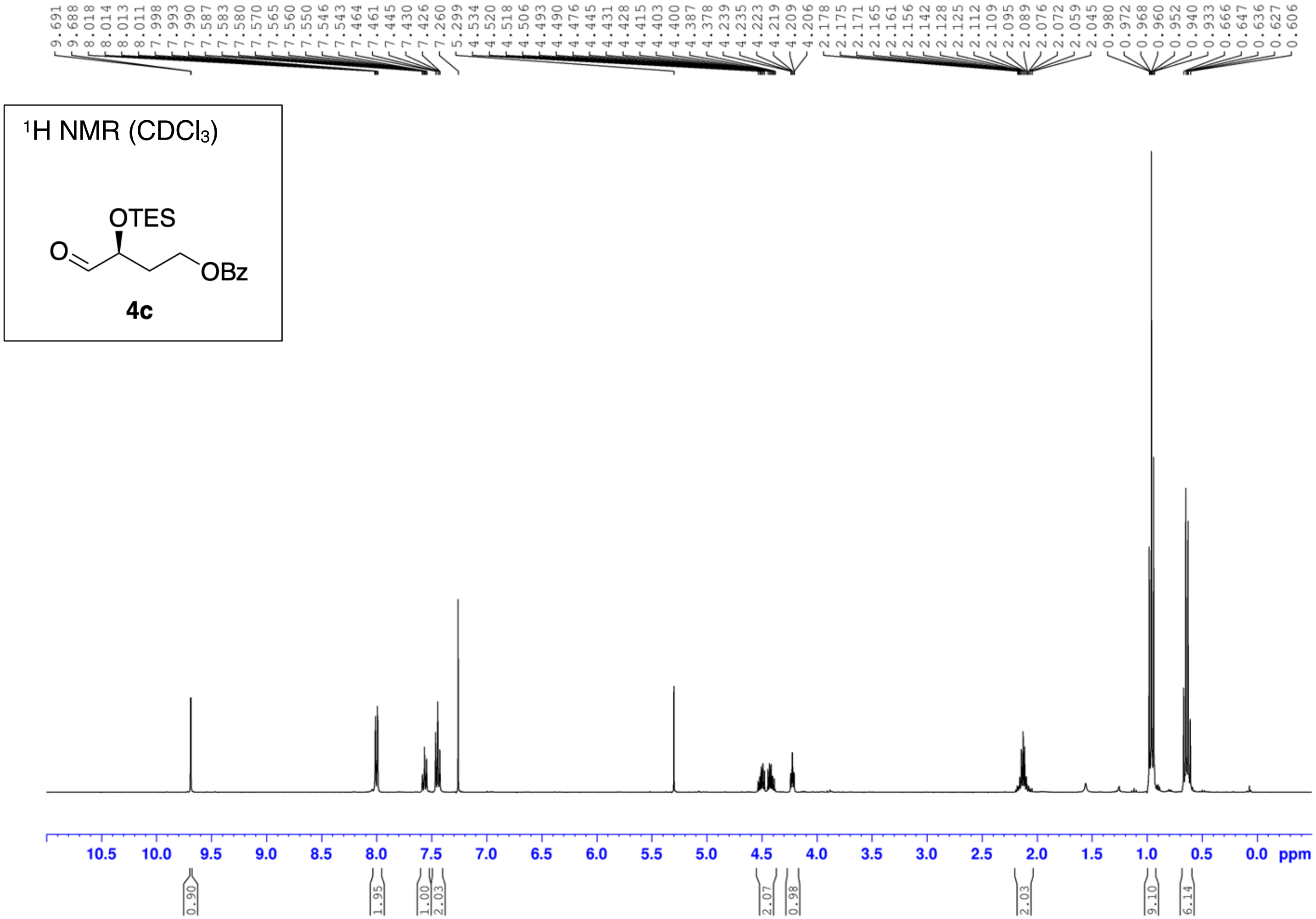

BRUKER

```
Current Data Parameters
NAME             JNH_03_9
EXPNO                   2
PROCNO                  1

F2 - Acquisition Parameters
Date_            20211030
Time                13.43 h
INSTRUM             spect
PROBHD    Z104450_0192 (
PULPROG              zg30
TD                  65536
SOLVENT             CDCl3
NS                     16
DS                      2
SWH              8012.820 Hz
FIDRES           0.244532 Hz
AQ              4.0894465 sec
RG                    512
DW                 62.400 usec
DE                  16.92 usec
TE                  295.6 K
D1             1.00000000 sec
TD0                     1
SFO1          400.1324708 MHz
NUC1                   1H
P0                   5.00 usec
P1                  15.00 usec
PLW1           8.47000027 W

F2 - Processing parameters
SI                  65536
SF            400.1300097 MHz
WDW                    EM
SSB                     0
LB                   0.30 Hz
GB                      0
PC                   1.00
```

^{13}C NMR ($CDCl_3$)

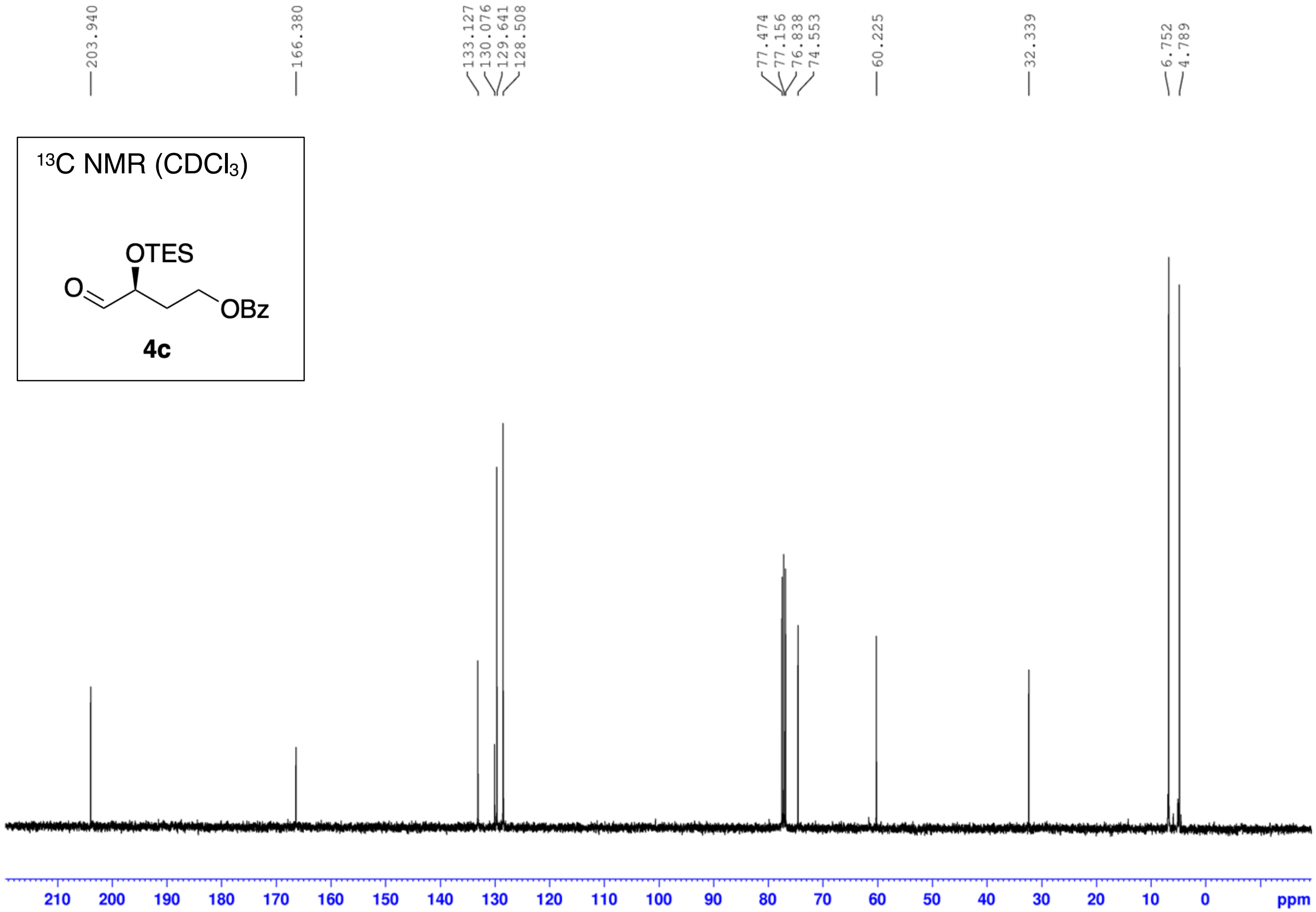

4c

BRUKER

```
Current Data Parameters
NAME       JNH_02_230 13C
EXPNO                   3
PROCNO                  1

F2 - Acquisition Parameters
Date_            20210325
Time               8.58 h
INSTRUM             spect
PROBHD     Z104450_0192 (
PULPROG            zgpg30
TD                  65536
SOLVENT             CDCl3
NS                     64
DS                      2
SWH             24038.461 Hz
FIDRES           0.733596 Hz
AQ              1.3631488 sec
RG                    203
DW                 20.800 usec
DE                   6.50 usec
TE                  297.1 K
D1             2.00000000 sec
D11            0.03000000 sec
TD0                     1
SFO1          100.6228298 MHz
NUC1                  13C
P0                   3.28 usec
P1                   9.85 usec
PLW1          28.63999939 W
SFO2          400.1316005 MHz
NUC2                   1H
CPDPRG[2          waltz65
PCPD2               90.00 usec
PLW2           8.47000027 W
PLW12          0.23528001 W
PLW13          0.11834000 W

F2 - Processing parameters
SI                  32768
SF            100.6127594 MHz
WDW                    EM
SSB                     0
LB                   1.00 Hz
GB                      0
PC                   1.40
```

^1H NMR ($CDCl_3$)

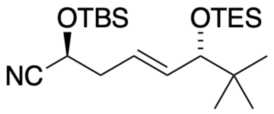

8

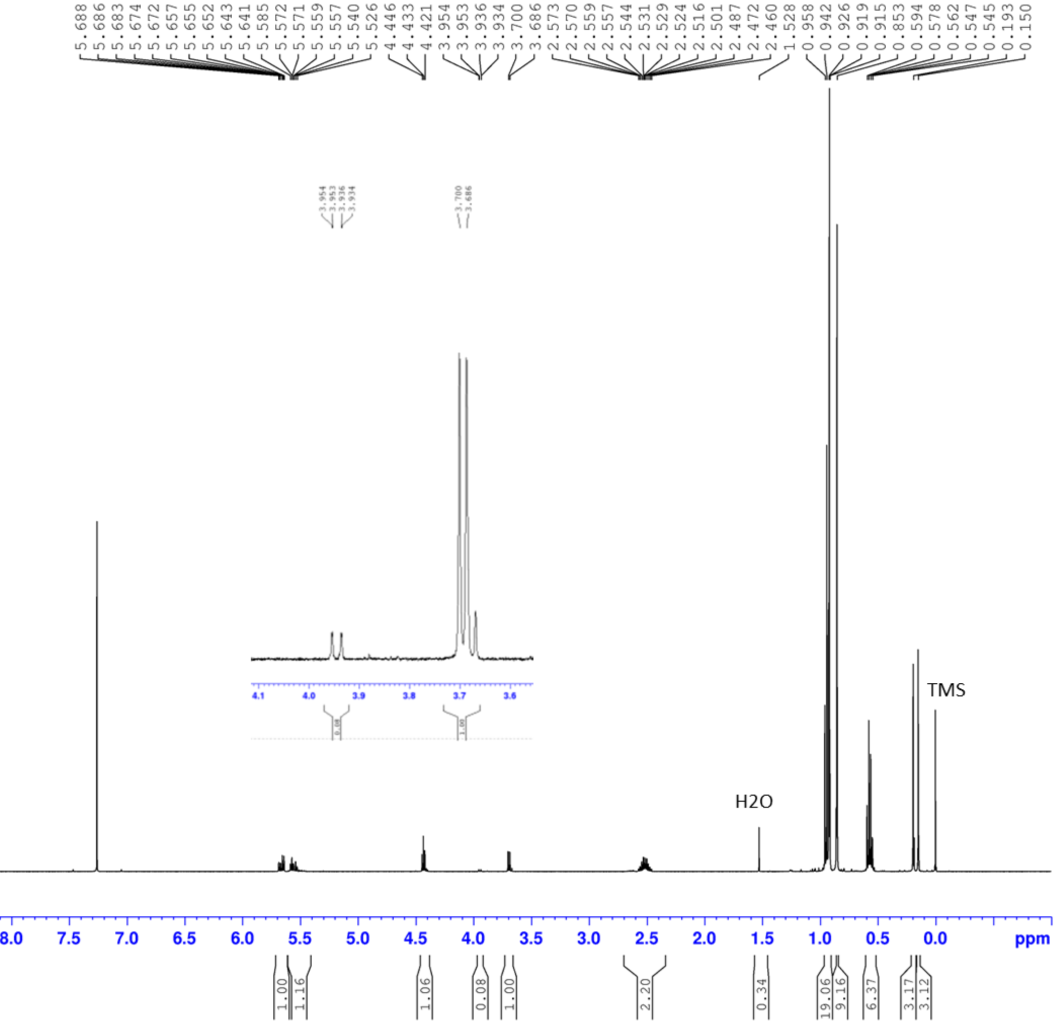


```
Current Data Parameters
NAME          JNH_03_165
EXPNO                  1
PROCNO                 1

F2 - Acquisition Parameters
Date_           20220829
Time               16.33 h
INSTRUM   Avance NEO 500
PROBHD   Z113652_0071 (
PULPROG             zg30
TD                 65536
SOLVENT            CDCl3
NS                    16
DS                     2
SWH            10000.000 Hz
FIDRES          0.305176 Hz
AQ             3.2767999 sec
RG                   101
DW                50.000 usec
DE                 10.45 usec
TE                 300.0 K
D1            1.00000000 sec
TD0                    1
SFO1         500.3030894 MHz
NUC1                  1H
P0                  4.00 usec
P1                 12.00 usec
PLW1         13.35999966 W

F2 - Processing parameters
SI                 65536
SF           500.3000123 MHz
WDW                   no
SSB                    0
LB                     0 Hz
GB                     0
PC                  1.00
```

^{13}C NMR ($CDCl_3$)

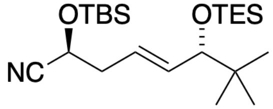

8

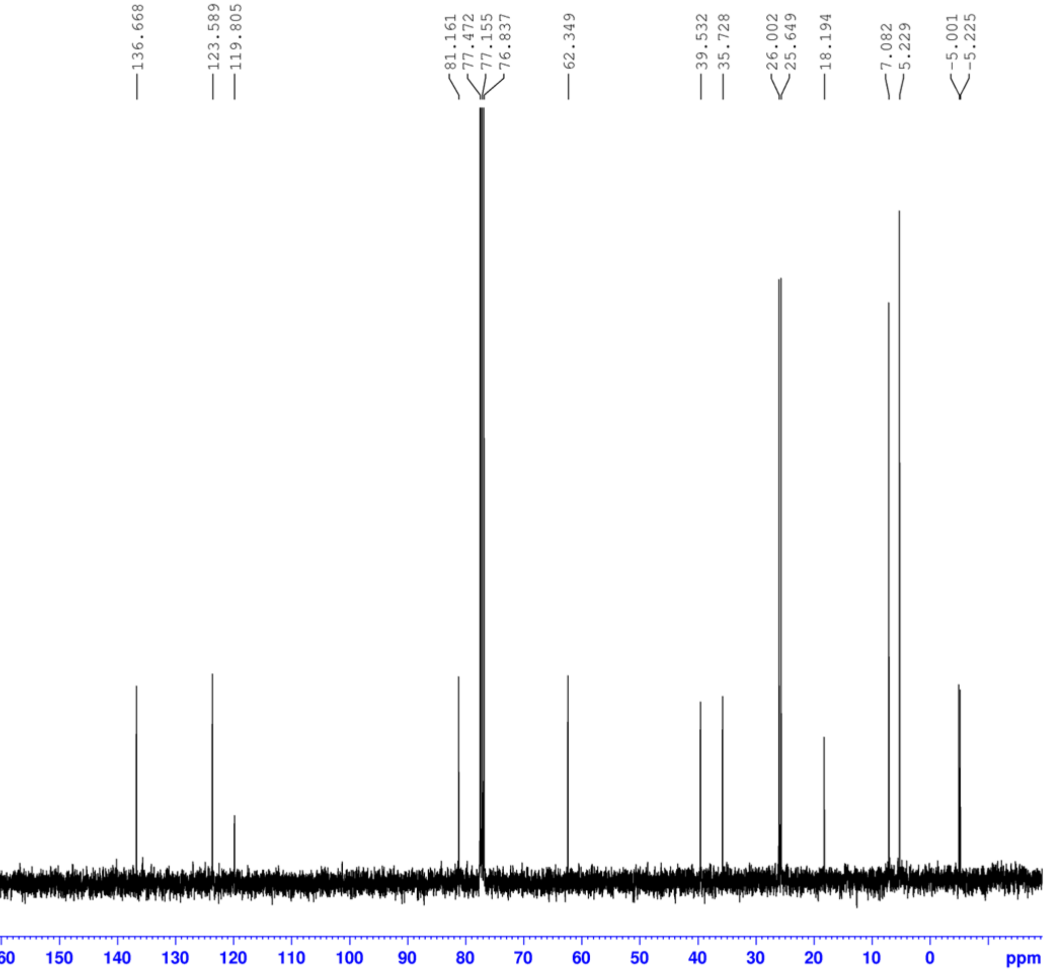

BRUKER

Current Data Parameters

NAME	JNH_02_289 C13
EXPNO	1
PROCNO	1

F2 - Acquisition Parameters

Date_	20220802
Time	11.38 h
INSTRUM	spect
PROBHD	Z104450_0192 (
PULPROG	zgpg30
TD	65536
SOLVENT	CDCl3
NS	100
DS	2
SWH	24038.461 Hz
FIDRES	0.733596 Hz
AQ	1.3631488 sec
RG	203
DW	20.800 usec
DE	6.50 usec
TE	295.9 K
D1	2.00000000 sec
D11	0.03000000 sec
TD0	1
SFO1	100.6228298 MHz
NUC1	13C
P0	3.28 usec
P1	9.85 usec
PLW1	28.63999939 W
SFO2	400.1316005 MHz
NUC2	1H
CPDPRG[2	waltz65
PCPD2	90.00 usec
PLW2	8.47000027 W
PLW12	0.23528001 W
PLW13	0.11834000 W

F2 - Processing parameters

SI	32768
SF	100.6127556 MHz
WDW	EM
SSB	0
LB	1.00 Hz
GB	0
PC	1.40

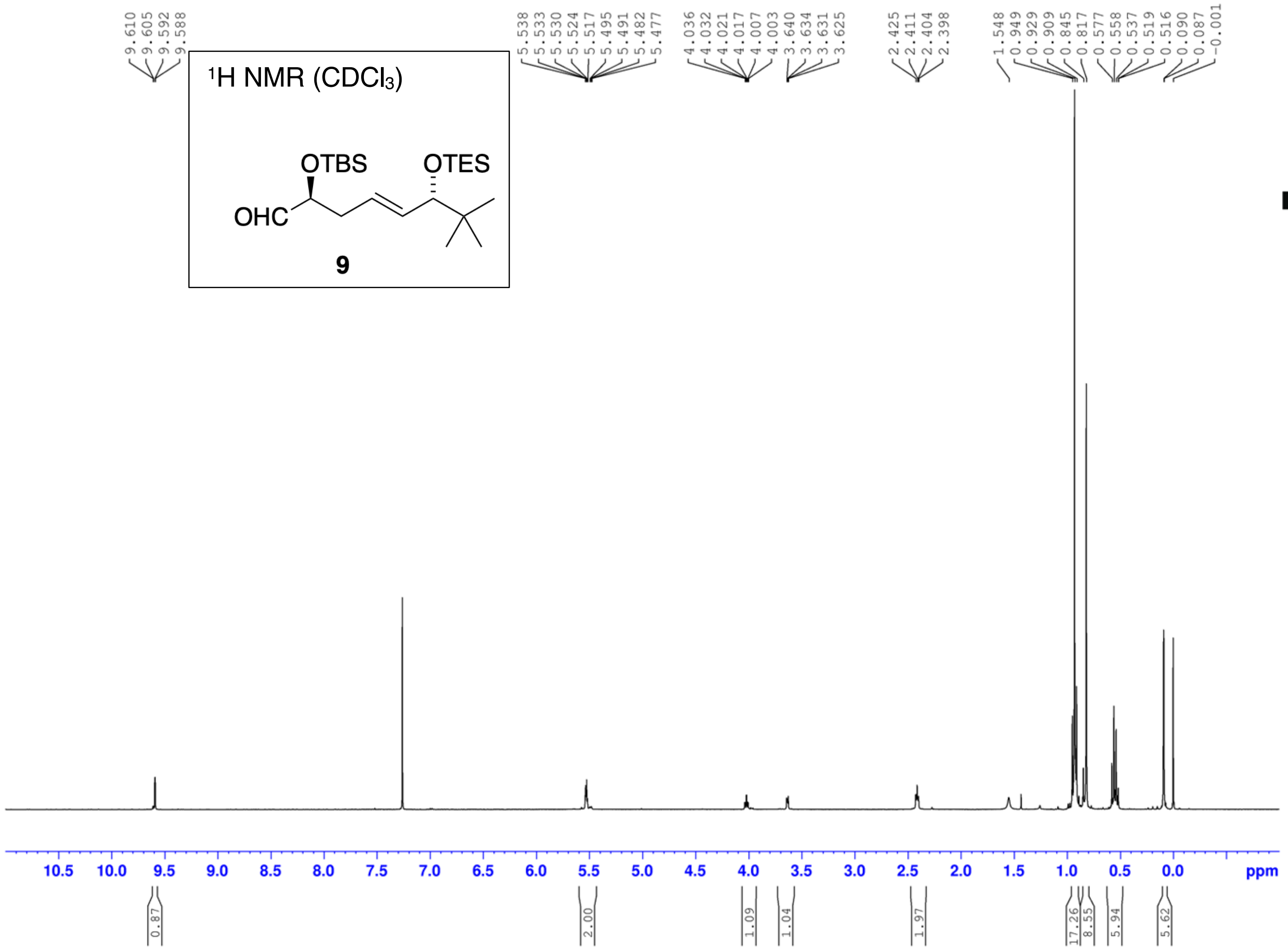


```
Current Data Parameters
NAME          JNH_03_222
EXPNO                  3
PROCNO                 1

F2 - Acquisition Parameters
Date_           20230110
Time             17.57 h
INSTRUM            spect
PROBHD   Z104450_0192 (
PULPROG             zg30
TD                 65536
SOLVENT            CDCl3
NS                    16
DS                     2
SWH             8012.820 Hz
FIDRES          0.244532 Hz
AQ             4.0894465 sec
RG                   575
DW                62.400 usec
DE                 16.92 usec
TE                 297.0 K
D1            1.00000000 sec
TD0                    1
SFO1         400.1324708 MHz
NUC1                  1H
P0                  5.00 usec
P1                 15.00 usec
PLW1          8.47000027 W

F2 - Processing parameters
SI                 65536
SF           400.1300096 MHz
WDW                   EM
SSB                    0
LB                  0.30 Hz
GB                     0
PC                  1.00
```

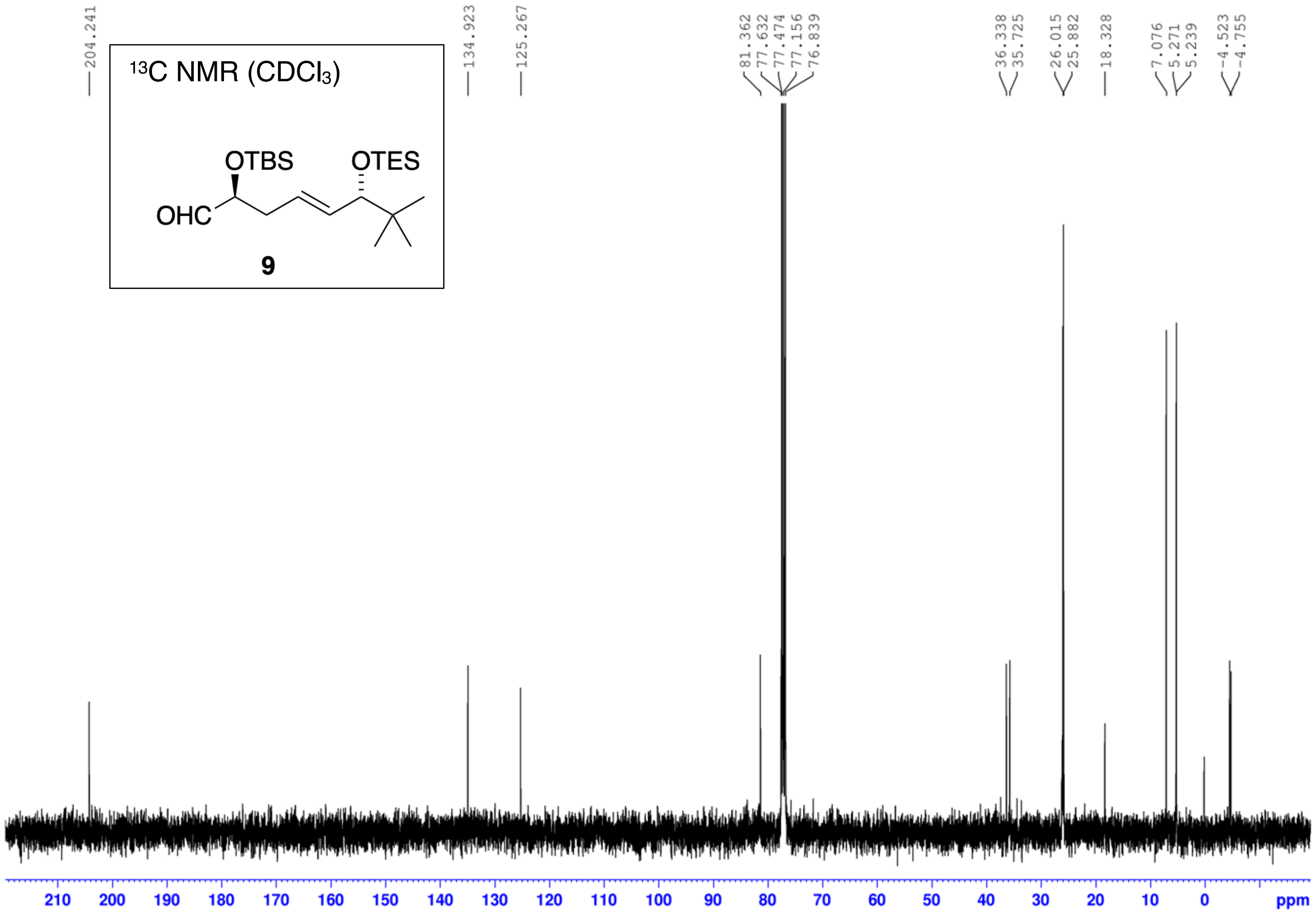

Current Data Parameters
NAME JNH_03_152 13C
EXPNO 1
PROCNO 1

F2 - Acquisition Parameters
Date_ 20220812
Time 19.08 h
INSTRUM spect
PROBHD Z104450_0192 (
PULPROG zgpg30
TD 65536
SOLVENT CDCl3
NS 981
DS 2
SWH 24038.461 Hz
FIDRES 0.733596 Hz
AQ 1.3631488 sec
RG 114
DW 20.800 usec
DE 6.50 usec
TE 295.4 K
D1 2.00000000 sec
D11 0.03000000 sec
TD0 1
SFO1 100.6228298 MHz
NUC1 13C
P0 3.28 usec
P1 9.85 usec
PLW1 28.63999939 W
SFO2 400.1316005 MHz
NUC2 1H
CPDPRG[2 waltz65
PCPD2 90.00 usec
PLW2 8.47000027 W
PLW12 0.23528001 W
PLW13 0.11834000 W

F2 - Processing parameters
SI 32768
SF 100.6127552 MHz
WDW EM
SSB 0
LB 1.00 Hz
GB 0
PC 1.40

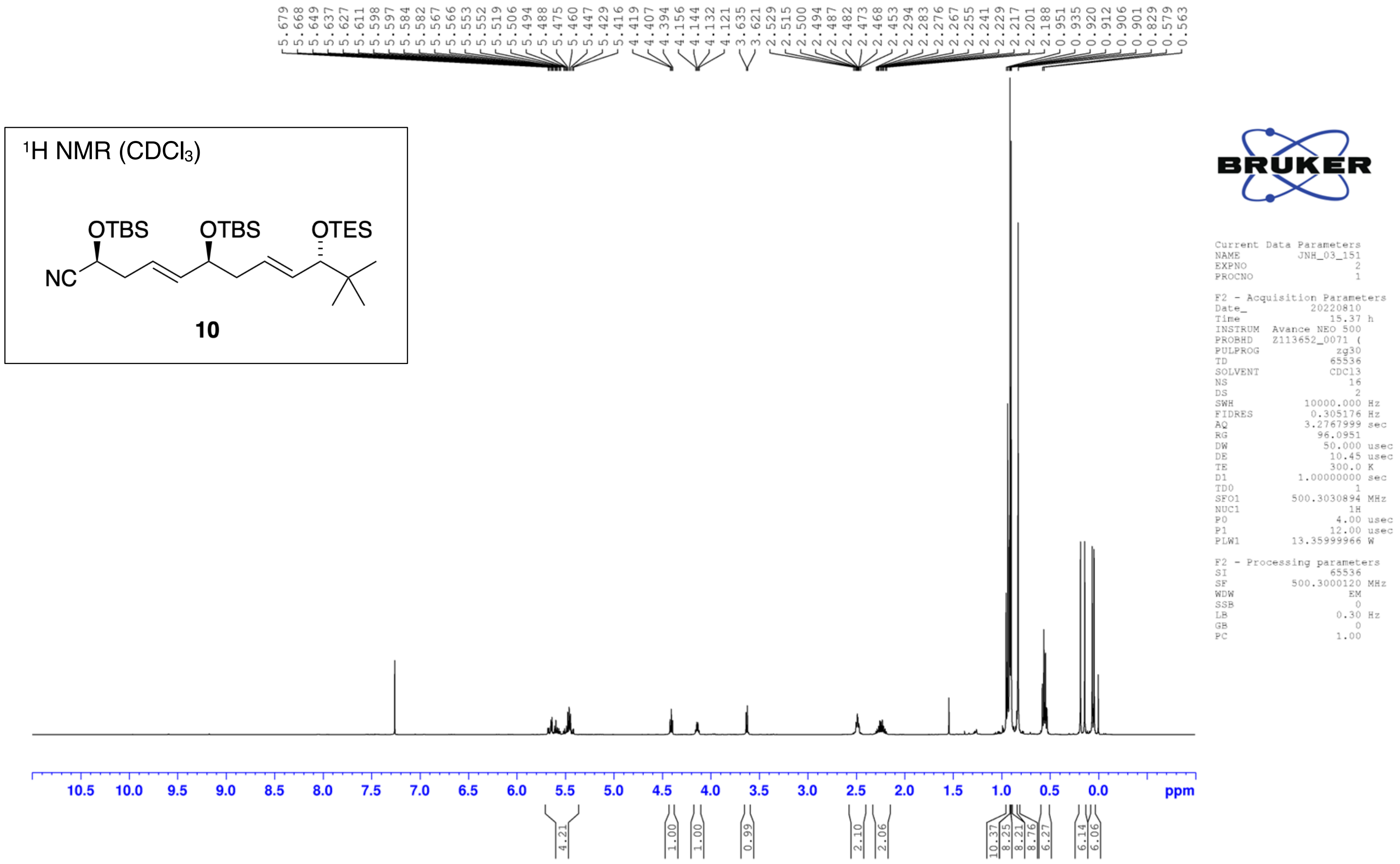

¹H NMR (CDCl₃)
OTBS
OTBS
OTES
NC
10
5.679 5.668 5.649 5.637 5.627 5.611 5.598 5.597 5.584 5.582 5.567 5.566 5.553 5.552 5.519 5.506 5.494 5.488 5.475 5.460 5.447 5.429 5.416 4.419 4.407 4.394 4.156 4.144 4.132 4.121 3.635 3.621 2.529 2.515 2.500 2.494 2.487 2.482 2.473 2.468 2.453 2.294 2.283 2.276 2.267 2.255 2.241 2.229 2.217 2.201 2.188 0.951 0.935 0.920 0.912 0.906 0.901 0.829 0.579 0.563
10.5 10.0 9.5 9.0 8.5 8.0 7.5 7.0 6.5 6.0 5.5 5.0 4.5 4.0 3.5 3.0 2.5 2.0 1.5 1.0 0.5 0.0 ppm
4.21 1.00 1.00 0.99 2.10 2.06 10.37 8.25 8.21 8.76 6.27 6.14 6.06
BRUKER
Current Data Parameters
NAME JNH_03_151
EXPNO 2
PROCNO 1
F2 - Acquisition Parameters
Date_ 20220810
Time 15.37 h
INSTRUM Avance NEO 500
PROBHD Z113652_0071 (
PULPROG zg30
TD 65536
SOLVENT CDCl3
NS 16
DS 2
SWH 10000.000 Hz
FIDRES 0.305176 Hz
AQ 3.2767999 sec
RG 96.0951
DW 50.000 usec
DE 10.45 usec
TE 300.0 K
D1 1.00000000 sec
TD0 1
SFO1 500.3030894 MHz
NUC1 1H
P0 4.00 usec
P1 12.00 usec
PLW1 13.35999966 W
F2 - Processing parameters
SI 65536
SF 500.3000120 MHz
WDW EM
SSB 0
LB 0.30 Hz
GB 0
PC 1.00

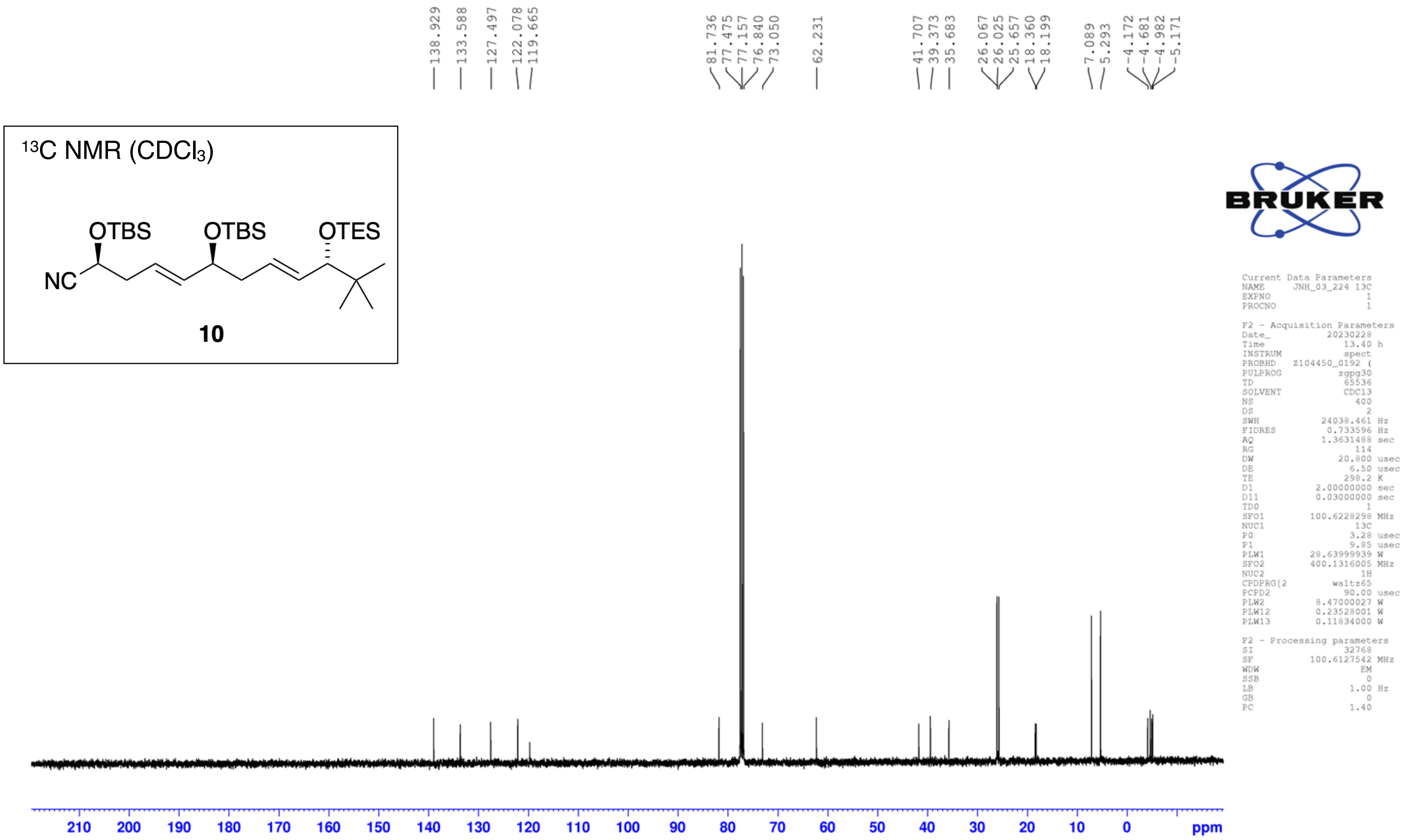

13C NMR (CDCl3)
OTBS
OTBS
OTES
NC
10
138.929
133.588
127.497
122.078
119.665
81.736
77.475
77.157
76.840
73.050
62.231
41.707
39.373
35.683
26.067
26.025
25.657
18.360
18.199
7.089
5.293
-4.172
-4.681
-4.982
-5.171
BRUKER
Current Data Parameters
NAME JNH_03_224 13C
EXPNO 1
PROCNO 1
F2 - Acquisition Parameters
Date_ 20230228
Time 13.40 h
INSTRUM spect
PROBHD Z104450_0192 (
PULPROG zgpg30
TD 65536
SOLVENT CDCl3
NS 400
DS 2
SWH 24038.461 Hz
FIDRES 0.733596 Hz
AQ 1.3631488 sec
RG 114
DW 20.800 usec
DE 6.50 usec
TE 298.2 K
D1 2.00000000 sec
D11 0.03000000 sec
TD0 1
SFO1 100.6228298 MHz
NUC1 13C
P0 3.28 usec
P1 9.85 usec
PLW1 28.63999939 W
SFO2 400.1316005 MHz
NUC2 1H
CPDPRG[2 waltz65
PCPD2 90.00 usec
PLW2 8.47000027 W
PLW12 0.23528001 W
PLW13 0.11834000 W
F2 - Processing parameters
SI 32768
SF 100.6127542 MHz
WDW EM
SSB 0
LB 1.00 Hz
GB 0
PC 1.40
210 200 190 180 170 160 150 140 130 120 110 100 90 80 70 60 50 40 30 20 10 0 ppm

^1H NMR ($CDCl_3$)

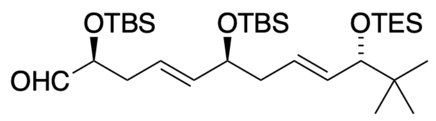

11

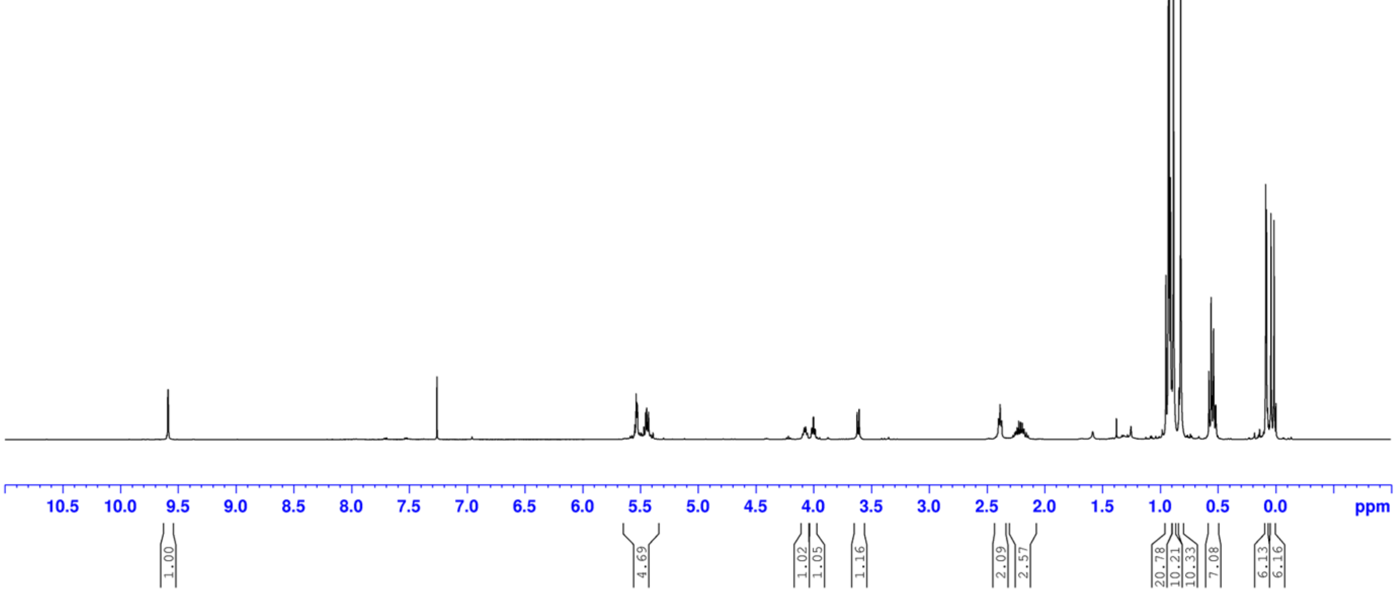


```
Current Data Parameters
NAME            JNH_03_154
EXPNO                    3
PROCNO                   1

F2 - Acquisition Parameters
Date_             20220816
Time                8.35 h
INSTRUM              spect
PROBHD   Z104450_0192 (
PULPROG               zg30
TD                   65536
SOLVENT              CDCl3
NS                      16
DS                       2
SWH               8012.820 Hz
FIDRES            0.244532 Hz
AQ               4.0894465 sec
RG                    80.6
DW                  62.400 usec
DE                   16.92 usec
TE                   294.5 K
D1              1.00000000 sec
TD0                      1
SFO1           400.1324708 MHz
NUC1                    1H
P0                    5.00 usec
P1                   15.00 usec
PLW1            8.47000027 W

F2 - Processing parameters
SI                   65536
SF             400.1300098 MHz
WDW                     EM
SSB                      0
LB                    0.30 Hz
GB                       0
PC                    1.00
```

^{13}C NMR ($CDCl_3$)

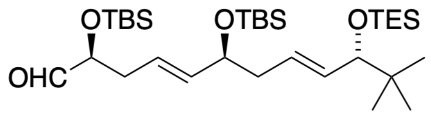

11

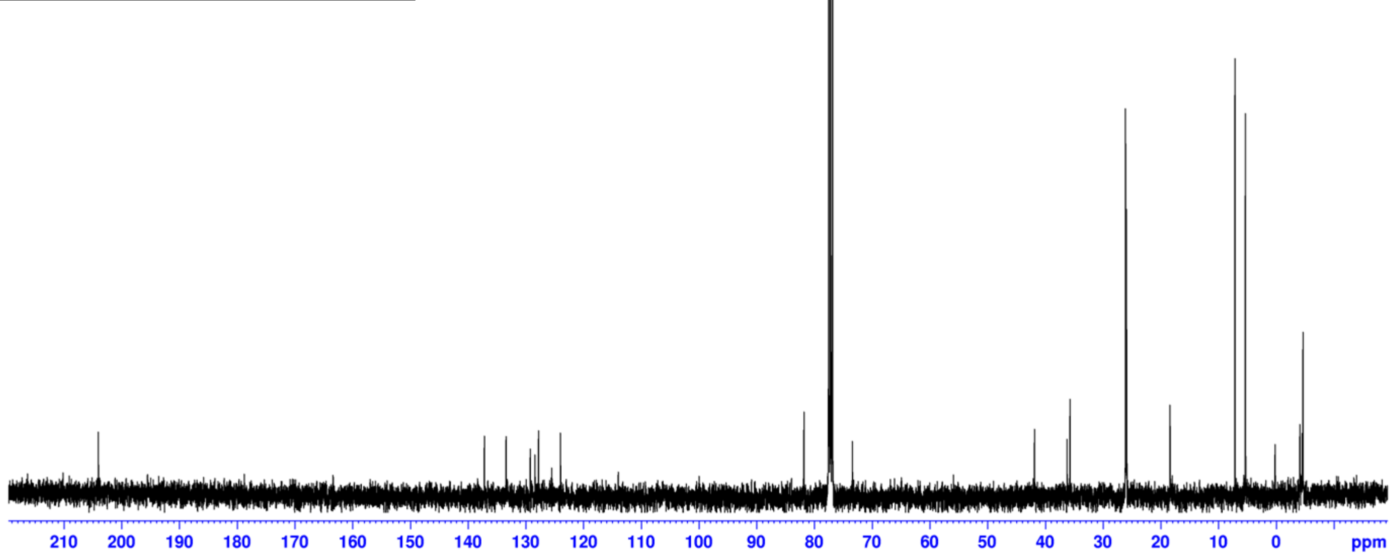


```
Current Data Parameters
NAME      JNH_03_154 13C
EXPNO                  1
PROCNO                 1

F2 - Acquisition Parameters
Date_           20220815
Time               15.26 h
INSTRUM            spect
PROBHD    Z104450_0192 (
PULPROG           zgpg30
TD                 65536
SOLVENT            CDCl3
NS                   550
DS                     2
SWH            24038.461 Hz
FIDRES          0.733596 Hz
AQ             1.3631488 sec
RG                   203
DW                20.800 usec
DE                  6.50 usec
TE                 295.5 K
D1            2.00000000 sec
D11           0.03000000 sec
TD0                    1
SFO1         100.6228298 MHz
NUC1                 13C
P0                  3.28 usec
P1                  9.85 usec
PLW1         28.63999939 W
SFO2         400.1316005 MHz
NUC2                  1H
CPDPRG[2          waltz65
PCPD2              90.00 usec
PLW2          8.47000027 W
PLW12         0.23528001 W
PLW13         0.11834000 W

F2 - Processing parameters
SI                 32768
SF           100.6127549 MHz
WDW                   EM
SSB                    0
LB                  1.00 Hz
GB                     0
PC                  1.40
```

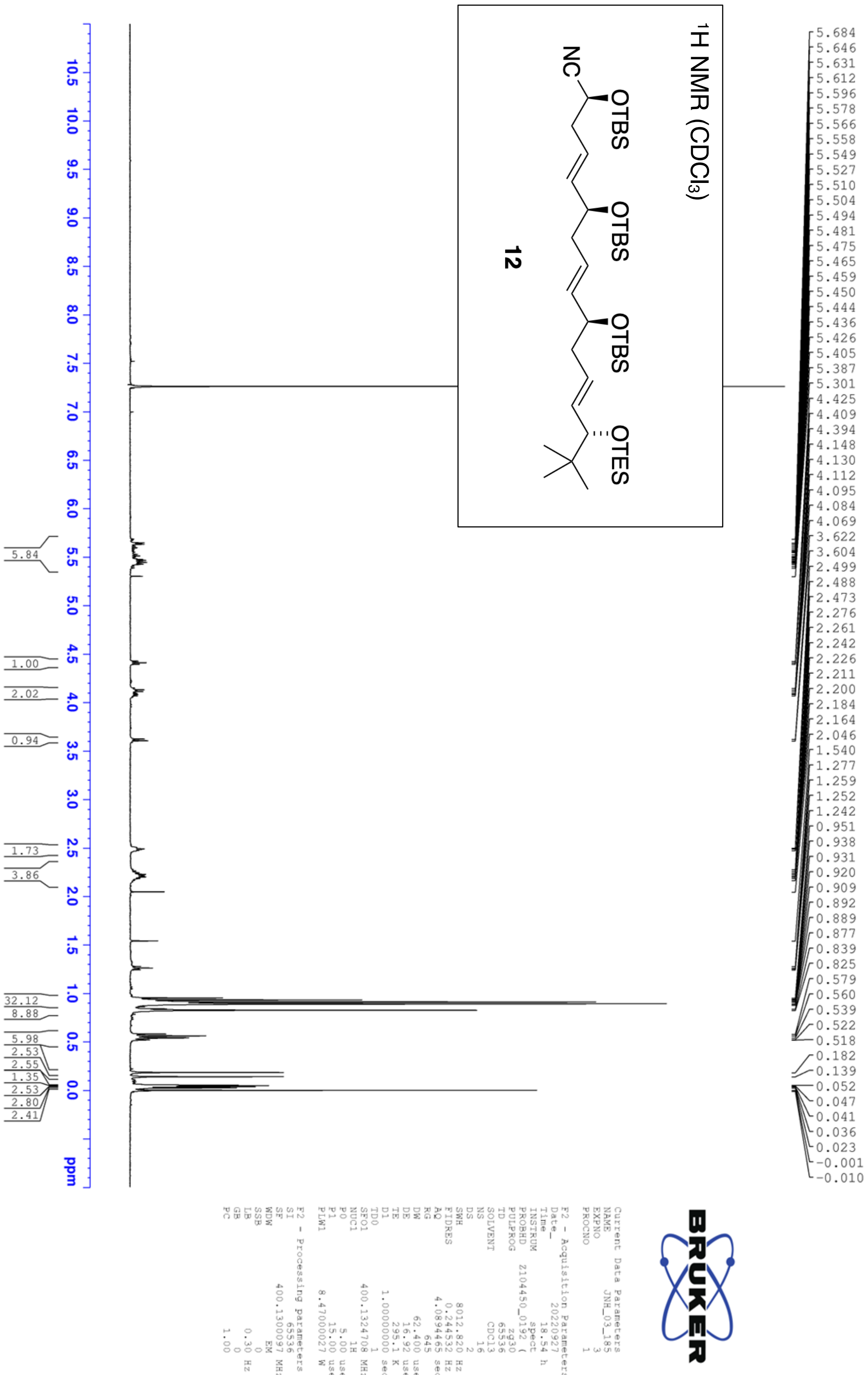
1H NMR (CDCl3)
NC
OTBS
OTBS
OTBS
OTES
12
5.684 5.646 5.631 5.612 5.596 5.578 5.566 5.558 5.549 5.527 5.510 5.504 5.494 5.481 5.475 5.465 5.459 5.450 5.444 5.436 5.426 5.405 5.387 5.301 4.425 4.409 4.394 4.148 4.130 4.112 4.095 4.084 4.069 3.622 3.604 2.499 2.488 2.473 2.276 2.261 2.242 2.226 2.211 2.200 2.184 2.164 2.046 1.540 1.277 1.259 1.252 1.242 0.951 0.938 0.931 0.920 0.909 0.892 0.889 0.877 0.839 0.825 0.579 0.560 0.539 0.522 0.518 0.182 0.139 0.052 0.047 0.041 0.036 0.023 -0.001 -0.010
5.84
1.00
2.02
0.94
1.73
3.86
32.12
8.88
5.98
2.53
2.55
1.35
2.53
2.80
2.41
ppm

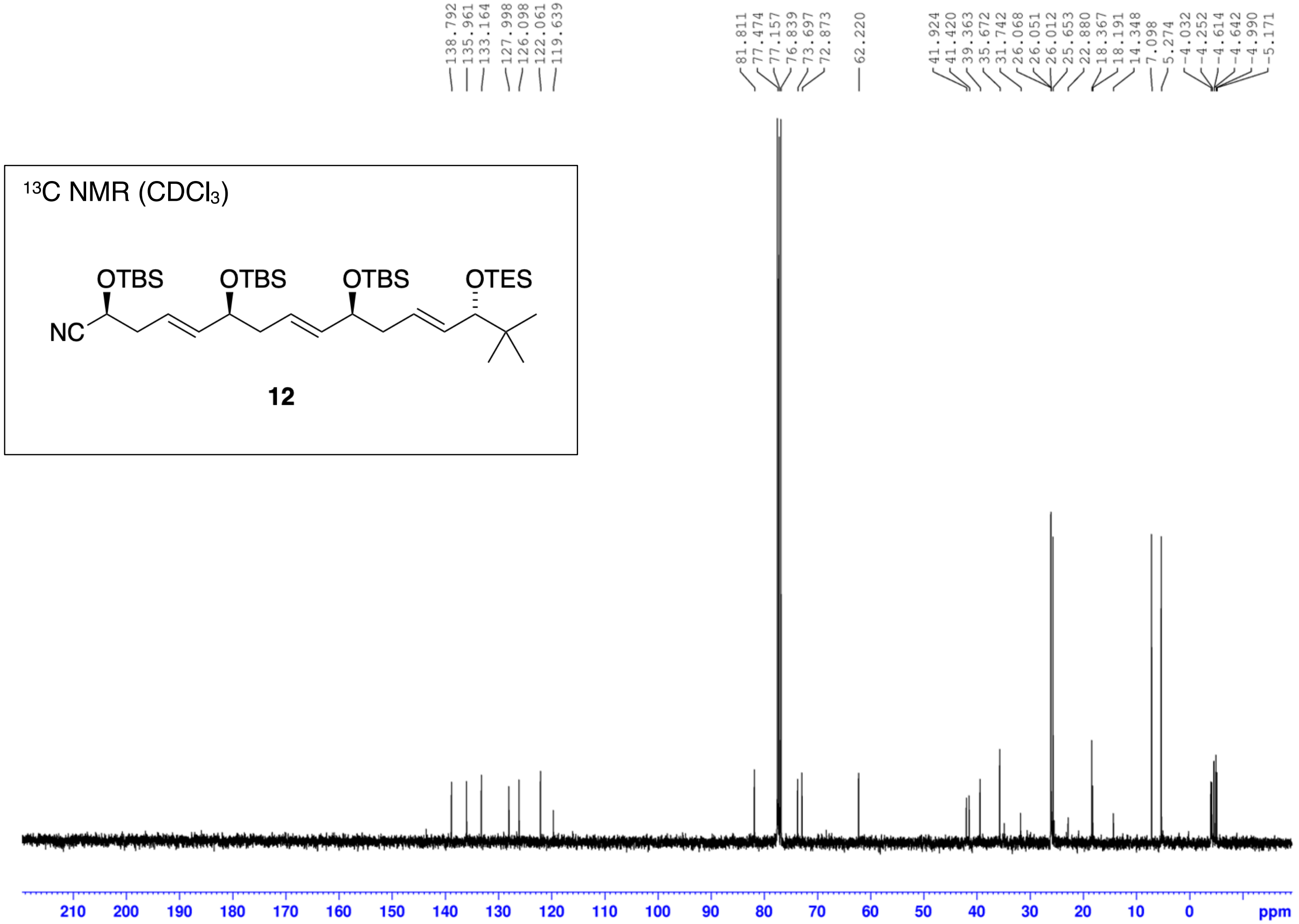


```
Current Data Parameters
NAME       JNH_03_156 13C
EXPNO                   1
PROCNO                  1

F2 - Acquisition Parameters
Date_            20220816
Time               16.42 h
INSTRUM             spect
PROBHD     Z104450_0192 (
PULPROG            zgpg30
TD                  65536
SOLVENT             CDCl3
NS                    254
DS                      2
SWH             24038.461 Hz
FIDRES           0.733596 Hz
AQ              1.3631488 sec
RG                    203
DW                 20.800 usec
DE                   6.50 usec
TE                  295.1 K
D1             2.00000000 sec
D11            0.03000000 sec
TD0                     1
SFO1          100.6228298 MHz
NUC1                  13C
P0                   3.28 usec
P1                   9.85 usec
PLW1          28.63999939 W
SFO2          400.1316005 MHz
NUC2                   1H
CPDPRG[2          waltz65
PCPD2               90.00 usec
PLW2           8.47000027 W
PLW12          0.23528001 W
PLW13          0.11834000 W

F2 - Processing parameters
SI                  32768
SF            100.6127554 MHz
WDW                    EM
SSB                     0
LB                   1.00 Hz
GB                      0
PC                   1.40
```

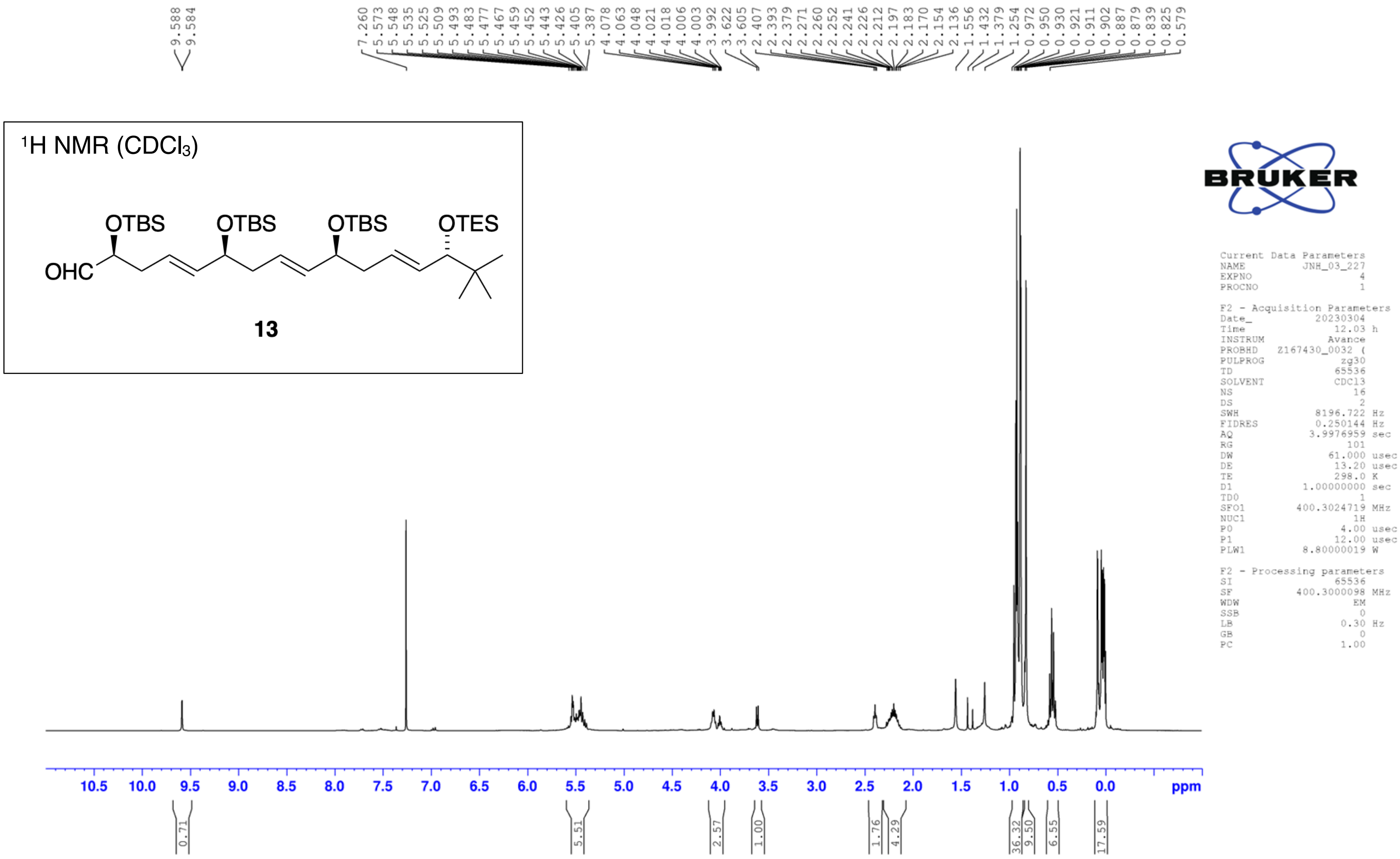

1H NMR (CDCl3)
OTBS
OTBS
OTBS
OTES
OHC
13
9.588
9.584
7.260
5.573
5.548
5.535
5.525
5.509
5.493
5.483
5.477
5.467
5.459
5.452
5.443
5.426
5.405
5.387
4.078
4.063
4.048
4.021
4.018
4.006
4.003
3.992
3.622
3.605
2.407
2.393
2.379
2.271
2.260
2.252
2.241
2.226
2.212
2.197
2.183
2.170
2.154
2.136
1.556
1.432
1.379
1.254
0.972
0.950
0.930
0.921
0.911
0.902
0.887
0.879
0.839
0.825
0.579
0.71
5.51
2.57
1.00
1.76
4.29
36.32
9.50
6.55
17.59
10.5 10.0 9.5 9.0 8.5 8.0 7.5 7.0 6.5 6.0 5.5 5.0 4.5 4.0 3.5 3.0 2.5 2.0 1.5 1.0 0.5 0.0 ppm
BRUKER
Current Data Parameters
NAME JNH_03_227
EXPNO 4
PROCNO 1
F2 - Acquisition Parameters
Date_ 20230304
Time 12.03 h
INSTRUM Avance
PROBHD Z167430_0032 (
PULPROG zg30
TD 65536
SOLVENT CDCl3
NS 16
DS 2
SWH 8196.722 Hz
FIDRES 0.250144 Hz
AQ 3.9976959 sec
RG 101
DW 61.000 usec
DE 13.20 usec
TE 298.0 K
D1 1.00000000 sec
TD0 1
SFO1 400.3024719 MHz
NUC1 1H
P0 4.00 usec
P1 12.00 usec
PLW1 8.80000019 W
F2 - Processing parameters
SI 65536
SF 400.3000098 MHz
WDW EM
SSB 0
LB 0.30 Hz
GB 0
PC 1.00

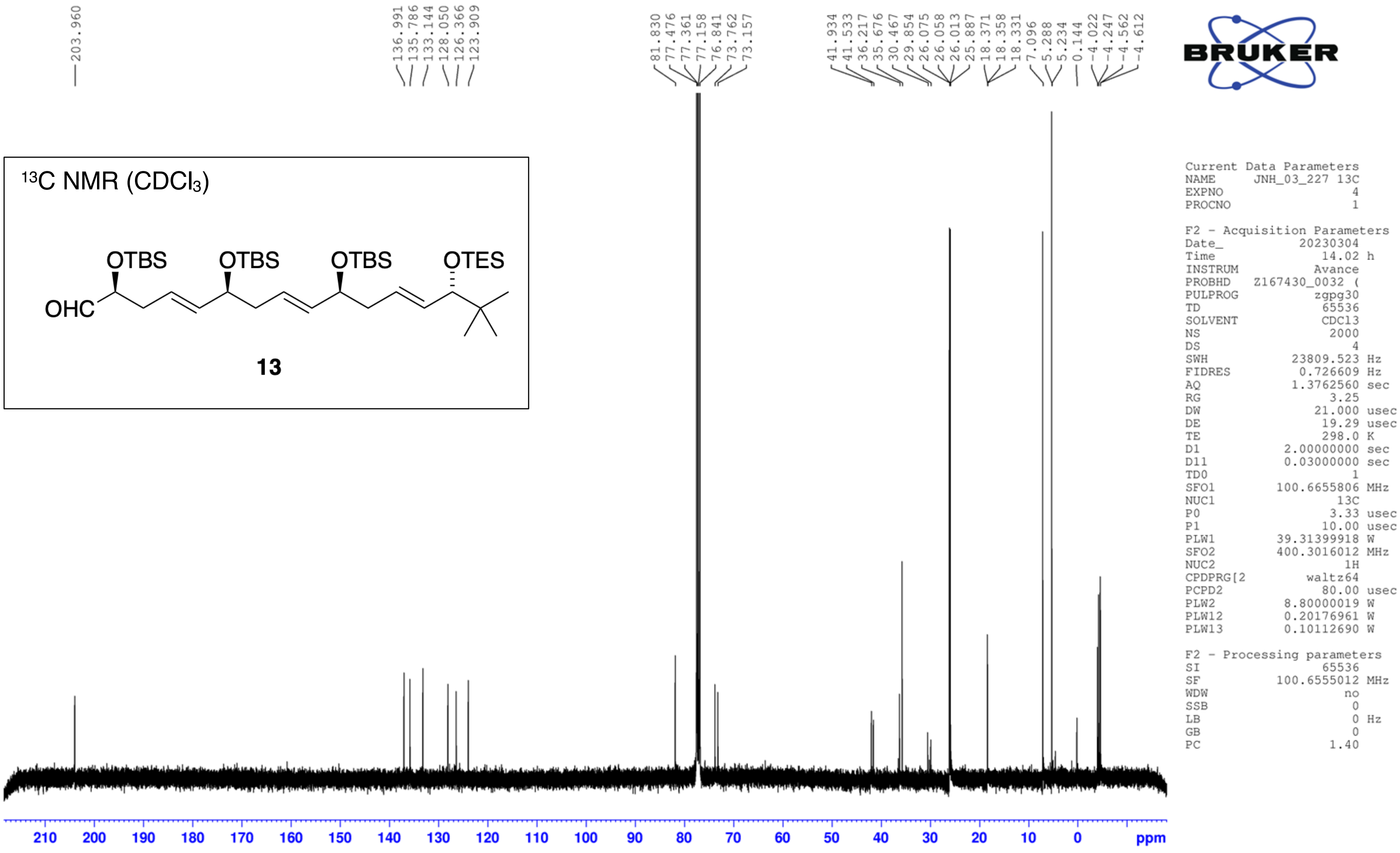

203.960
136.991
135.786
133.144
128.050
126.366
123.909
81.830
77.476
77.361
77.158
76.841
73.762
73.157
41.934
41.533
36.217
35.676
30.467
29.854
26.075
26.058
26.013
25.887
18.371
18.358
18.331
7.096
5.288
5.234
0.144
-4.022
-4.247
-4.562
-4.612
^{13}C NMR ($CDCl_3$)
OTBS
OTBS
OTBS
OTES
OHC
13
210 200 190 180 170 160 150 140 130 120 110 100 90 80 70 60 50 40 30 20 10 0 ppm
BRUKER
Current Data Parameters
NAME JNH_03_227 13C
EXPNO 4
PROCNO 1
F2 - Acquisition Parameters
Date_ 20230304
Time 14.02 h
INSTRUM Avance
PROBHD Z167430_0032 (
PULPROG zgpg30
TD 65536
SOLVENT CDCl3
NS 2000
DS 4
SWH 23809.523 Hz
FIDRES 0.726609 Hz
AQ 1.3762560 sec
RG 3.25
DW 21.000 usec
DE 19.29 usec
TE 298.0 K
D1 2.00000000 sec
D11 0.03000000 sec
TD0 1
SFO1 100.6655806 MHz
NUC1 13C
P0 3.33 usec
P1 10.00 usec
PLW1 39.31399918 W
SFO2 400.3016012 MHz
NUC2 1H
CPDPRG[2 waltz64
PCPD2 80.00 usec
PLW2 8.80000019 W
PLW12 0.20176961 W
PLW13 0.10112690 W
F2 - Processing parameters
SI 65536
SF 100.6555012 MHz
WDW no
SSB 0
LB 0 Hz
GB 0
PC 1.40

¹H NMR ($CDCl_3$)

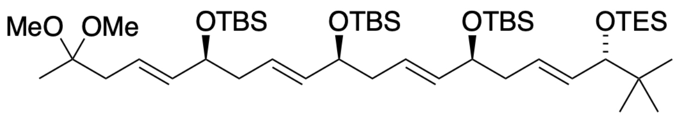

15

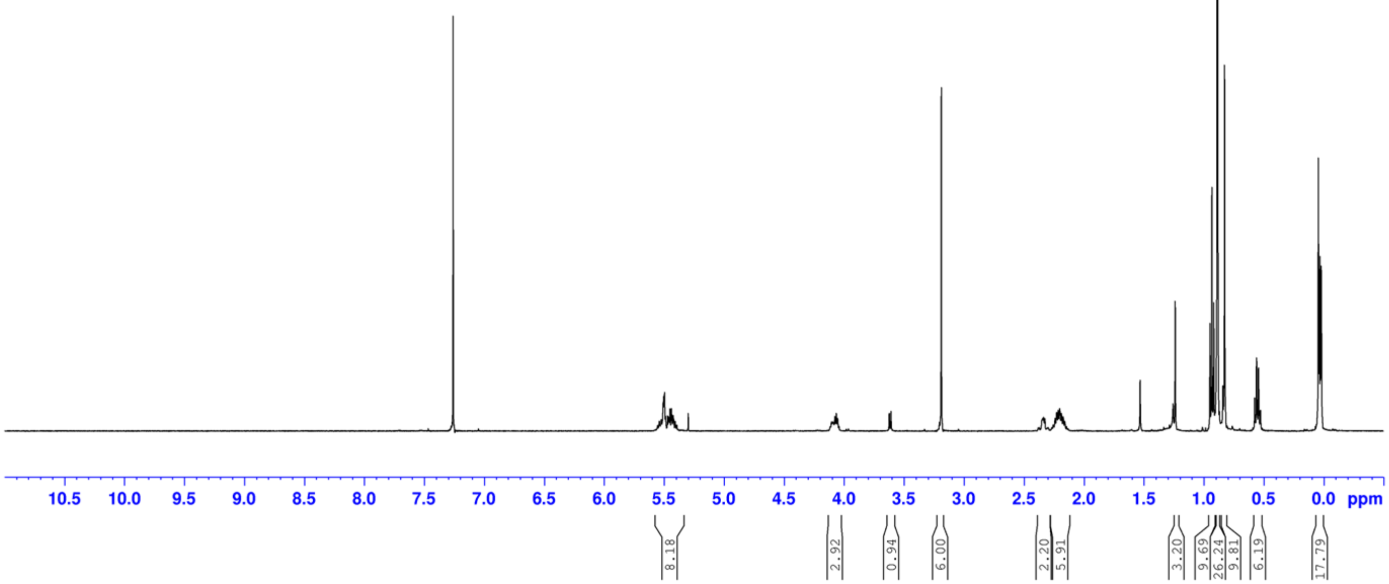


```
Current Data Parameters
NAME           JNH_03_230
EXPNO                   3
PROCNO                  1

F2 - Acquisition Parameters
Date_            20230313
Time                15.09 h
INSTRUM  Avance NEO 500
PROBHD    Z113652_0071 (
PULPROG              zg30
TD                  65536
SOLVENT             CDCl3
NS                     16
DS                      2
SWH             10000.000 Hz
FIDRES           0.305176 Hz
AQ              3.2767999 sec
RG                    101
DW                 50.000 usec
DE                  10.45 usec
TE                  300.0 K
D1             1.00000000 sec
TD0                     1
SFO1          500.3030894 MHz
NUC1                   1H
P0                   4.00 usec
P1                  12.00 usec
PLW1          13.35999966 W

F2 - Processing parameters
SI                  65536
SF            500.3000121 MHz
WDW                    no
SSB                     0
LB                      0 Hz
GB                      0
PC                   1.00
```

^{13}C NMR ($CDCl_3$)

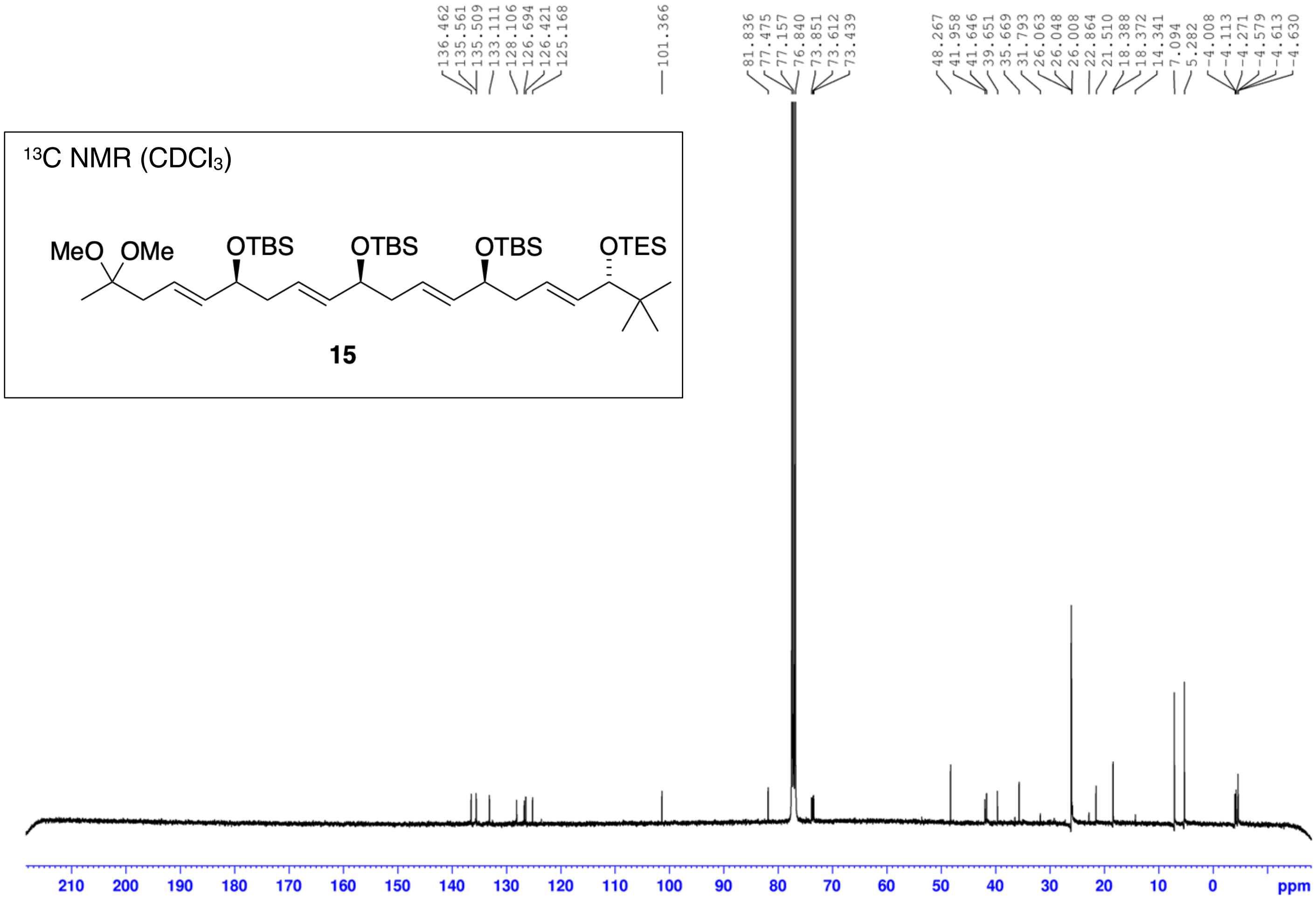

15

```
Current Data Parameters
NAME      JNH_03_228 13C
EXPNO               12
PROCNO               1

F2 - Acquisition Parameters
Date_         20230309
Time              1.13 h
INSTRUM         Avance
PROBHD   Z167430_0032 (
PULPROG         zgpg30
TD               65536
SOLVENT          CDCl3
NS                2390
DS                   4
SWH          23809.523 Hz
FIDRES        0.726609 Hz
AQ           1.3762560 sec
RG                3.25
DW              21.000 usec
DE               19.29 usec
TE               298.0 K
D1          2.00000000 sec
D11         0.03000000 sec
TD0                  1
SFO1       100.6655806 MHz
NUC1               13C
P0                3.33 usec
P1               10.00 usec
PLW1       39.31399918 W
SFO2       400.3016012 MHz
NUC2                1H
CPDPRG[2       waltz64
PCPD2            80.00 usec
PLW2        8.80000019 W
PLW12       0.20176961 W
PLW13       0.10112690 W

F2 - Processing parameters
SI               65536
SF         100.6555015 MHz
WDW                 EM
SSB                  0
LB                1.00 Hz
GB                   0
PC                1.40
```

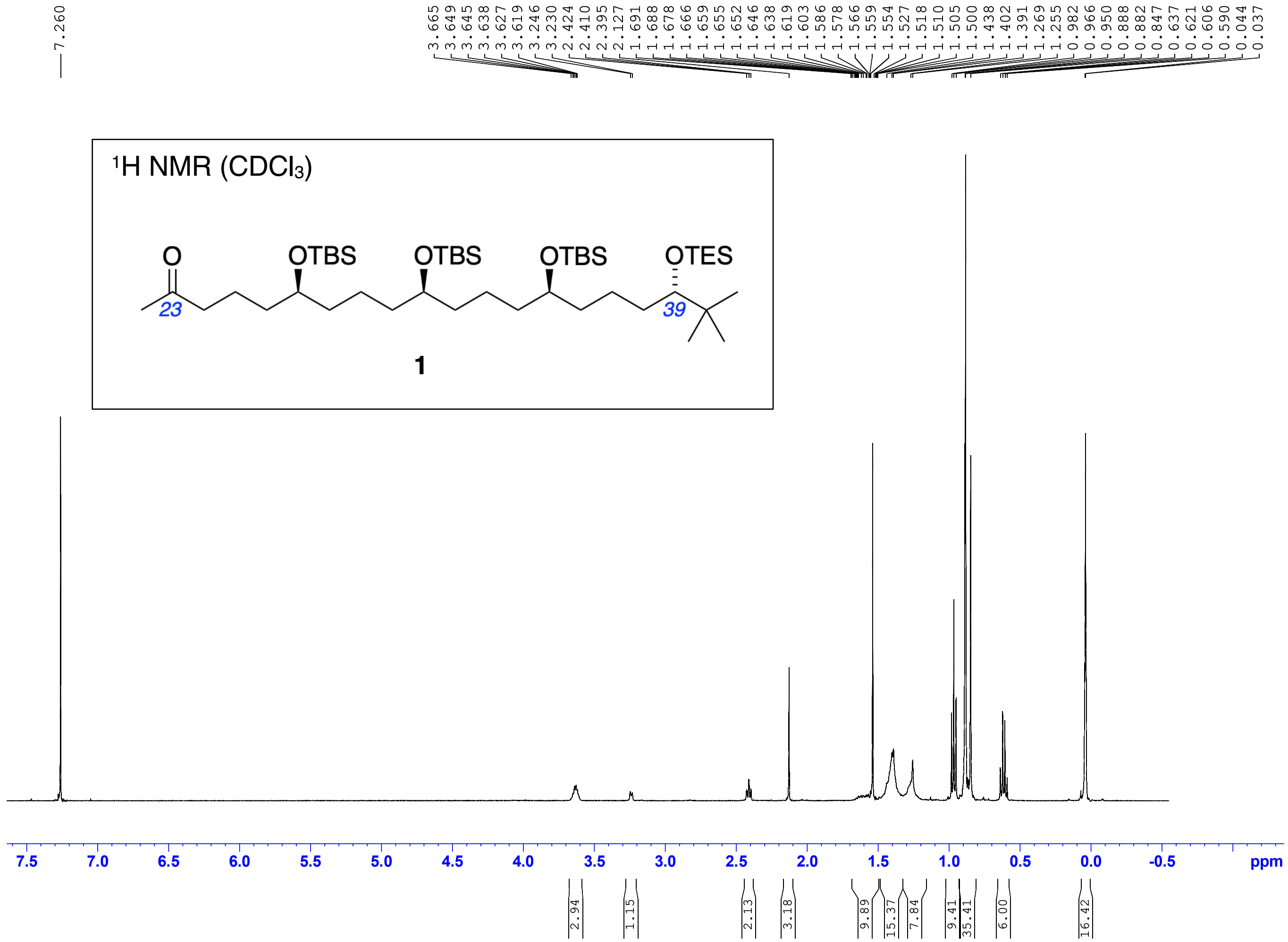


```
Current Data Parameters
NAME              GKF-1-286
EXPNO                    25
PROCNO                    1

F2 - Acquisition Parameters
Date_             20240313
Time                  6.51 h
INSTRUM     Avance NEO 500
PROBHD      Z113652_0071 (
PULPROG               zg30
TD                   65536
SOLVENT              CDCl3
NS                      33
DS                       2
SWH              5882.353 Hz
FIDRES           0.179515 Hz
AQ              5.5705600 sec
RG                     101
DW                  85.000 usec
DE                    8.58 usec
TE                   300.0 K
D1              1.00000000 sec
TD0                      1
SFO1           500.3026787 MHz
NUC1                    1H
P0                    4.00 usec
P1                   12.00 usec
PLW1           15.19999981 W

F2 - Processing parameters
SI                   65536
SF             500.3000121 MHz
WDW                     no
SSB                      0
LB                       0 Hz
GB                       0
PC                    1.00
```

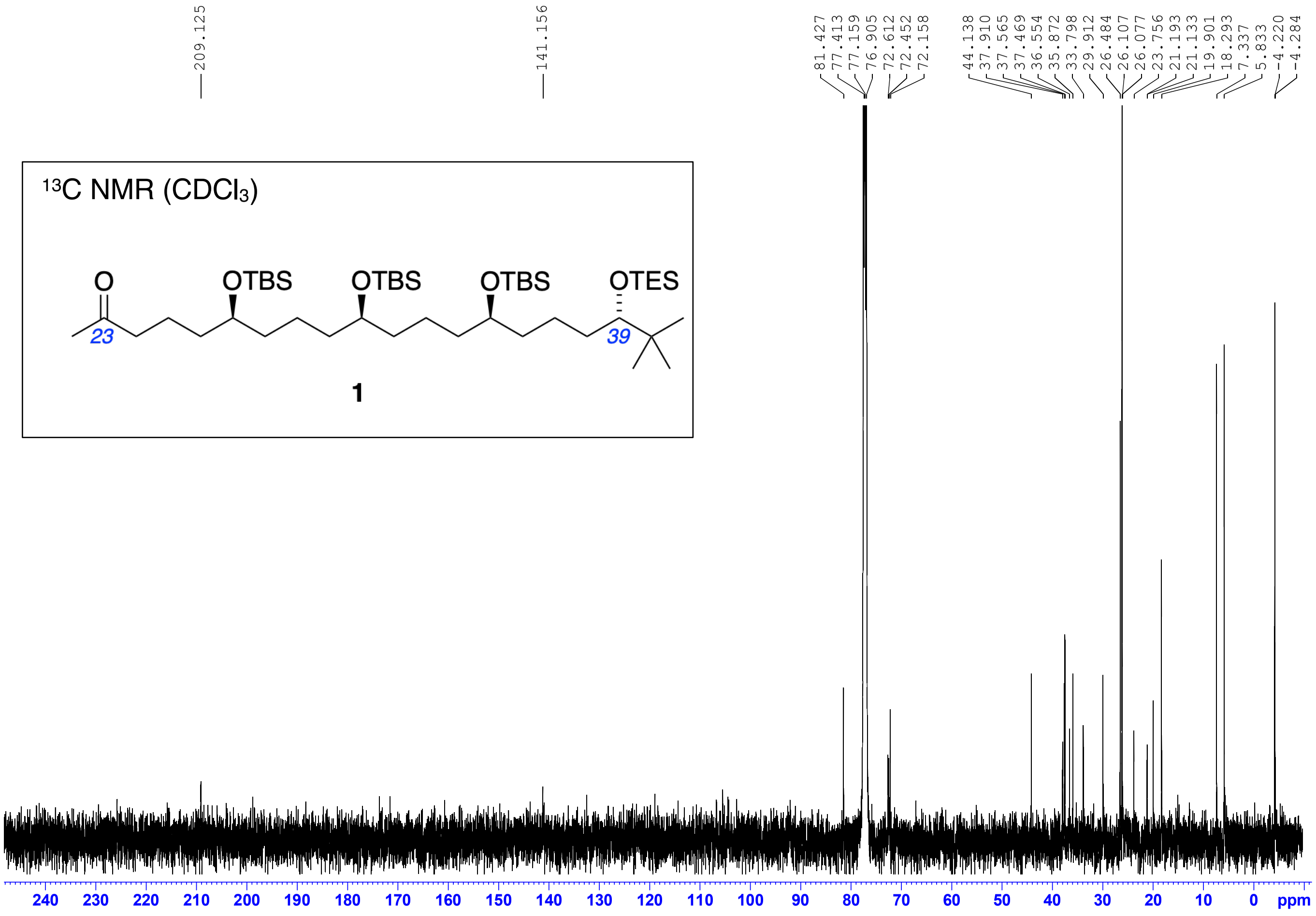

Current Data Parameters

NAME	13
EXPNO	13
PROCNO	1

F2 - Acquisition Parameters

Date_	20240314
Time	8.10 h
INSTRUM	Avance NEO 500
PROBHD	Z113652_0071 (
PULPROG	zgpg30
TD	65536
SOLVENT	CDCl3
NS	4096
DS	8
SWH	32679.739 Hz
FIDRES	0.997306 Hz
AQ	1.0027008 sec
RG	101
DW	15.300 usec
DE	10.00 usec
TE	300.0 K
D1	5.00000000 sec
D11	0.03000000 sec
TD0	1
SFO1	125.8156311 MHz
NUC1	13C
P0	3.25 usec
P1	9.75 usec
PLW1	104.00000000 W
SFO2	500.3020012 MHz
NUC2	1H
CPDPRG[2	waltz65
PCPD2	80.00 usec
PLW2	16.89999962 W
PLW12	0.38000000 W
PLW13	0.18783000 W

F2 - Processing parameters

SI	131072
SF	125.8005153 MHz
WDW	EM
SSB	0
LB	1.50 Hz
GB	0
PC	1.40

^1H NMR ($CDCl_3$)

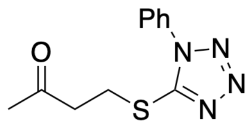

S3

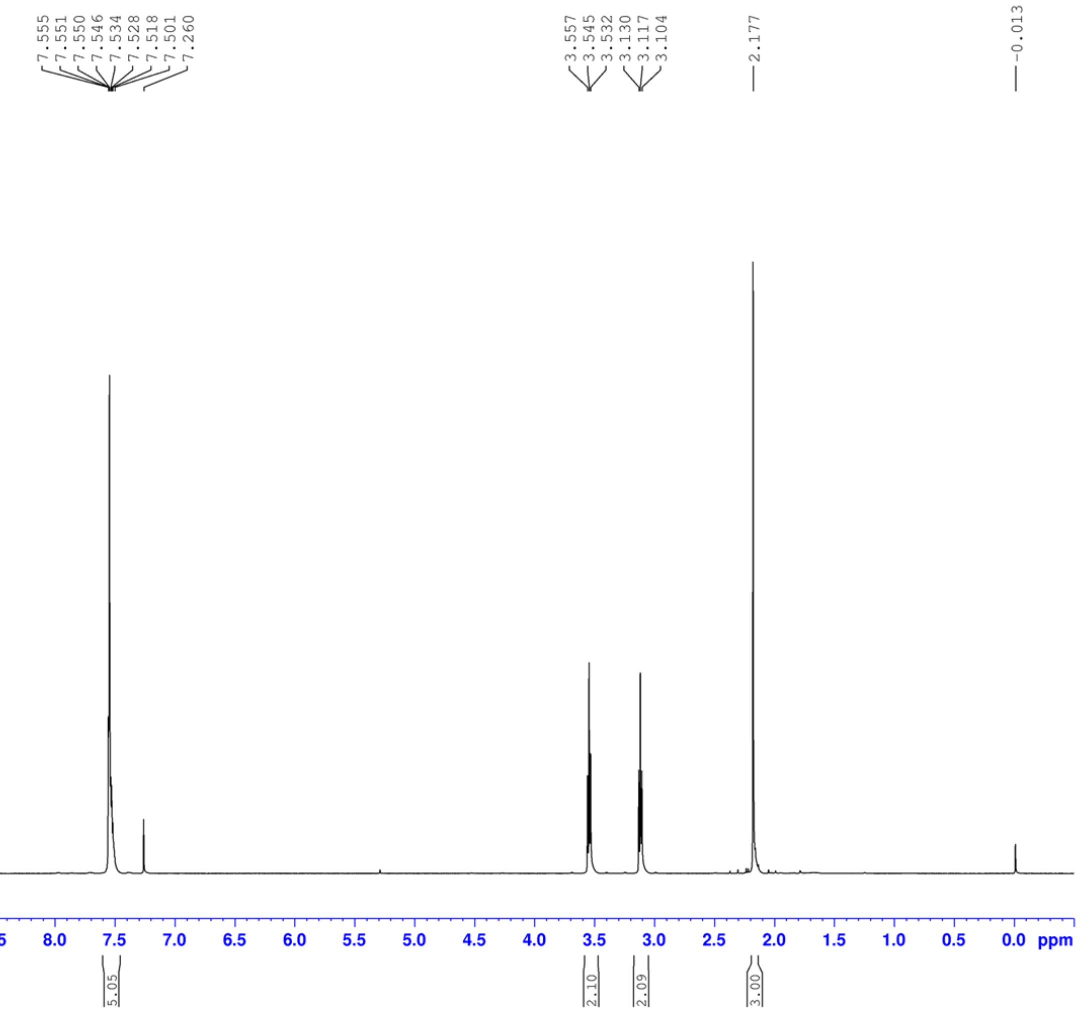


```
Current Data Parameters
NAME          JNH_03_161
EXPNO                  1
PROCNO                 1

F2 - Acquisition Parameters
Date_           20220823
Time               10.02 h
INSTRUM   Avance NEO 500
PROBHD    Z113652_0071 (
PULPROG             zg30
TD                 65536
SOLVENT            CDCl3
NS                    16
DS                     2
SWH            10000.000 Hz
FIDRES          0.305176 Hz
AQ             3.2767999 sec
RG                   101
DW                50.000 usec
DE                 10.45 usec
TE                 300.0 K
D1          1.00000000 sec
TD0                    1
SFO1         500.3030894 MHz
NUC1                  1H
P0                  4.00 usec
P1                 12.00 usec
PLW1         13.35999966 W

F2 - Processing parameters
SI                 65536
SF           500.3000121 MHz
WDW                   EM
SSB                    0
LB                  0.30 Hz
GB                     0
PC                  1.00
```

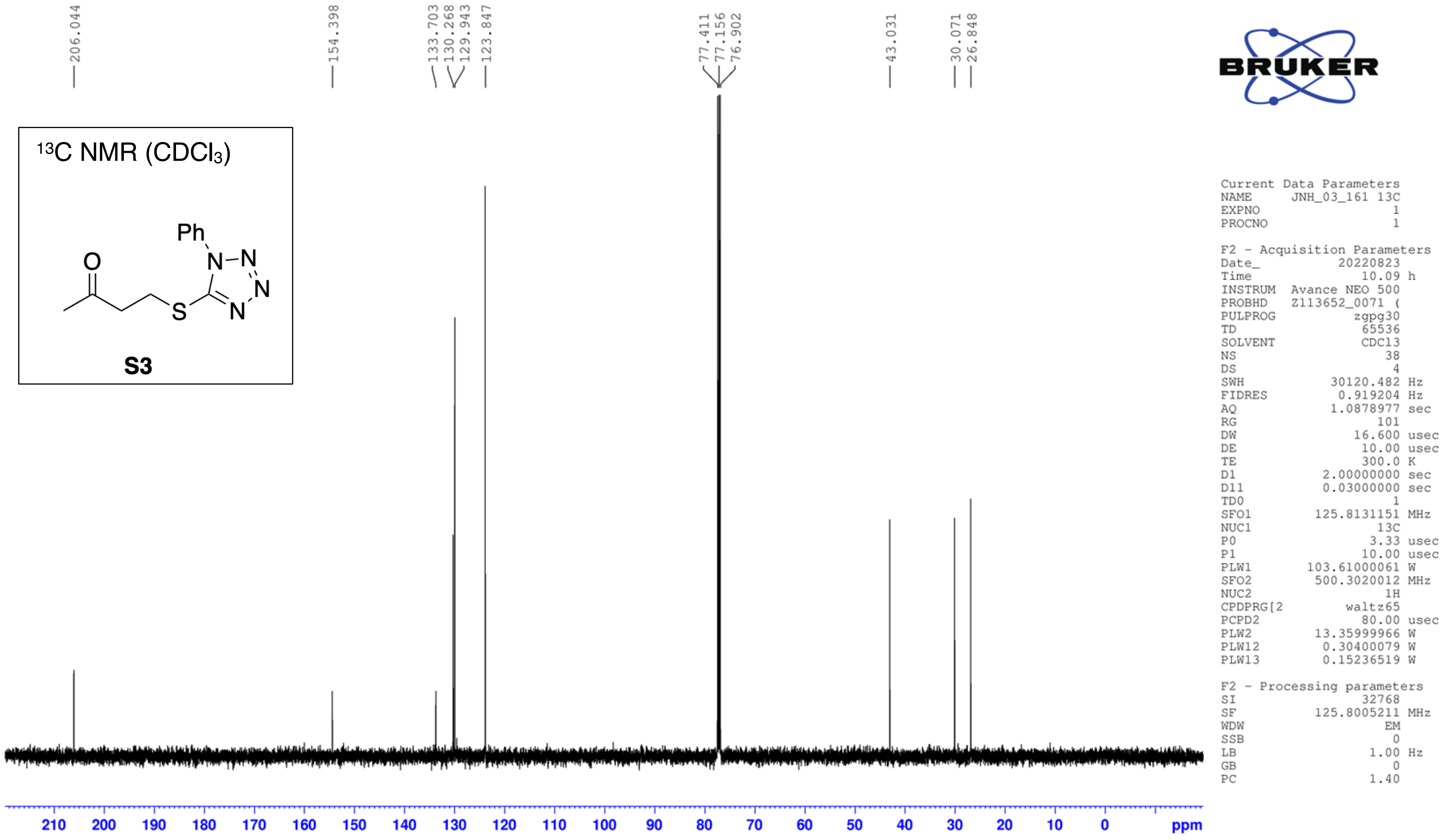

206.044
154.398
133.703
130.268
129.943
123.847
77.411
77.156
76.902
43.031
30.071
26.848
13C NMR (CDCl3)
Ph
N
N
N
N
O
S
S3
BRUKER
Current Data Parameters
NAME JNH_03_161 13C
EXPNO 1
PROCNO 1
F2 - Acquisition Parameters
Date_ 20220823
Time 10.09 h
INSTRUM Avance NEO 500
PROBHD Z113652_0071 (
PULPROG zgpg30
TD 65536
SOLVENT CDCl3
NS 38
DS 4
SWH 30120.482 Hz
FIDRES 0.919204 Hz
AQ 1.0878977 sec
RG 101
DW 16.600 usec
DE 10.00 usec
TE 300.0 K
D1 2.00000000 sec
D11 0.03000000 sec
TD0 1
SFO1 125.8131151 MHz
NUC1 13C
P0 3.33 usec
P1 10.00 usec
PLW1 103.61000061 W
SFO2 500.3020012 MHz
NUC2 1H
CPDPRG[2 waltz65
PCPD2 80.00 usec
PLW2 13.35999966 W
PLW12 0.30400079 W
PLW13 0.15236519 W
F2 - Processing parameters
SI 32768
SF 125.8005211 MHz
WDW EM
SSB 0
LB 1.00 Hz
GB 0
PC 1.40
210 200 190 180 170 160 150 140 130 120 110 100 90 80 70 60 50 40 30 20 10 0 ppm

^1H NMR ($CDCl_3$)

S4

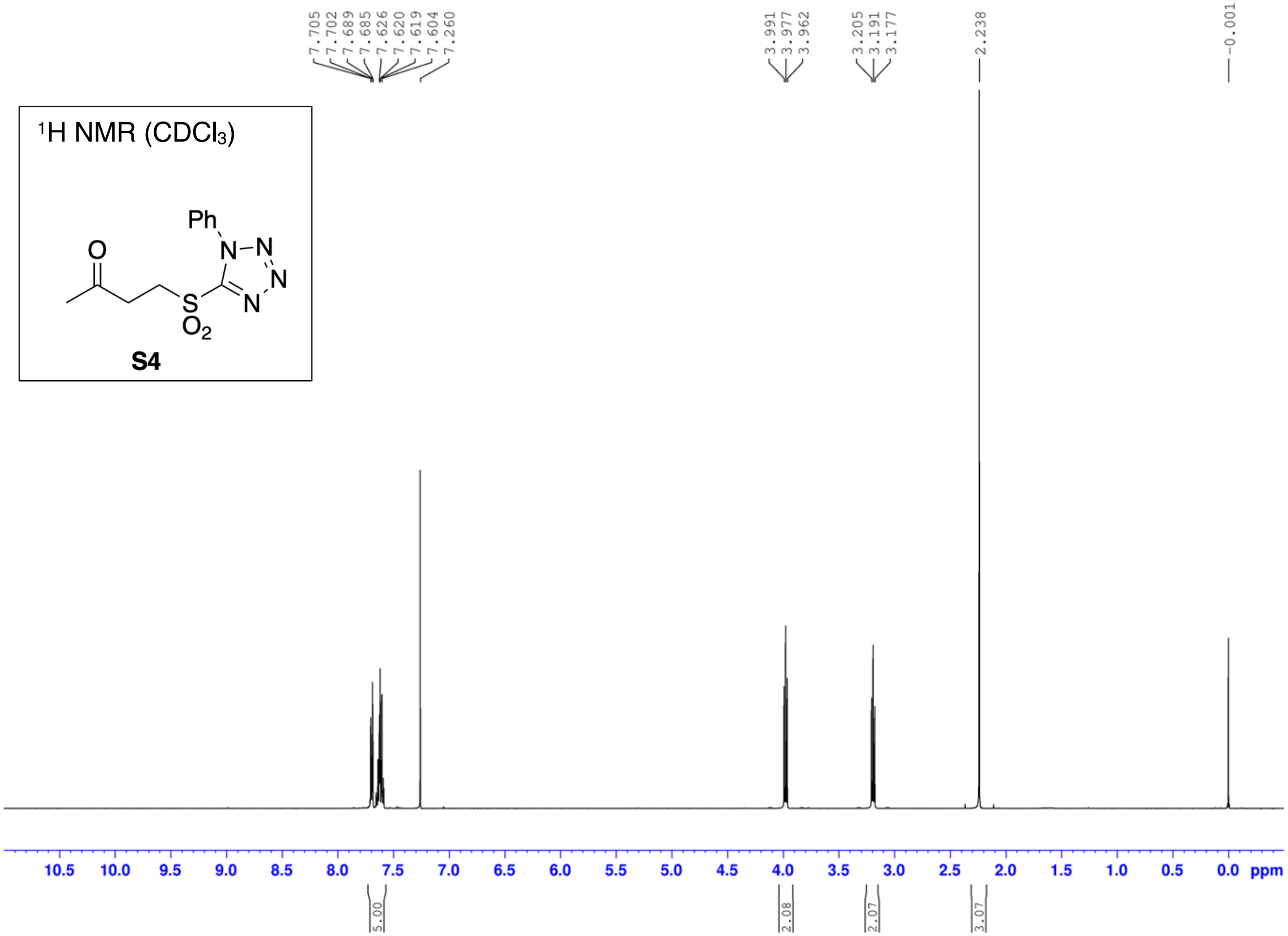

Current Data Parameters
NAME JNH_03_162
EXPNO 1
PROCNO 1

F2 - Acquisition Parameters
Date_ 20220823
Time 13.43 h
INSTRUM Avance NEO 500
PROBHD Z113652_0071 (
PULPROG zg30
TD 65536
SOLVENT CDCl3
NS 16
DS 2
SWH 10000.000 Hz
FIDRES 0.305176 Hz
AQ 3.2767999 sec
RG 101
DW 50.000 usec
DE 10.45 usec
TE 300.0 K
D1 1.00000000 sec
TD0 1
SFO1 500.3030894 MHz
NUC1 1H
P0 4.00 usec
P1 12.00 usec
PLW1 13.35999966 W

F2 - Processing parameters
SI 65536
SF 500.3000121 MHz
WDW no
SSB 0
LB 0 Hz
GB 0
PC 1.00

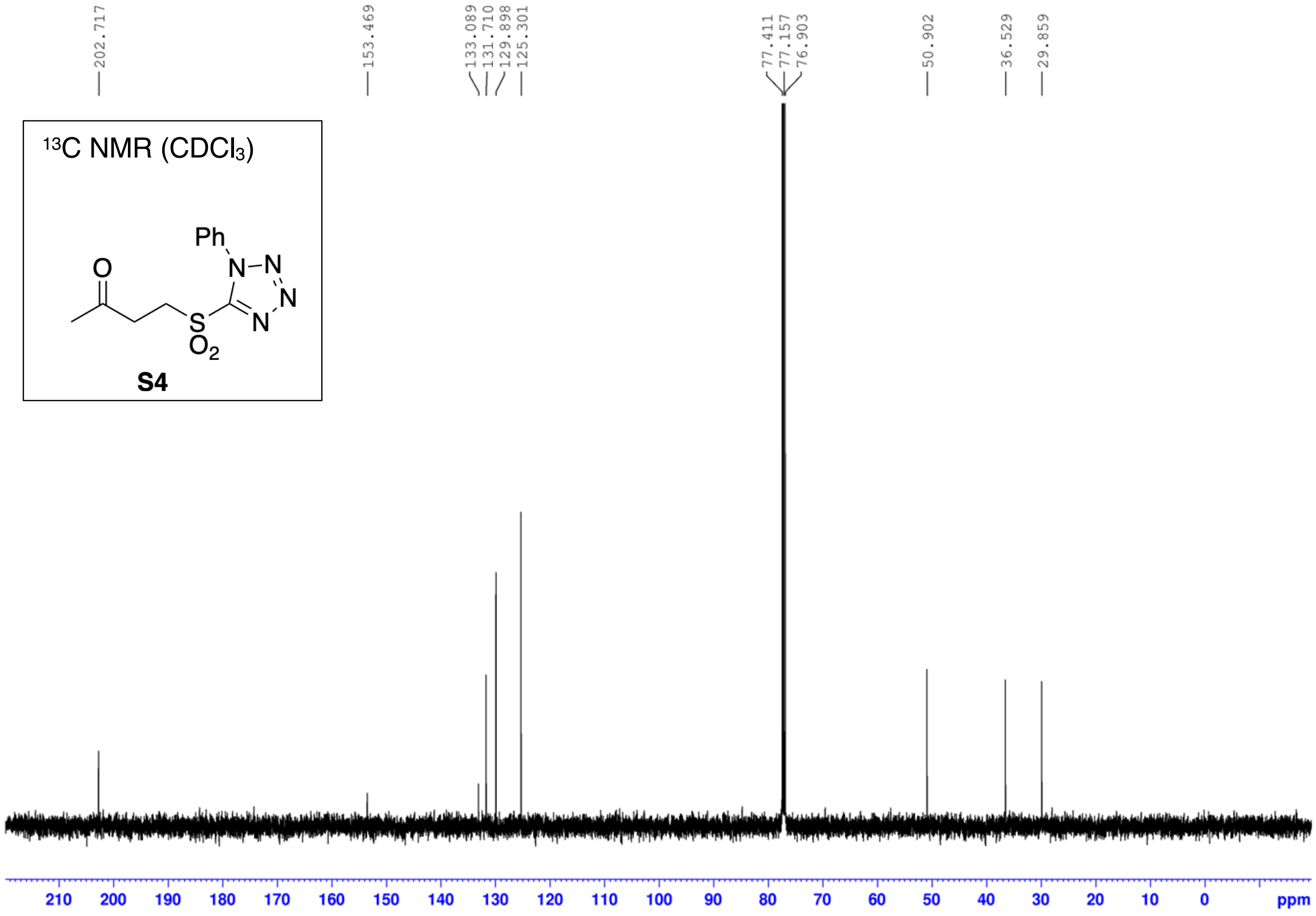


```
Current Data Parameters
NAME      JNH_03_162 13C
EXPNO                  1
PROCNO                 1

F2 - Acquisition Parameters
Date_           20220823
Time               13.50 h
INSTRUM   Avance NEO 500
PROBHD    Z113652_0071 (
PULPROG          zgpg30
TD                65536
SOLVENT           CDCl3
NS                   73
DS                    4
SWH           30120.482 Hz
FIDRES         0.919204 Hz
AQ            1.0878977 sec
RG                  101
DW               16.600 usec
DE                10.00 usec
TE                300.0 K
D1           2.00000000 sec
D11          0.03000000 sec
TD0                   1
SFO1        125.8131151 MHz
NUC1                13C
P0                 3.33 usec
P1                10.00 usec
PLW1       103.61000061 W
SFO2        500.3020012 MHz
NUC2                 1H
CPDPRG[2          waltz65
PCPD2             80.00 usec
PLW2        13.35999966 W
PLW12        0.30400079 W
PLW13        0.15236519 W

F2 - Processing parameters
SI                32768
SF          125.8005175 MHz
WDW                  EM
SSB                   0
LB                 1.00 Hz
GB                    0
PC                 1.40
```

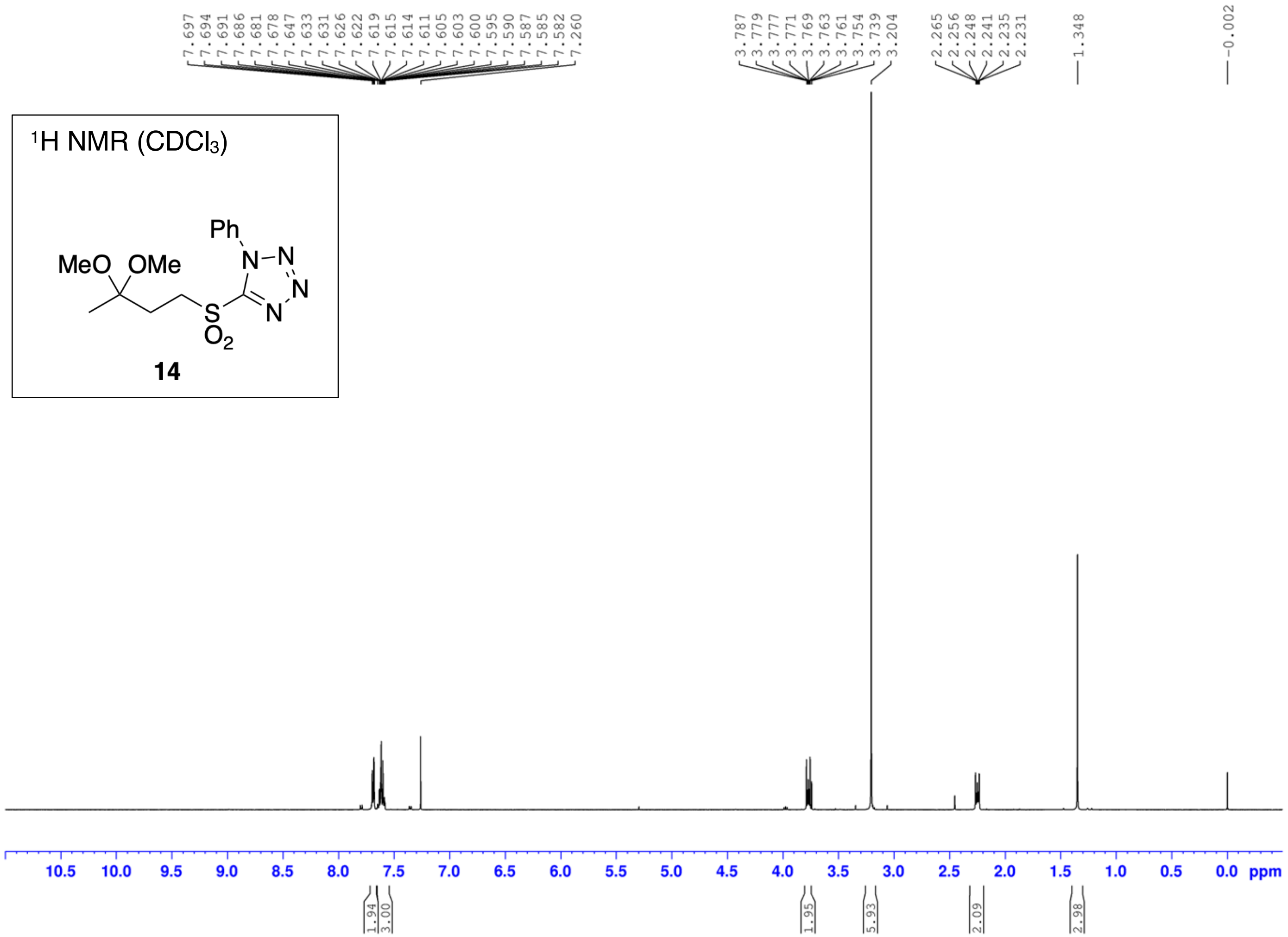


```
Current Data Parameters
NAME            JNH_03_163
EXPNO                    2
PROCNO                   1

F2 - Acquisition Parameters
Date_             20220824
Time                 10.59 h
INSTRUM   Avance NEO 500
PROBHD    Z113652_0071 (
PULPROG               zg30
TD                   65536
SOLVENT              CDCl3
NS                      16
DS                       2
SWH              10000.000 Hz
FIDRES            0.305176 Hz
AQ               3.2767999 sec
RG                     101
DW                  50.000 usec
DE                   10.45 usec
TE                   300.0 K
D1              1.00000000 sec
TD0                      1
SFO1           500.3030894 MHz
NUC1                    1H
P0                    4.00 usec
P1                   12.00 usec
PLW1           13.35999966 W

F2 - Processing parameters
SI                   65536
SF             500.3000121 MHz
WDW                     no
SSB                      0
LB                       0 Hz
GB                       0
PC                    1.00
```

^{13}C NMR ($CDCl_3$)

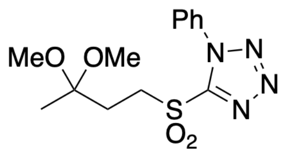

14

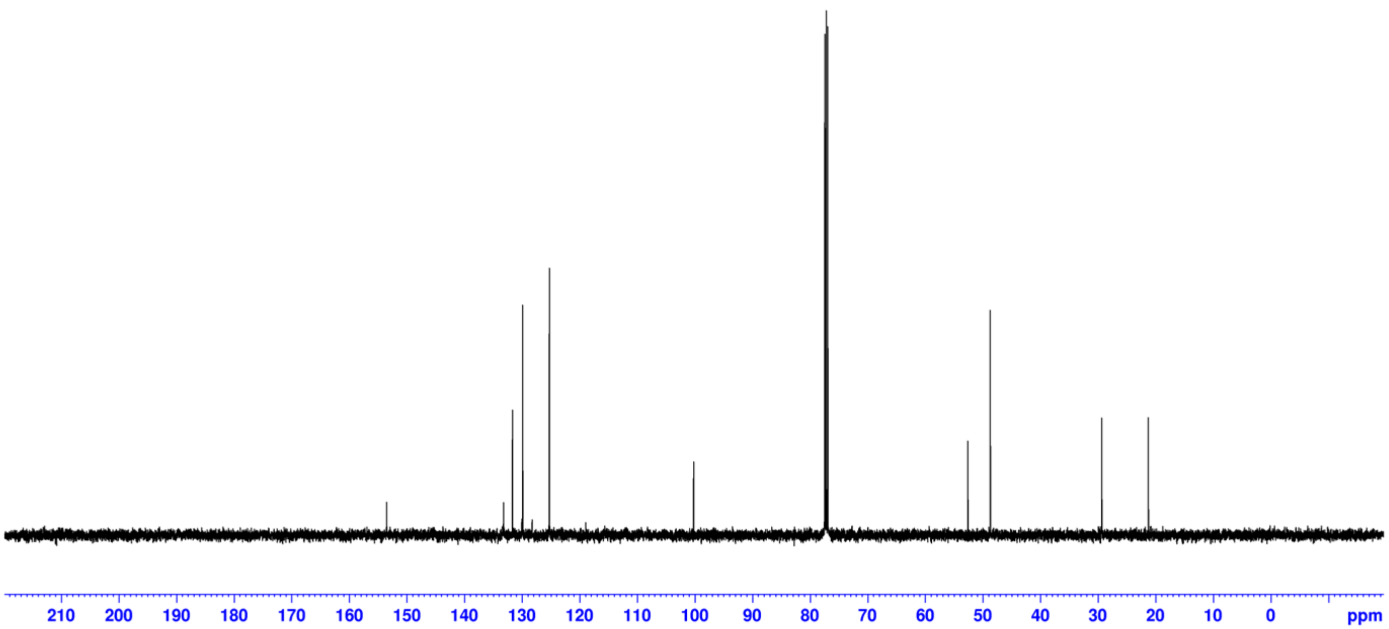


```
Current Data Parameters
NAME      JNH_03_163 13C
EXPNO                  2
PROCNO                 1

F2 - Acquisition Parameters
Date_           20220824
Time               11.07 h
INSTRUM   Avance NEO 500
PROBHD    Z113652_0071 (
PULPROG          zgpg30
TD                65536
SOLVENT           CDCl3
NS                   57
DS                    4
SWH           30120.482 Hz
FIDRES         0.919204 Hz
AQ            1.0878977 sec
RG                  101
DW               16.600 usec
DE                10.00 usec
TE                300.0 K
D1           2.00000000 sec
D11          0.03000000 sec
TD0                   1
SFO1        125.8131151 MHz
NUC1                13C
P0                 3.33 usec
P1                10.00 usec
PLW1       103.61000061 W
SFO2        500.3020012 MHz
NUC2                 1H
CPDPRG[2        waltz65
PCPD2             80.00 usec
PLW2        13.35999966 W
PLW12        0.30400079 W
PLW13        0.15236519 W

F2 - Processing parameters
SI                32768
SF          125.8005184 MHz
WDW                  EM
SSB                   0
LB                 1.00 Hz
GB                    0
PC                 1.40
```
